# TASCI—transcutaneous tibial nerve stimulation in patients with acute spinal cord injury to prevent neurogenic detrusor overactivity: protocol for a nationwide, randomised, sham-controlled, double-blind clinical trial

**DOI:** 10.1136/bmjopen-2020-039164

**Published:** 2020-08-13

**Authors:** Veronika Birkhäuser, Martina D Liechti, Collene E Anderson, Lucas M Bachmann, Sarah Baumann, Michael Baumberger, Lori A Birder, Sander M Botter, Silvan Büeler, Célia D Cruz, Gergely David, Patrick Freund, Susanne Friedl, Oliver Gross, Margret Hund-Georgiadis, Knut Husmann, Xavier Jordan, Miriam Koschorke, Lorenz Leitner, Eugenia Luca, Ulrich Mehnert, Sandra Möhr, Freschta Mohammadzada, Katia Monastyrskaya, Nikolai Pfender, Daniel Pohl, Helen Sadri, Andrea M Sartori, Martin Schubert, Kai Sprengel, Stephanie A Stalder, Jivko Stoyanov, Cornelia Stress, Aurora Tatu, Cécile Tawadros, Stéphanie van der Lely, Jens Wöllner, Veronika Zubler, Armin Curt, Jürgen Pannek, Martin W G Brinkhof, Thomas M Kessler

**Affiliations:** 1 Department of Neuro-Urology, Balgrist University Hospital, University of Zürich, Zürich, Switzerland; 2 Swiss Paraplegic Research, Nottwil, Switzerland; 3 Department of Health Sciences and Medicine, University of Lucerne, Lucerne, Switzerland; 4 Medignition Inc., Research Consultants, Zürich, Switzerland; 5 Spinal Cord and Rehabilitation Medicine, Swiss Paraplegic Centre, Nottwil, Switzerland; 6 Neuro-Urology, University of Pittsburgh School of Medicine, Pittsburgh, Pennsylvania, USA; 7 Swiss Center for Musculoskeletal Biobanking, Balgrist Campus AG, Zürich, Switzerland; 8 Instituto de Investigação e Inovação em Saúde, Translational Neuro-urology Group, Universidade do Porto, Porto, Portugal; 9 Faculdade de Medicina, Departemento de Biomedicina, Unidade de Biologia Experimental, Universidade do Porto, Porto, Portugal; 10 Spinal Cord Injury Center, Balgrist University Hospital, University of Zürich, Zürich, Switzerland; 11 Clinic of Neurorehabilitation and Paraplegiology, REHAB Basel, Basel, Switzerland; 12 Spinal Cord Injury Department, Clinique romande de réadaptation, Sion, Switzerland; 13 Urology Research Laboratory, DBMR, University of Bern, Bern, Switzerland; 14 Department of Gastroenterology and Hepatology, University Hospital Zürich, Zürich, Switzerland; 15 Institute for Regenerative Medicine, University of Zürich, Zürich, Switzerland; 16 Department of Trauma, University Hospital Zürich, Zürich, Switzerland; 17 Neuro-Urology, Swiss Paraplegic Centre, Nottwil, Switzerland; 18 Department of Radiology, Balgrist University Hospital, Zürich, Switzerland; 19 Department of Urology, Inselspital University Hospital Bern, Bern, Switzerland

**Keywords:** neuro-urology, urinary incontinences, neurological injury, rehabilitation medicine, bladder disorders

## Abstract

**Introduction:**

Neurogenic lower urinary tract dysfunction (NLUTD), including neurogenic detrusor overactivity (NDO) and detrusor sphincter dyssynergia, is one of the most frequent and devastating sequelae of spinal cord injury (SCI), as it can lead to urinary incontinence and secondary damage such as renal failure. Transcutaneous tibial nerve stimulation (TTNS) is a promising, non-invasive neuromodulatory intervention that may prevent the emergence of the C-fibre evoked bladder reflexes that are thought to cause NDO. This paper presents the protocol for TTNS in acute SCI (TASCI), which will evaluate the efficacy of TTNS in preventing NDO. Furthermore, TASCI will provide insight into the mechanisms underlying TTNS, and the course of NLUTD development after SCI.

**Methods and analysis:**

TASCI is a nationwide, randomised, sham-controlled, double-blind clinical trial, conducted at all four SCI centres in Switzerland. The longitudinal design includes a baseline assessment period 5–39 days after acute SCI and follow-up assessments occurring 3, 6 and 12 months after SCI. A planned 114 participants will be randomised into verum or sham TTNS groups (1:1 ratio), stratified on study centre and lower extremity motor score. TTNS is performed for 30 min/day, 5 days/week, for 6–9 weeks starting within 40 days after SCI. The primary outcome is the occurrence of NDO jeopardising the upper urinary tract at 1 year after SCI, assessed by urodynamic investigation. Secondary outcome measures assess bladder and bowel function and symptoms, sexual function, neurological structure and function, functional independence, quality of life, as well as changes in biomarkers in the urine, blood, stool and bladder tissue. Safety of TTNS is the tertiary outcome.

**Ethics and dissemination:**

TASCI is approved by the Swiss Ethics Committee for Northwest/Central Switzerland, the Swiss Ethics Committee Vaud and the Swiss Ethics Committee Zürich (#2019-00074). Findings will be disseminated through peer-reviewed publications.

**Trial registration number:**

NCT03965299.

**Strengths and limitations of this study**

- This study is a nationwide, randomised, sham-controlled, double-blind clinical trial conducted at all four spinal cord injury (SCI) centres in Switzerland.
- Transcutaneous tibial nerve stimulation (TTNS) is a well-tolerated, low-risk, non-invasive intervention.
- The inpatient setting facilitates study procedures and monitoring that maximise intervention adherence.
- Secondary outcomes provide valuable information regarding the impact of TTNS on additional factors that are relevant to patients as well as clinicians, and also facilitate targeted analyses aimed at better understanding the mechanism underlying TTNS therapy, and the development of neurogenic lower urinary tract dysfunction after SCI.
- In comparison to beginning immediately after SCI, the intervention start at 29–40 days after SCI (in order to complete all baseline assessments) may modestly reduce the potential for neuroplasticity and the maximum achievable intervention effect.

## Introduction

Most patients with spinal cord injury (SCI) progressively develop neurogenic lower urinary tract dysfunction (NLUTD),^1^ which is one of the most devastating sequelae of SCI. Recovery of lower urinary tract (LUT) function is often identified as a top patient concern after SCI, and sometimes is even prioritised above walking.^2–4^ The consequences of NLUTD, including urinary incontinence, dependence on catheters and recurrent urinary tract infections, negatively impact quality of life (QoL).^5–7^ On a neurological level, intact pathways between the brain and the LUT are required for the complex multilevel neural network governing the LUT to function properly.^8^ SCI is thought to trigger neuroplastic changes in C-fibre-mediated spinal reflex pathways in the spinal cord, which lead to the emergence of aberrant micturition reflexes.^9 10^ Thus, within a few weeks after acute SCI, most patients start experiencing involuntary bladder contractions, neurogenic detrusor overactivity (NDO), which can cause urinary incontinence.^8^ Furthermore, NDO, in combination with detrusor sphincter dyssynergia (DSD) and/or low bladder compliance, can produce high intravesical pressure, which is a relevant risk factor for renal failure.^11 12^ NDO also predisposes patients to vesicoureterorenal reflux, which can endanger the upper urinary tract (UUT).^8 13^ Accordingly, the current urological management approach is centred around protecting the UUT and treating long-term complications of NLUTD.^13 14^ Antimuscarinic medication and intradetrusor onabotulinumtoxin A injections are the first-line and second-line treatments for NDO, but both treatments are often discontinued due to limited effectiveness and bothersome side effects.^15–17^ If conventional conservative and minimally invasive therapies fail, the only remaining options are more invasive treatments which are associated with substantial morbidity, such as bladder augmentation and urinary diversion.^18 19^ While neurourological treatment of patients with SCI has greatly improved during the last decades, it is only applied when NLUTD is already established with little chance of recovery.

Here, we present the protocol for a randomised controlled trial (RCT) that aims at producing evidence for the use of non-invasive electrical stimulation (neuromodulation) to prevent the development of NDO. Early neuromodulatory therapy has the potential to impede or alter neuroplasticity, preventing the sensitisation of ‘silent’ C fibres and the formation of spinal reflex pathways thought to be responsible for NDO. Indeed, in a pilot study, bilateral sacral neuromodulation during the early phase of SCI prevented the development of NDO and also improved erectile and bowel function.^20^ Furthermore, in animal models, early neuromodulatory interventions after SCI can modulate the micturition reflex^21^ and inhibit detrusor overactivity.^22^ In humans, non-invasive neuromodulatory techniques such as tibial nerve stimulation^23^ and transcutaneous electrical nerve stimulation (TENS)^24^ have shown promise in a treatment context^25–28^ and warrant well-designed RCTs. Transcutaneous tibial nerve stimulation (TTNS) has generated interest, as a neuromodulatory treatment that is completely non-invasive (current is applied through surface electrodes) with no known safety risks, easy to apply and well accepted by patients. A randomised pilot trial in patients with acute SCI concluded that TTNS was feasible and safe in an inpatient rehabilitation setting.^29^ Furthermore, at the end of the study, the participants in the intervention group had lower detrusor pressures and fewer developed DSD compared with the sham group,^30^ suggesting that TTNS could alter the course of NLUTD. We hypothesise that TTNS applied in the acute phase after SCI will prevent the emergence of C-fibre evoked bladder reflexes, thereby the development of NDO, and avert UUT damage and urinary incontinence. TTNS in acute SCI (TASCI) aims to provide evidence regarding the clinical efficacy of TTNS as a preventative measure for NDO, along with additional insight into the mechanism underlying TTNS and the course of NLUTD development during the first year after SCI. We operationalised this RCT as a population-based study in the context of specialised rehabilitation for SCI in Switzerland.

## Methods and analyses

This protocol follows the recommendations of the Standard Protocol Items: Recommendations for Interventional Trials (SPIRIT) Statement (online supplementary 1).^31^

10.1136/bmjopen-2020-039164.supp1 Supplementary data

### Study design and setting

TASCI is a nationwide, randomised, sham-controlled, double-blind clinical trial investigating the effects of TTNS in patients with acute SCI.^32^ The intervention will take place during the inpatient rehabilitation stay in one of the four SCI centres in Switzerland (Basel: REHAB Basel, Nottwil: Swiss Paraplegic Center, Sion: Clinique romande de réadaptation, and Zürich: Balgrist University Hospital). Furthermore, TASCI is nested in the inception cohort of the prospective Swiss Spinal Cord Injury (SwiSCI) cohort study.^33^

**Figure 1:**
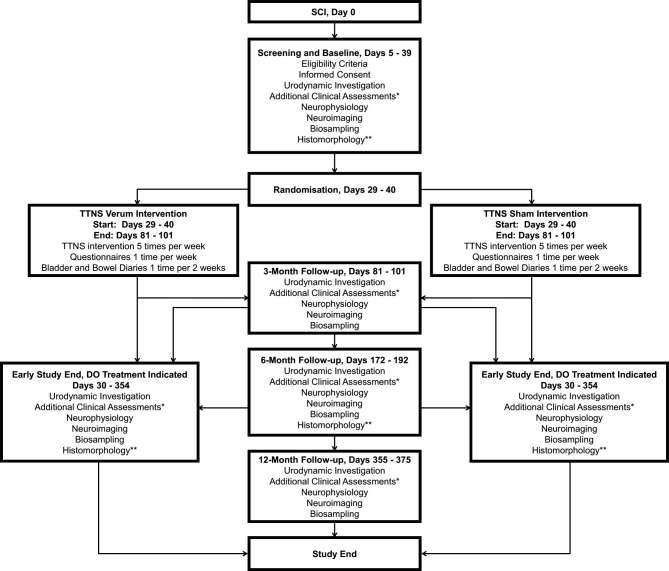
Trial flow chart. Safety data will be recorded on a continuous basis the entire time the participant is enrolled in the study. *Additional clinical assessments target urological and bowel function and symptoms, neurological structure and function, sexual function, spasticity, functional independence, quality of life and biochemical and molecular biomarkers in the urine, blood, stool and in bladder tissue (additional consent required for bladder biopsy). **Histomorphology is based on bladder biopsy which requires additional consent. DO, detrusor overactivity; SCI, spinal cord injury; TTNS, transcutaneous tibial nerve stimulation.

The TASCI schedule (figure 1) includes a screening and baseline period from days 5–39 after SCI diagnosis, followed by a 6–9 weeks intervention period that ends when all 3-month follow-up assessments have been completed. Three follow-up visits are planned: the 3-month visit (day 91±10), 6-month visit (day 182±10) and 12-month visit (day 365±10). The 1 year post-SCI measurement time point was chosen for the primary outcome variable because it represents the time when the patient’s situation is stable enough to start considering invasive treatment options.^14^ The additional time points were selected to maximise the chances of detecting treatment effects, as well as to coincide with routine follow-up assessments of patients with SCI.

### Participants

All patients with SCI admitted to rehabilitation in one of the participating centres are screened for eligibility using information from their routine clinical examinations. For a full listing of inclusion and exclusion criteria, refer to table 1. Eligible patients are then approached by an investigator or supervising clinician who obtains informed consent. For an English translation of the study information leaflet and declaration of consent, see online supplementary 2. Patients who consent undergo additional baseline assessments and are randomised after their eligibility for the trial has been confirmed.

10.1136/bmjopen-2020-039164.supp2 Supplementary data

**Table 1:** Transcutaneous tibial nerve stimulation in acute spinal cord injury (TASCI) eligibility criteria

Inclusion criteria	Exclusion criteria
Age ≥ 18 years Patients with acute SCI (traumatic SCI and sudden onset (<7 days) non-traumatic SCI), within 40 days after injury Patients with acute SCI at cervical or thoracic level Willingness to take part and follow the requirements of the protocol during TASCI (up to 1 year after SCI): No percutaneous tibial nerve stimulation No functional electrical stimulation (FES) apart from upper limb FES No electrical muscle stimulation Informed consent	Contraindications to the investigational product Treatment with antimuscarinics or mirabegron DO with contractions greater than 40 cm H_2_O at a bladder filling volume of less than 500 mL at baseline visit Known or suspected non-adherence, drug or alcohol abuse Inability to follow the procedures of the study, for example, due to language problems, psychological disorders, dementia, and so on, of the participant Participation in another study with investigational drug or product within the 30 days preceding and during the present study Neuromodulation treatment for urological or bowel indication in the last 6 months or ongoing Botulinum toxin injections in the detrusor and/or urethral sphincter in the last six months Bilaterally absent tibial nerve compound muscle action potential (amplitude < 1 mV) Women who are pregnant or breast feeding Individuals especially in need of protection (according to Research with Human Subjects published by the Swiss Academy of Medical Sciences (www.samw.ch/en/News/News.html) Enrolment of the investigator, their family members, employees and other dependent persons Pre-existing or concomitant medical condition apart from SCI that might pose a safety issue or would interfere with interpretation of study results or study conduct (eg, Parkinson’s disease, neurodegenerative disorders including multiple sclerosis and amyotrophic lateral sclerosis, urological malignancies)

Urinary tract infection (UTI) is a transitory exclusion criterion for all functional assessments, including urodynamics. Before undergoing functional assessments, participants are screened for UTIs and haematuria. If a UTI is identified, treatment is initiated, and the functional assessments are rescheduled once the participant has been symptom free for at least 3 days.

DO, detrusor overactivity; SCI, spinal cord injury.

### Randomisation and blinding

Participants are randomly allocated with a 1:1 ratio into TTNS verum or TTNS sham stimulation groups, using computer-generated permuted block randomisation lists with varying block sizes. The randomisation lists are stratified according to study centre and the primary prognostic indicator, lower extremity motor score (LEMS) from the International Standards for Neurological Classification of SCI assessment.^34^ Prognostic models have shown that LEMS measured within 40 days of SCI is a reliable predictor of urinary continence and complete bladder emptying 1 year after SCI.^35^ Based on this model, LEMS cut-offs of <12, 12–37 and >37 were chosen, which correspond to a population-average estimated probability of <20%, 20%–80% and >80% for urinary continence and complete bladder emptying at 1 year after SCI.^35^ The allocation sequence and assignment of participants are centrally coordinated, and performed via the database software (Research Electronic Data Capture (REDCap)).^36 37^ To safeguard the allocation concealment, the randomisation table will be password secured and only accessible to investigators responsible for randomisation and application of the TTNS intervention.

To maintain blinding of the participants, care providers and outcome assessors, throughout the duration of the participants’ involvement in the study, the randomisation and TTNS intervention will be performed by trained operators, who are not involved in the clinical management. Additionally, the verum and sham TTNS set-ups are identical in terms of appearance, application procedure and intervention duration, and all participants receive the same information.

### Intervention

**Figure 2:**
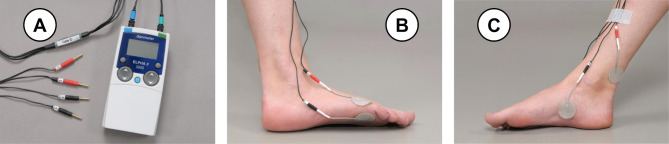
Transcutaneous tibial nerve stimulation (TTNS) intervention. Pictures of the TTNS device, cables and electrode placements. (A) The TTNS device has two channels that can be adjusted independently. Electrodes are placed in a specified, standardised order (1–4). (B) Lateral view of the foot, showing the electrode placement on the dorsal bone and plantar fat pad of the fifth metatarsophalangeal joint. (C) Medial view of the foot, electrodes are placed 4–5 cm proximal and posterior to the medial malleolus, as well as in the longitudinal arch of the foot, distal to, and in line with, the medial malleolus.

A commercially available TENS device (ELPHA II 3000, CE 0543 Certification, FH Service, Odense, Denmark) is used for both the TTNS verum and sham intervention (figure 2). The current frequency (20 Hz) and pulse width (200 μs) are preprogrammed. The current amplitude, which can be set between 0 and 100 mA, is manually adjusted for each participant during the initial setup period. Each TTNS session starts with a preparatory phase where the participant is asked to lie in a comfortable supine position and a pillow or blanket is placed on the lower part of the leg to restrict the participant’s view of their foot. Four electrodes are placed according to a prespecified order on the foot and lower leg. The first electrode is placed 4–5 cm above and 4–5 cm posterior to the medial malleolus. The next two electrodes are placed around the fifth metatarsophalangeal joint, one dorsally over the bone, and one on the plantar fat pad. A final electrode is placed distal to, and in line with, the medial malleolus in the longitudinal arch of the foot (figure 2). During an initial test period of several minutes, sensory and motor thresholds (if applicable) are assessed, current amplitudes are adjusted and the operator is carefully observing foot movement, that is, flexion or fanning of the big toe or any other motor response. The operator may reposition electrodes to optimise the electrical stimulation set-up. After the correct positioning of the electrodes has been verified by observation of the appropriate motor response,^23^ the stimulation amplitude is reduced to a submotor level, defined as 0.5–1.0 mA below the motor threshold. In the sham set-up, a cable with an interposed 1000 Ω resistance allows a current amplitude to be displayed and changed on the device without providing electrical stimulation to the participant.

The TTNS intervention is performed for 30 min/day, 5 days/week, starting within 40 days after SCI and ending after all 3-month follow-up assessments have been completed (total intervention period: 6–9 weeks). The majority of the participants are expected to be inpatients for the duration of the intervention, a setting that maximises participant adherence. The application of the TTNS intervention by designated and trained personnel ensures that the treatment is applied correctly while blinding is maintained. In addition, the remaining follow-up visits are part of standardised SCI care in all four participating centres, limiting the potential loss to follow-up, and ensuring that the participants receive necessary ancillary care.

Participants may be withdrawn when the study protocol is not followed, a health problem occurs with a suspected link to the stimulation, if a severe health condition/illness makes an intensive outpatient treatment or new hospitalisation necessary, consent is withdrawn, or if a pregnancy is detected. Any concomitant treatment and medication intake will be carefully documented, so that the information can be used in secondary analyses.

### Outcomes

The primary outcome is the occurrence of NDO jeopardising the UUT up to 12 months after SCI, that is, at 1 year after SCI or any earlier time point if NDO treatment is needed. This outcome is a composite measure including: NDO with an amplitude ≥40 cm H_2_O identified during urodynamic investigation; or else the initiation of NDO treatment with antimuscarinics and/or intradetrusor onabotulinumtoxin A injections. Urodynamics will be performed before starting TTNS (within 40 days after SCI) at follow-up visits 3 months, 6 months and 1 year after SCI, or at any earlier time point if there is reason to believe that the participant has developed NDO, before starting NDO treatment.

Secondary outcomes (table 2) were selected to further evaluate the effects of the TTNS intervention, and include clinical outcomes such as differential changes in LUT and bowel function and symptoms, sexual function, neurological function, as well as functional independence and QoL. Moreover, the evaluation of differential changes in brain and spinal cord function as well as corticospinal integrity, and biomarkers in the urine, blood, stool and bladder tissue will also provide insight into the mechanism of action underlying the TTNS intervention. All secondary outcomes may also be used to better understand the pathogenesis of NLUTD after SCI. Information collected will provide a detailed description of the disease course, and when possible, inform prognostic modelling and address causal questions. Finally, adverse events will be recorded for the duration of the participant's enrolment in the study to evaluate the safety of TTNS. Table 2 shows an overview of the secondary outcome measurement tools and assessment periods, and secondary outcome parameters are listed in detail in online supplementary 3.

10.1136/bmjopen-2020-039164.supp3 Supplementary data

**Table 2:** Secondary outcome measurement tools and time of assessment

Outcome and assessment tool	Assessment periods
Occurrence of neurogenic DO jeopardising the upper urinary tract over a 1-year time course (time-to-event analysis)	
Urodynamic investigation	S&B, 3M, 6M, 12M
Clinical assessment	S&B, 3M, 6M, 12M
Differential changes in lower urinary tract function	
Urodynamic investigation	S&B, 3M, 6M, 12M
Differential changes in lower urinary tract symptoms	
Bladder diary	S&B, 3M, 6M, 12M; TTNS: biweekly
International Prostate Symptom Score (IPSS)	S&B, 3M, 6M, 12M; TTNS: weekly
Urinary Symptom Profile (USP)	S&B, 3M, 6M, 12M; TTNS: weekly
Differential changes in neurological structure and function	
Neurourological physical examination	S&B, 3M, 6M, 12M
International Standards for Neurological Classification of Spinal Cord Injury (ISNCSCI) assessment	S&B, 3M, 6M, 12M
Nerve conduction studies (NCS)	S&B, 3M, 6M, 12M
Electromyography (EMG)	S&B, 3M, 6M, 12M
Electroencephalography (EEG)	S&B, 3M, 6M, 12M
Transcranial magnetic stimulation (TMS)	S&B, 3M, 6M, 12M
Magnetic resonance imaging (MRI)	S&B, 3M, 6M, 12M
Differential changes in bowel and pelvic floor function	
Rectal sensitivity examination	S&B, 3M, 6M, 12M
Anorectal manometry	S&B, 3M, 6M, 12M
Barostat assessment	S&B, 3M, 6M, 12M
Differential changes in bowel symptoms	
Bowel diary	S&B, 3M, 6M, 12M; TTNS: biweekly
Neurogenic Bowel Dysfunction Score (NBD)	S&B, 3M, 6M, 12M; TTNS: weekly
Differential changes in sexual function	
Female Sexual Function Index (FSFI)	S&B, 3M, 6M, 12M
International Index of Erectile Function (IIEF)	S&B, 3M, 6M, 12M
Clinical assessment	S&B, 3M, 6M, 12M
Differential changes in spasticity	
Modified Ashworth Scale (MAS)	S&B, 3M, 6M, 12M
Spinal Cord Injury (SCI) Spasticity Evaluation Tool (SCI-SET)	S&B, 3M, 6M, 12M; TTNS: weekly
Differential changes in functional independence	
Spinal Cord Independence Measure (SCIM) III	S&B, 3M, 6M, 12M
Differential changes in quality of life	
International Prostate Symptom Score (IPSS)	S&B, 3M, 6M, 12M; TTNS: weekly
Qualiveen	S&B, 3M, 6M, 12M; TTNS: weekly
Differential changes in biochemical and molecular biomarkers in the urine, blood and stool, as well as in the bladder tissue	
Histomorphology	S&B 6M
Enzyme-linked immunosorbent assay (ELISA)	S&B, 3M, 6M, 12M
Western immunoblotting	S&B, 3M, 6M, 12M
Reverse transcription polymerase chain reaction (RT-PCR)	S&B, 3M, 6M, 12M
Microbiome analysis	S&B, 3M, 6M, 12M
Safety of TTNS	
Adverse events and serious adverse events as assessed by clinical record check and patient interview	Any time

Assessment periods are referenced to the date of SCI diagnosis and include: screening and baseline (S&B; days 5–39), 3-month follow-up (3M; days 91±10), 6-month follow-up (6M; days 182±10) and 12-month follow-up (12M; days 365±10). Additionally, some surveys are administered weekly during the transcutaneous tibial nerve stimulation (TTNS) treatment period (TTNS: weekly), and the bowel and bladder diaries are administered once every 2 weeks during the treatment period (TTNS: biweekly).

DO, detrusor overactivity.

Generally, the outcome assessments are administered at screening and baseline as well as during each of the three follow-up visits (figure 1). One notable exception is the bladder biopsy, which will take place at baseline and the 6-month follow-up visit, and only with additional consent. Moreover, during the intervention period, participants will complete some of the questionnaires once per week, and the bladder and bowel diaries once every 2 weeks. Withdrawal from the study can occur at any time if treatment for NDO is indicated. If withdrawal occurs, all study completion assessments should be performed whenever possible. Furthermore, the trial is nested in the SwiSCI cohort study, so observation of the participants will continue through the cohort study after the end of TASCI. SwiSCI can provide long-term, open-label outcome data regarding morbidity, activities and participation (eg, labour market participation), QoL and life expectancy.

### Sample size

Presuming a proportion of spontaneous success among the sham group of 0.15,^23^ 114 participants (57 verum, 57 sham) need to be included to detect a difference of 0.25 in the proportion of participants who develop NDO, and thereby to correctly reject the null hypothesis with a probability (power) of 0.8. The type I error probability associated with the test of this null hypothesis is 0.05. Since the primary outcome variable is binary, this sample size analysis included a continuity correction to avoid type I error inflation.

Using prospective data collected by the SwiSCI inception cohort for the years 2014–2016 and applying a modified version of the TASCI inclusion/exclusion criteria, a total of 193 eligible patients are predicted during the 3-year recruitment period of TASCI. Anticipating a recruitment rate of 66%, this implies that the requisite 114 participants can be included within 3 years. Expected participant numbers per centre during the study period are 20 for Basel, 42 for Nottwil, 13 for Sion and 39 for Zürich. Moreover, these data imply that participants who withdraw or drop out can be replaced.

### Data collection and management

All investigators are responsible for complete and correct data entry. Data will be recorded in electronic case report forms using an internet-based, secure database—REDCap,^36 37^ which fulfils the good clinical practice (GCP) requirements and is hosted at Balgrist University Hospital. Participants will be assigned a unique 10-digit identification number, and personal data (eg, name) are not entered into the main study database. Confidentiality will be ensured by restricting the access to the trial database to only a minimal number of people, and by pseudonymisation of the data before analyses. The database includes built-in quality control mechanisms, such as range-restricted data entry, and identification of missing data. A monitoring system clearly indicates that an expected assessment is late or missing. Additionally, a manual check is performed of each dataset after it is closed and marked as complete, first by a research assistant at the study site, then by a study coordinator.

### Statistical analyses

The primary analysis will be an intention-to-treat analysis. The general statistical methodology for primary and secondary outcomes comprises basic descriptive statistics and multivariable regression analyses. Continuous variables will be tested for normality, and displayed as either means and SD or medians and ranges; categorical variables will be displayed as frequencies and proportions. Multivariable regression techniques will be employed to control for possible confounding due to unequal distribution of baseline parameters across the trial arms, as well as systematic variation in outcomes across RCT strata, that is, centre and LEMS. Longitudinal analysis techniques (eg, mixed-effect models, structural equation models for growth and time-to-event models) will be used to account for repeated measures, facilitating time-updated covariate adjustment and appropriately modelling trends with respect to time.

The primary outcome ‘the occurrence of NDO jeopardising the UUT 1 year after SCI’ is a binary response variable, which will be described using proportions with 95% binomial CIs and analysed using logistic regression, thus expressing treatment effect sizes as ORs (with 95% CIs). Sensitivity analyses will include a time-to-event analysis to evaluate the impact of TTNS on the risk of early drop-out or preterm endpoints (<1 year). Protocol non-adherence, early withdrawals and loss to follow-up will be examined and used to inform per-protocol sensitivity analyses. Subgroup analyses stratified on LEMS or study centre may be performed if an intervention–subgroup interaction is found, with the understanding that these analyses will likely be underpowered. In cases where outcome data are used in an observational context, usually to examine prognosis or NLUTD aetiology, causal claims will be supported by an underlying theoretical framework.

Multiple imputation will be used when appropriate to account for missing data in the predictor variables.^38^ Pattern mixture models may be applied to address missing outcome data for different scenarios of the missing data process.^39^ Sensitivity analysis will be used to evaluate the robustness of the chosen imputation strategy. Any sensitivity analyses added after the breaking of the blind (post-hoc sensitivity analyses) will be clearly identified as such in the trial report.

No interim analyses are planned. In the event that severe clinical or neurological deterioration is detected in more than one subject, or on the recommendation of the study monitoring board following a serious adverse event, the study will be suspended until a comprehensive safety review has been completed. If the trial is suspended or halted, an interim analysis will be performed.

### Quality assurance and safety oversight

Monitoring will be carried out by the Unit for Clinical and Applied Research at Balgrist University Hospital. Monitoring visits will occur at each study site before the start of the study, after 3-month follow-up assessments have been completed for the first participant, and then annually thereafter, with a final closeout visit at study end. The site qualification and initiation visit(s) will assure that the study site has the appropriate facilities and personnel, and that the personnel are able to conduct the study according to protocol. Routine visits will focus on source document verification, in particular documents related to eligibility, consent, key outcome parameters, and ensure the documentation and reporting of adverse events occurred in accordance with all legal, ethical and protocol-specific requirements.

### Patient and public involvement

Feedback on the intervention procedure was obtained from patients and healthy volunteers during the piloting phase and used to refine the study protocol. All participants will receive a study update newsletter annually.

## Ethics and dissemination

### Ethics

TASCI will be conducted in accordance with the protocol and the principles contained in the current version of the Declaration of Helsinki, the GCP guidelines issued by the ICH (International Conference on Harmonisation of technical requirements for registration of pharmaceuticals for human use), the European Regulation on medical devices 2017/745 and the ISO Norm 14 155 and ISO 14971, the Swiss Law and Swiss regulatory authority’s requirements. The study has been approved by the Swiss Ethics Committee for Northwest/Central Switzerland, the Swiss Ethics Committee Vaud and the Swiss Ethics Committee Zürich (2019-00074) and has been registered at ClinicalTrials.gov (NCT03965299). The ethics committees and regulatory authorities will receive annual safety and interim reports and be informed about study end. All investigators will respect the participant’s right to confidentiality, and adhere to the Swiss Federal Act on Data Protection. There are no conflicts of interest.

### Dissemination

The study results will be published in open-access peer-reviewed journals, and authorship will be defined according to the recommendations of the International Committee of Medical Journal Editors. All persons wishing to publish using TASCI data must submit a project plan to the TASCI steering committee for approval before the data are released for analysis. Datasets and statistical coding will be deposited in a public repository, as long as participant privacy is not compromised. Results will be presented at conferences of relevant societies such as the American Urological Association, Deutschsprachige Medizinische Gesellschaft für Paraplegie, European Association of Urology, International Continence Society, International Neuro-Urology Society and International Spinal Cord Society. Furthermore, these societies will be used as platforms to advocate the implementation of the study findings in daily clinical practice and incorporation into clinical guidelines. Dissemination among persons with SCI will be achieved through the newsletters Paraplegie and Paracontact as well as online media.

## Discussion

TASCI is the first RCT to investigate the efficacy of TTNS in preventing the development of NDO after acute SCI. It represents a transition in the approach to NLUTD management from treatment of manifested problems to prevention of the disorder itself. The structured follow-up of participants could further inform clinical practice, for example, providing much-needed evidence about the timing of key examinations. Moreover, the inclusion of ‘research-oriented’ secondary outcomes will provide data that may shed some light on the mechanism underlying neuromodulatory therapy, which up until now, is essentially a black box. Additionally, the secondary outcome data could deepen the understanding of the natural trajectory of NLUTD development in the first year after SCI. A final potential benefit of this trial is that it uses a multidisciplinary approach to maximise the chances of identifying and understanding the potential preventative effects of neuromodulatory therapy. The requisite collaboration among experts in neurourology, neuroscience, SCI, rehabilitation medicine, basic and translational research, and biostatistics improves the overall quality of the research and promotes future collaborative endeavours.

The successful development of a therapy that prevents NDO after SCI would represent a huge achievement, especially since there is no known cure, only management of consequences. An effective preventative measure could eliminate lifelong issues associated with SCI, lowering barriers to work force participation and improving QoL, at the same time reducing lifetime healthcare costs. Thus, TASCI represents an important and timely RCT with the potential to revolutionise clinical practice, as well as to provide conceptual insight regarding the mechanism of neuromodulation, and the developmental course of NLUTD following SCI.

### Trial status

This publication is based on version 2 of the TASCI protocol dated 29 October 2019. The official start of recruitment was on 19 June 2019. The estimated end date of the trial is 30 June 2024 and recruitment of patients is ongoing. As of the time of submission, temporary interruptions to recruitment due to the COVID-19 pandemic are a possibility and in case of extended disruption, a schedule adjustment may be required.

## Supplementary Material

Reviewer comments

Author's manuscript
