# Supplementary material for: TASCI—transcutaneous tibial nerve stimulation in patients with acute spinal cord injury to prevent neurogenic detrusor overactivity: protocol for a nationwide, randomised, sham-controlled, double-blind clinical trial
Source: BMJ Open. 2020 Aug 13;10(8):e039164. doi: 10.1136/bmjopen-2020-039164 (PMC7430472; doi:10.1136/bmjopen-2020-039164)
Supplement: Supplementary data [file bmjopen-2020-039164supp001.pdf]

SPIRIT
STANDARD PROTOCOL ITEMS: RECOMMENDATIONS FOR INTERVENTIONAL TRIALS

Supplement 1 – SPIRIT checklist for the TASCI (Transcutaneous Tibial Nerve Stimulation in Acute Spinal Cord Injury) Trial Protocol

SPIRIT 2013 Checklist: Recommended items to address in a clinical trial protocol and related documents*

Section/item	Item No	Description
**Administrative information**		
Title	1	Descriptive title identifying the study design, population, interventions, and, if applicable, trial acronym ✔ **Title, page 1**
Trial registration	2a	Trial identifier and registry name. If not yet registered, name of intended registry ✔ **Abstract, page 4**
	2b	All items from the World Health Organization Trial Registration Data Set – ✔ **Throughout the text, pages 1-21, and publicly accessible information on clinicaltrials.gov, NCT03965299**
Protocol version	3	Date and version identifier ✔ **Trial Status, page 20**
Funding	4	Sources and types of financial, material, and other support - ✔ **Funding Statement, page 21**
Roles and responsibilities	5a	Names, affiliations, and roles of protocol contributors ✔ **Title Page, pages 1-2; Contributor Statement, pages 20-21**
	5b	Name and contact information for the trial sponsor ✔ **Title Page (Corresponding Author), page 2**
	5c	Role of study sponsor and funders, if any, in study design; collection, management, analysis, and interpretation of data; writing of the report; and the decision to submit the report for publication, including whether they will have ultimate authority over any of these activities ✔ **Contributor Statement, Funding Statement , pages 20, 21**
	5d	Composition, roles, and responsibilities of the coordinating centre, steering committee, endpoint adjudication committee, data management team, and other individuals or groups overseeing the trial, if applicable (see Item 21a for data monitoring committee) ✔ **Dissemination, pages 18-19; Contributor Statement, pages 20-21**

Introduction

Background and rationale	6a	Description of research question and justification for undertaking the trial, including summary of relevant studies (published and unpublished) examining benefits and harms for each intervention ✔ **Introduction, pages 5-7**
	6b	Explanation for choice of comparators – ✔ **Introduction, page 6; Methods: Randomisation and blinding, Intervention, page 10**
Objectives	7	Specific objectives or hypotheses ✔ **Introduction, page 6-7**
Trial design	8	Description of trial design including type of trial (eg, parallel group, crossover, factorial, single group), allocation ratio, and framework (eg, superiority, equivalence, noninferiority, exploratory) ✔ **Methods: Study design and setting, page 7; Randomisation and blinding, page 10**

Methods: Participants, interventions, and outcomes

Study setting	9	Description of study settings (eg, community clinic, academic hospital) and list of countries where data will be collected. Reference to where list of study sites can be obtained ✔ **Methods: Study design and setting, page 7**
Eligibility criteria	10	Inclusion and exclusion criteria for participants. If applicable, eligibility criteria for study centres and individuals who will perform the interventions (eg, surgeons, psychotherapists) ✔ **Methods: Participants, page 8; Box 1: Eligibility criteria, page 9**
Interventions	11a	Interventions for each group with sufficient detail to allow replication, including how and when they will be administered ✔ **Methods: Intervention, pages 10-12. Please note: some details have been left vague in order to prevent participants from unblinding themselves with a Google search.**
	11b	Criteria for discontinuing or modifying allocated interventions for a given trial participant (eg, drug dose change in response to harms, participant request, or improving/worsening disease) ✔ **Methods: Intervention, page 12**
	11c	Strategies to improve adherence to intervention protocols, and any procedures for monitoring adherence (eg, drug tablet return, laboratory tests) ✔ **Methods: Intervention, pages 11-12**
	11d	Relevant concomitant care and interventions that are permitted or prohibited during the trial ✔ **Methods: Intervention, pages 11-12, Box 1 Eligibility criteria, page 9**

Birkhäuser V, *et al. BMJ Open* 2020; 10:e039164. doi: 10.1136/bmjopen-2020-039164

Outcomes	12	Primary, secondary, and other outcomes, including the specific measurement variable (eg, systolic blood pressure), analysis metric (eg, change from baseline, final value, time to event), method of aggregation (eg, median, proportion), and time point for each outcome. Explanation of the clinical relevance of chosen efficacy and harm outcomes is strongly recommended ✔ **Methods: Outcomes, pages 12-13; Table 1. Secondary outcomes and outcome measures, page 14; Supplement 3. Secondary outcome parameters**
Participant timeline	13	Time schedule of enrolment, interventions (including any run-ins and washouts), assessments, and visits for participants. A schematic diagram is highly recommended (see Figure) ✔ **Methods: Study design and setting, page 7, Intervention, pages 11-12, Figure 1. Trial Flowchart**
Sample size	14	Estimated number of participants needed to achieve study objectives and how it was determined, including clinical and statistical assumptions supporting any sample size calculations ✔ **Methods: Sample size, page 15**
Recruitment	15	Strategies for achieving adequate participant enrolment to reach target sample size – **Not explicitly mentioned, all patients with a spinal cord injury (SCI) coming through all Swiss specialized SCI centres will be screened for eligibility.**

Methods: Assignment of interventions (for controlled trials)

Allocation:

Sequence generation	16a	Method of generating the allocation sequence (eg, computer-generated random numbers), and list of any factors for stratification. To reduce predictability of a random sequence, details of any planned restriction (eg, blocking) should be provided in a separate document that is unavailable to those who enrol participants or assign interventions ✔ **Methods: Randomisation and blinding, page 10**
Allocation concealment mechanism	16b	Mechanism of implementing the allocation sequence (eg, central telephone; sequentially numbered, opaque, sealed envelopes), describing any steps to conceal the sequence until interventions are assigned ✔ **Methods: Randomisation and blinding, page 10**
Implementation	16c	Who will generate the allocation sequence, who will enrol participants, and who will assign participants to interventions ✔ **Methods: Participants, page 8, Randomisation and blinding, page 10**
Blinding (masking)	17a	Who will be blinded after assignment to interventions (eg, trial participants, care providers, outcome assessors, data analysts), and how ✔ **Methods: Randomisation and blinding, Intervention, pages 10 - 12**

Birkhäuser V, *et al. BMJ Open* 2020; 10:e039164. doi: 10.1136/bmjopen-2020-039164

	17b	If blinded, circumstances under which unblinding is permissible, and procedure for revealing a participant's allocated intervention during the trial – **Not explicitly mentioned, we do not foresee any need to unblind participants for the duration of their participation in the trial, as early evidence and clinical experience has indicated that the intervention is safe.**

Methods: Data collection, management, and analysis

Data collection methods	18a	Plans for assessment and collection of outcome, baseline, and other trial data, including any related processes to promote data quality (eg, duplicate measurements, training of assessors) and a description of study instruments (eg, questionnaires, laboratory tests) along with their reliability and validity, if known. Reference to where data collection forms can be found, if not in the protocol ✔ **Methods: Outcomes, pages 12-13; Table 1 Secondary outcomes, page 14; Supplement 3; Data collection and management, pages 15-16; Statistical Analyses, pages 16-17**
	18b	Plans to promote participant retention and complete follow-up, including list of any outcome data to be collected for participants who discontinue or deviate from intervention protocols ✔ **Methods: Intervention, pages 11-12, Outcomes, page 13**
Data management	19	Plans for data entry, coding, security, and storage, including any related processes to promote data quality (eg, double data entry; range checks for data values). Reference to where details of data management procedures can be found, if not in the protocol ✔ **Methods: Data management, pages 15-16**
Statistical methods	20a	Statistical methods for analysing primary and secondary outcomes. Reference to where other details of the statistical analysis plan can be found, if not in the protocol ✔ **Methods: Statistical analyses, pages 16-17**
	20b	Methods for any additional analyses (eg, subgroup and adjusted analyses) ✔ **Methods: Statistical analyses, pages 16-17**
	20c	Definition of analysis population relating to protocol non-adherence (eg, as randomised analysis), and any statistical methods to handle missing data (eg, multiple imputation) ✔ **Methods: Statistical analyses, pages 16-17**

Birkhäuser V, *et al. BMJ Open* 2020; 10:e039164. doi: 10.1136/bmjopen-2020-039164

Methods: Monitoring

Data monitoring	21a	Composition of data monitoring committee (DMC); summary of its role and reporting structure; statement of whether it is independent from the sponsor and competing interests; and reference to where further details about its charter can be found, if not in the protocol. Alternatively, an explanation of why a DMC is not needed ~ **There is a monitoring committee, but it is responsible for general trial conduct as well as data concerns. Sections: Data management, Quality assurance and oversight, pages 15-18**
	21b	Description of any interim analyses and stopping guidelines, including who will have access to these interim results and make the final decision to terminate the trial ✔ **Methods: Statistical analyses, page 17**
Harms	22	Plans for collecting, assessing, reporting, and managing solicited and spontaneously reported adverse events and other unintended effects of trial interventions or trial conduct ✔ **Methods: Outcomes, page 13; Table 1, page 14; Supplement 3**
Auditing	23	Frequency and procedures for auditing trial conduct, if any, and whether the process will be independent from investigators and the sponsor ✔ **Methods: Quality assurance and oversight, pages 17-18**

Ethics and dissemination

Research ethics approval	24	Plans for seeking research ethics committee/institutional review board (REC/IRB) approval ✔ **Ethics and Dissemination: Ethics, page 18**
Protocol amendments	25	Plans for communicating important protocol modifications (eg, changes to eligibility criteria, outcomes, analyses) to relevant parties (eg, investigators, REC/IRBs, trial participants, trial registries, journals, regulators) – **Not directly stated: by default in Switzerland all important protocol modifications have to be communicated to the ethics committees, who will pass information along regulators as appropriate. Trial participants receive an update letter, and there are representatives from each investigating centre on the steering committee.**
Consent or assent	26a	Who will obtain informed consent or assent from potential trial participants or authorised surrogates, and how (see Item 32) ✔ **Methods: Participants, page 8**
	26b	Additional consent provisions for collection and use of participant data and biological specimens in ancillary studies, if applicable ✔ **Methods: Outcomes, page 13**

Confidentiality	27	How personal information about potential and enrolled participants will be collected, shared, and maintained in order to protect confidentiality before, during, and after the trial ✔ **Methods: Data management, pages 15-16; Ethics and dissemination, pages 18-19**
Declaration of interests	28	Financial and other competing interests for principal investigators for the overall trial and each study site ✔ **Competing interests, page 21**
Access to data	29	Statement of who will have access to the final trial dataset, and disclosure of contractual agreements that limit such access for investigators ✔ **Contributor Statement, Dissemination, pages 18-19**
Ancillary and post-trial care	30	Provisions, if any, for ancillary and post-trial care, and for compensation to those who suffer harm from trial participation - ✔ **Methods: Intervention (ancillary care), pages 11-12. The need to compensate for harm is not foreseen, previous studies and clinical experience in other patient groups have not raised any safety concerns**
Dissemination policy	31a	Plans for investigators and sponsor to communicate trial results to participants, healthcare professionals, the public, and other relevant groups (eg, via pub lication, reporting in results databases, or other data sharing arrangements), including any publication restrictions ✔ **Ethics and Dissemination: Patient and public involvement, Dissemination, pages 18-19**
	31b	Authorship eligibility guidelines and any intended use of professional writers ✔ **Ethics and Dissemination: Dissemination, pages 18-19**
	31c	Plans, if any, for granting public access to the full protocol, participant-level dataset, and statistical code ✔ **Ethics and Dissemination: Dissemination, pages 18-19**
**Appendices**		
Informed consent materials	32	Model consent form and other related documentation given to participants and authorised surrogates ✔ **Supplement 2**
Biological specimens	33	Plans for collection, laboratory evaluation, and storage of biological specimens for genetic or molecular analysis in the current trial and for future use in ancillary studies, if applicable ✔ **Methods: Outcomes, pages 12-13; Figure 1; Table 1, page 14; Supplement 2, Supplement 3**

*It is strongly recommended that this checklist be read in conjunction with the SPIRIT 2013 Explanation & Elaboration for important clarification on the items. Amendments to the protocol should be tracked and dated. The SPIRIT checklist is copyrighted by the SPIRIT Group under the Creative Commons "Attribution-NonCommercial-NoDerivs 3.0 Unported" license.

Birkhäuser V, *et al. BMJ Open* 2020; 10:e039164. doi: 10.1136/bmjopen-2020-039164
