# Supplementary material for: TASCI—transcutaneous tibial nerve stimulation in patients with acute spinal cord injury to prevent neurogenic detrusor overactivity: protocol for a nationwide, randomised, sham-controlled, double-blind clinical trial
Source: BMJ Open. 2020 Aug 13;10(8):e039164. doi: 10.1136/bmjopen-2020-039164 (PMC7430472; doi:10.1136/bmjopen-2020-039164)
Supplement: Supplementary data [file bmjopen-2020-039164supp002.pdf]

Study information and declaration of consent

Transcutaneous tibial nerve stimulation in patients with acute spinal cord injury to prevent neurogenic bladder dysfunction

Official study acronym: **TASCI**
(Transcutaneous **T**ibial Nerve Stimulation in **A**cute **S**pinal **C**ord **I**njury)

This study is organised by: Balgrist University Hospital

Dear Sir or Madam

We would like to ask you to participate in a nationwide clinical study. The study investigates the effect of early phase electrical stimulation of the tibial nerve (transcutaneous tibial nerve stimulation (TTNS)) on bladder dysfunction after acute spinal cord injury. Below you will find a detailed description of the study. Please read the study information carefully before you decide whether or not you will participate.

Summary

1	**Study aim**<br>We would like to ask you to participate in our clinical trial. The study investigates whether early phase electrical stimulation of the tibial nerve can prevent the development of harmful bladder dysfunction.
2	**Selection for participation**<br>You have recently suffered a spinal cord injury at the level of the cervical or thoracic vertebrae. As bladder condition often deteriorates over the course of the first few weeks to months, you may benefit from early phase TTNS treatment. That is why we are giving you this study information.
3	**General information about the study**<br>Spinal cord injuries usually lead to bladder dysfunction, which often worsens over time and is associated with a risk for kidney failure and reduced quality of life. In this Switzerland-wide study we investigate the effect of early phase TTNS on bladder dysfunction after acute spinal cord injury. A total of 114 patients at the four specialized spinal cord injury centers in Switzerland, in Basel, Nottwil, Sion and Zurich, will be included in the study. Patients are randomly assigned to two groups: 57 patients will be treated with TTNS (verum), 57 patients will receive a sham treatment (sham). Neither patients nor the doctors treating them know which group the patients are assigned to, it is a double-blind study. The treatment is administered five times a week for 30 minutes, during a period of 6-9 weeks. The stimulation device (transcutaneous electrical nerve stimulation (TENS)) used for the treatment (ELPHA II 3000, medical device, CE 0543 Certification, FH Service) is CE certified and approved in Switzerland. The stimulation parameters correspond to the standards currently used in routine clinical practice for the treatment of chronic bladder dysfunction. The duration of the study for each individual participant is 1 year, with preliminary and follow-up examinations taking place at 4 fixed time points. The total duration of the study is 4 years.

Birkhäuser V, et al. BMJ Open 2020; 10:e039164. doi: 10.1136/bmjopen-2020-039164

4	**Procedure**<br>For you, the study will last for a total of 1 year, and for much of that time you will receive inpatient care at our clinic. During your hospital stay, you will undergo some baseline examinations for the study which will take place within the framework of your routine clinical examinations. This includes examinations of bladder pressure and bowel function, neurophysiological assessments, as well as magnetic resonance imaging for all suitable study participants. Furthermore, you will fill in a set of questionnaires about your condition (that is, your bladder and bowel function) and keep a bladder diary and a bowel diary for three or seven days, respectively.<br>This is followed by the 6-9 week treatment phase with five treatment sessions per week. Treatment groups are randomly assigned and neither you nor your study doctor knows which group you have been assigned to. Three, 6 and 12 months after spinal cord injury, follow-up examinations are performed, and they include the same tests as the baseline examinations. At the end of the study, you will learn which group you were assigned to. The baseline and follow-up examinations include assessments of your physical, neurological and urological condition, as well as analyses of blood, urine, and stool, and possibly also bladder tissue samples (at the end of the baseline examinations and 6 months after spinal cord injury).
5	**Benefit**<br>TTNS may have a positive effect on your bladder and sexual function. Through your participation we are gaining important information about the effectiveness of TTNS in preventing bladder problems.
6	**Rights**<br>You can freely decide whether or not you want to participate in the study. Your decision does not affect your medical care and you do not have to justify your decision.
7	**Responsibilities**<br>If you participate, we ask you to adhere to certain requirements. No other electrical stimulation of nerves or muscles (with the exception of the upper extremities) for therapeutic purposes may be performed on you for the entire duration of the study (12 months). You should inform your study doctor or study team of all treatments and medications you are currently receiving. Please talk to us as early as possible before any treatment changes are made, whether it is in the form of a change in dosage or the addition of a new treatment.
8	**Risks**<br>Overall, TTNS has an excellent safety profile, that is, it has almost no side effects. In very rare cases, skin reactions can occur in the area of where the surface electrodes are placed.<br>When measuring bladder pressure (urodynamic investigation) and stimulating the lower urinary tract, the insertion of the measurement catheters into the bladder and anus can be uncomfortable and can be associated with a slight burning sensation. Very rarely, the insertion of the catheter can lead to inflammation of the bladder, or injury to the urethra that could cause the urethra to narrow (urethral strictures).<br>The bowel assessments (anorectal manometry) are clinically established and are regarded as safe examinations. The insertion of the catheter can be perceived as unpleasant. A painful sensation indicates a medical problem, which can then be directly examined and, if necessary, treated.<br><u>When tissue samples are taken from the bladder (only applicable if you specifically agree to participate in this part of the study)</u>, a bladder infection or bleeding from the bladder may occur, which, very rarely, requires an additional procedure (cystoscopy) to stop the

	bleeding (haemostasis). In very rare cases, the intervention might cause a perforation (small hole) in the bladder. If this occurs, a catheter has to remain in the bladder for an extended period of time to allow the bladder to heal.
9	**Other treatment options**<br>Your doctor will discuss your other available treatment options with you.
10	**Results**<br>You will be informed of study results that are important for your health. If you do not want to receive this information, please inform your study doctor.
11	**Confidentiality of data and samples**<br>We adhere to all legal regulations regarding data protection and all parties involved are subject to the obligation of professional confidentiality. Your personal and medical data and your biological material / samples (blood, urine, etc.) are protected, and used in an encrypted form. If applicable: the data and samples you provide will only be used for other research projects if you give your separate consent.
12	**Withdrawal**<br>You can withdraw from the study at any time if you no longer wish to participate. The data and samples collected up to the time of your withdrawal will still be evaluated.
13	**Compensation**<br>There is no compensation for participating in this study.
14	**Liability**<br>The University of Zürich has taken out an insurance policy with Zürich Versicherungs-Gesellschaft AG (police: 14.970.888) for the research department of the Spinal Cord Injury Centre at Balgrist University Hospital (Forchstrasse 340, 8008 Zürich) to cover liability in the event of a possible claim.
15	**Funding**<br>The Swiss National Science Foundation (SNSF) is the primary source of funding for this study.
16	**Contact information**<br>If any questions, uncertainties or emergencies arise during or after the study, you can always contact the following persons:

Study Doctor (Principal Investigator):	Deputy:
Prof.Thomas M. Kessler, MD<br>Neuro-Urology / Spinal Cord Injury Centre<br>Balgrist University Hospital<br>Forchstrasse 340<br>8008 Zürich<br>phone +41 (0)44 386 39 07 (weekdays during working hours)	Dr. Ulrich Mehnert, MD, PhD<br>Neuro-Urology / Spinal Cord Injury Centre<br>Balgrist University Hospital<br>Forchstrasse 340<br>8008 Zürich<br>phone +41 (0)44 386 39 07 (weekdays during working hours)
+41 (0)44 386 11 11 (in case of emergency, outside of regular working hours)	

Detailed information

1. Study aim

Spinal cord injuries usually lead to bladder dysfunction, which can endanger kidney function and reduce quality of life. This study investigates whether early phase electrical stimulation of the tibial nerve (transcutaneous tibial nerve stimulation, TTNS) can prevent the development of harmful bladder dysfunction. To better understand the mechanism of this treatment, comprehensive neuro-urological, imaging, and laboratory, including histological (tissue sample), assessments will be performed.

2. Selection for participation

All adults who are affected by an acute spinal cord injury (up to 40 days after injury) at the cervical or thoracic vertebral level can participate. However, persons with contraindications to the use of electrical stimulation are excluded from this study.

3. General information

Bladder dysfunction is one of the most important problems of patients with spinal cord injury, and if it is left untreated, it can significantly reduce quality of life and cause damage to the upper urinary tract that can even lead to kidney failure. Current treatments are often insufficient or associated with significant side effects. We urgently need new treatments that can prevent bladder dysfunction before permanent damage occurs. TTNS is already successfully being used to treat existing bladder dysfunction.

In a nationwide study in which all specialized spinal cord injury centres in Switzerland (Basel, Nottwil, Sion and Zürich) are participating, we are investigating the preventive effect of early phase TTNS on bladder dysfunction resulting from acute spinal cord injury. A total of 114 patients will be included in the study over a period of four years and randomly assigned to two groups: 57 patients will be treated with TTNS (verum), 57 patients will receive a sham treatment (sham). Neither the patients nor the doctors treating them know which group the patient is assigned to, it is a double-blind study. The treatment is administered for 30 minutes, 5 times a week, for 6-9 weeks using adhesive electrodes placed on the foot. The stimulation device (transcutaneous electrical nerve stimulation (TENS)) used for the treatment (ELPHA II 3000, CE 0543 Certification, FH Service) is CE certified and approved in Switzerland. The current settings correspond to the standards used in routine clinical practice for the treatment of neurogenic bladder dysfunction.

The electrical impulses are conducted up the leg via the tibial nerve to the spinal cord (which plays an important role in controlling the bladder) and, if used repeatedly, leads to an improvement in bladder condition in many patients. It is thought that electrical impulses transmitted from the tibial nerve via the spinal cord to the brain have a positive effect on the bladder centres in the central nervous system, although the exact mechanisms of action are not yet fully understood. The repeated application of this electrical stimulation seems to be particularly important for the success of the treatment. Experience shows that noticeable improvement only occurs after several stimulation sessions. This type of therapy has proven to be effective in patients with chronic bladder problems and offers an excellent and almost side-effect free alternative to treatments such as Botox injections into the bladder muscle or operations on the bladder.
The duration of the study is 1 year for each study participant, with baseline and follow-up examinations taking place at 4 fixed time points.
In order to better understand the developments that occur after spinal cord injury and the mechanism of action of TTNS, comprehensive neuro-urological, imaging, and laboratory assessments (including histological analysis of tissue samples) will be carried out during the course of this study. Your bladder dysfunction will be assessed using questionnaires and the data from routine clinical examinations (bladder function examination, neuro-urological examination, neurophysiological examination, and analyses of blood, urine and stool samples) in combination with data from study-specific assessments. In addition, we are performing cellular-level investigations (using blood, urine, stool and bladder tissue samples) to identify important

mechanisms which for example, may control cell division, cell proliferation and cell death. Furthermore, you will undergo routine bowel function examinations, in particular regarding sensation in the area of the anus and rectum, as well as rectal pressure conditions and muscle strength. It is possible that findings from these examinations can also be used for diagnostic or treatment purposes at a later point in time.

The study will be conducted in accordance with Swiss regulations, and with all internationally recognised guidelines. The responsible cantonal ethics committee has evaluated and approved the study. A description of this study can also be found on the website of the Swiss Federal Office of Public Health: www.kofam.ch.

4. Study procedure

If you are eligible for this study according to our inclusion and exclusion criteria, a member of the study team will a member of the study team will discuss the study with you in detail (informed consent discussion). You will have time to ask questions and you will receive this study information to read and consent forms to sign. Once you are enrolled in this study, in addition to the TTNS treatment, you will undergo baseline and follow-up examinations that occur at fixed time points over a period of 1 year (see chart below). The individual assessments that make up these examinations can be spread over several days. The examinations can vary in terms of time and according to your state of health, but should not take more than half a day in the longest case.

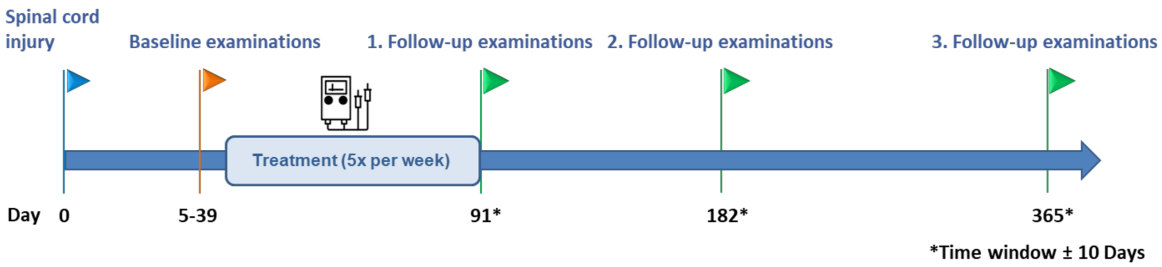

The **baseline examinations** take place up to 40 days after your spinal cord injury.

First, your medical history is taken, a list of your medications is collected, and you undergo a comprehensive medical examination:

- Physical examination
- Neurological examination:
  - Assessment of the severity of your paralysis: this includes an examination of your muscle strength, mobility, sensation (sensitivity to light touch and ability to distinguish between sharp and blunt objects) as well as sensation and conscious control of the anus
  - Reflexes
  - Muscle stiffness (spasticity)
  - Functional status (autonomy, breathing, management of bladder and bowel dysfunction, mobility)
  - Various walking tests for patients capable of walking, for example to record the walking aids used
- Assessment of health state, particularly bladder and bowel function using questionnaires:
  - Bladder emptying and sensation as well as catheter use
  - Your personal experiences, limitations and perceptions associated with your bladder dysfunction.
  - Bladder diary (over a period of 3 days): Documentation of times and amounts of fluid intake and urine excretion as well as incontinence episodes and use of adsorbent pads. We will provide you with a bladder diary and a urine measuring cup.
  - Bladder function (International Prostate Symptom Score, IPSS)
  - Urinary tract symptoms (Urinary Symptom Profile, USP)

Birkhäuser V, et al. BMJ Open 2020; 10:e039164. doi: 10.1136/bmjopen-2020-039164

- Quality of life (Qualiveen)
- Bowel function (Neurogenic Bowel Dysfunction Questionnaire, NBD)
- Sexual function (Female Sexual Function Index, FSFI; International Index of Erectile Function, IIEF)
- Spasticity after spinal cord injury (Spinal Cord Injury Spasticity Evaluation Tool, SCI-SET)

- Measurement of muscle function, nerve conduction, including nerve conduction velocity, as well as electrical activity in the brain. During these examinations, your nerves will be stimulated using short, light electrical impulses, among other things. For this purpose, adhesive electrodes (like those used for an electrocardiogram, (ECG)) or needle electrodes are placed at different positions on your body, for example, on your hand, or under the skin of your head, and a short electrical stimulus is triggered. This can be perceived as a tingling sensation. Sometimes this test is perceived as unpleasant. These sensations disappear when the test is finished and leave no side effects. This examination can last up to an hour.
- Bowel examinations
  - Anorectal manometry: in this clinical examination the pressure of the anal sphincter muscle, the sensation in the rectum and the neuronal reflexes are tested. This serves to assess the function of the anal sphincter. If abnormalities are found during the examination (for example, faecal incontinence, constipation or defecation disorders), the examination serves as a basis for further action.
  - Examination of the rectum and anus (proctoscopy): with this examination, diseases in the area of the anus, anal canal and lower rectum can be detected and, in some cases, treated directly.

Magnetic resonance imaging (MRI) is routinely used for capturing images of organs and tissues in a non-invasive and harmless manner. However, this examination cannot be performed if you have certain types of metal implants or a pacemaker, which you will be asked about separately. For the MRI examination, you must leave all metallic objects in a changing room. Before the examination, the scanning procedure will be explained to you one more time. You will then be accompanied to the MRI room where you will lie down on the examination table.
In order to make it as comfortable as possible for you to lie still, and to prevent unwanted movements, we use foam wedges, padding and velcro straps. You will be given headphones that you can use to communicate with the examiners at any time. You are positioned in the MRI machine and while you are lying very still on your back, images are taken of the spine and brain. In addition, brain function is measured at rest (resting activity, rMRI) and during electrical stimulation of the tibial nerve (functional MRI, fMRI). By repeating the assessment at several time points, we can detect changes that occur during the one-year course of the study. You will be able to listen to music during the some of the assessments. The MRI examination is radiation-free and it does not produce side effects. The headphones reduce the unpleasant noise caused by the machine. No contrast medium is required for the above-mentioned examinations, so no injections are administered.

As part of the normal hospital routine, **blood** and **urine samples** are collected from you, and slightly larger quantities will be taken for study purposes. In addition, stool samples will be collected, and, if you give your separate consent, tissue samples will also be collected from the bladder. The tissue samples are taken from the bladder under general or spinal (regional) anaesthesia using an optical instrument (cystoscope) with biopsy forceps / electrical loop inserted into the bladder through the urethra.

After completion of the baseline examinations, participants will be randomly divided into two treatment groups (actual TTNS (verum) or sham TTNS (sham)). You will be assigned to a treatment group using a random procedure, comparable to the tossing a coin (randomisation). The random assignment ensures that the groups are comparable. You cannot influence the treatment group assignment nor can your study doctor or any other person.

The treatment will be carried out by specially trained study personnel 5 times a week for 6-9 weeks until the first follow-up assessments. For the treatment, 4 adhesive electrodes are placed on the foot and during a preparatory phase, various current intensities and other parameters are tested to optimize the nerve stimulation conditions. Afterwards, the treatment, according to your group assignment, can begin. In order to ensure that the study data is credible and cannot be influenced, neither you nor your study doctor can know whether you are receiving verum or sham TTNS (this procedure is called "double-blind"). Every week you will fill in questionnaires to assess bladder awareness (University of South Australia Urinary Symptom Assessment (USA2)), bladder function (International Prostate Symptom Score (IPSS)) & Urinary Symptoms Profile (USP), spasticity (Spinal Cord Injury Spasticity Evaluation Tool (SCI-SET)), and quality of life (Qualiveen) as well as bowel function (Neurogenic Bowel Dysfunction Questionnaire (NBD)).

During the follow-up examinations, you will undergo repeats of the same clinical and research examinations that were performed at the study start. This means undergoing routine clinical examinations, filling in a bladder and bowel diary as well as all of the questionnaires mentioned above. Urine and blood samples are also collected again. In addition, bladder pressure, bowel function, and neurophysiological measurements are carried out, as well as an MRI examination. These examinations will occur 3, 6 and 12 months after spinal cord injury. During the second follow-up examination (6 months after spinal cord injury) if you consent to the removal of bladder tissue another sample will be taken. Your participation in the study will be complete after the 3rd follow-up examination (12 months after spinal cord injury).

We may have to prematurely end your participation in the study, if, for example, you have health problems that cannot be treated, or cannot be sufficiently treated, with electrotherapy or if the study protocol is not followed. In this case, you will undergo one final examination for your own safety. Your general practitioner will be informed about your participation in this study.

5. Benefits

If you participate in this study, early phase TTNS might prevent serious bladder dysfunction that is likely to occur due to your spinal cord injury. This could reduce the necessity of using long-term and often ineffective treatments, improve quality of life and reduce healthcare costs. This would be a milestone in the treatment of patients with spinal cord injury in the sense of "prevention is better than treatment". If you receive sham treatment, the course of the disease is unlikely to be affected. However, you will benefit from detailed examinations, which enhance monitoring of rehabilitation progress and allow for the immediate identification and treatment of health problems.

6. Rights

You participate in this study on a voluntary basis. If you do not wish to participate or later withdraw your consent, you do not need to justify your decision. Your medical care is guaranteed and independent of your decision to participate in the study. You may ask questions about the study at any time. The contact person, whose details are supplied at the end of this document, will be happy to answer your questions.

7. Responsibilities

As a participant, over the entire study year, it is necessary that you:

▪ adhere to the instructions and requirements outlined in the study plan (study protocol).

▪ inform your study doctor about your disease course and report any new symptoms, problems, or changes in your health condition (also after the end of the treatment phase or at the time of discontinuation of the study, for example, until any side effects disappear).

▪ always contact a member of the study team first to discuss any changes to your treatment plan (for example, changing the doses of your existing medications, adding a new medication or treatment, including complementary medicine and/or other treatments such as electrical muscle stimulation or similar treatments).

▪ bring a fully completed 3-day bladder diary and questionnaires with you to all baseline and follow-up examinations.

▪ do not receive any other electrical stimulation of nerves or muscles for therapeutic purposes (exception: functional electrical stimulation (FES) of the upper extremities), as it is sometimes used as a therapeutic approach during spinal cord injury rehabilitation. After the end of the study, these treatments can be used without restriction.

8. Risks and burdens for the participants

Overall TTNS has an excellent safety profile, meaning it has almost no side effects. In very rare cases, skin reactions in the area of the adhesive electrodes and/or an unpleasant tingling sensation in the feet with slight numbness may occur for a short time after the TTNS session. To date, severe, life-threatening or permanent damage due to TTNS have not been reported in any study.

For measuring bladder pressure (urodynamic investigation, a routine clinical procedure) and stimulating the lower urinary tract, bladder filling using an inserted catheter is necessary. Bladder filling can cause a feeling of tension and an unpleasant urge to urinate. In rare cases, the catheter insertion can cause a urinary tract infection, which would manifest as pain and a burning sensation when urinating. Very rarely, the urethra could be injured resulting in urethral narrowing (urethral stricture). If such symptoms occur, please inform the contact persons listed below immediately.

The only examination that is a source of radiation exposure is the video-urodynamic investigation, but this is a routine clinical examination which has a direct benefit for the patient. Patients with an acute spinal cord injury have a high risk of developing serious bladder problems, which in the worst case could lead to kidney failure. Therefore, all patients will undergo regular video-urodynamic investigations as part of their routine neuro-urological follow-ups.

Magnetic resonance imaging (MRI) has been an integral part of medical diagnostics for a long time. Images of the inside of the body are generated using radio frequency waves in a strong magnetic field. Magnetic resonance imaging is not known to cause harmful side effects. In particular, there is no radiation exposure. In order to avoid any risk, metallic or magnetic objects must be removed and stored before the examination.

Bowel examinations (anorectal manometry) are clinically established and are regarded as safe examinations. The insertion of the catheter can be perceived as unpleasant. Pain indicates a medical problem, which can then be directly examined and treated if necessary. In rare cases, the movement of the probe in the rectum and the inflation of the balloon can cause superficial injuries to the intestinal mucosa.

During **blood collection**, bruising may occur in the area of the puncture site. There is a very low risk of local or general infection. In extremely rare cases, injury to a nerve in the skin may occur, and possibly even progress to a chronic problem.

In rare cases, **taking tissue samples from the bladder** can cause a bladder infection or bleeding, which can very rarely result in a blood transfusion or a further intervention (cystoscopy) to control the bleeding (hemostasis). In extremely rare cases, a bladder perforation (small hole in the bladder) might occur during the intervention. In this case, a catheter has to remain in the bladder for an extended period of time to allow the bladder to heal. As with any procedure, general complications such as thromboses and embolisms can occur.

All other examinations and test procedures are non-invasive or minimally invasive.

For women who can become pregnant

For women of childbearing age (by definition not menopausal, last menstrual period within the last 12 months, no tubal ligation, ovaries and/or uterus not surgically removed) a free pregnancy test is carried out before the first examination. If this test is positive, you cannot participate in the study for your own safety. If you become pregnant during the study, you must inform your study doctor immediately, and may no longer participate in the study for your safety. In this case this does happen, you will be asked to provide information about the course and outcome of the pregnancy. The study doctor will discuss the procedures with you. If you are breastfeeding, you cannot participate in the study.

Birkhäuser V, *et al. BMJ Open* 2020; 10:e039164. doi: 10.1136/bmjopen-2020-039164

9. Other treatment options

There are currently no clinical treatment options to prevent the development of serious bladder dysfunction. If you do not wish to participate or wish to withdraw from the clinical trial at a later date, this will not affect your medical care.

10. Results of the study

The study doctor will inform you of any new knowledge acquired during the study that could affect your evaluation of potential benefits from the study or your safety and thus your consent to participate in the study. You will receive this information orally as well as in writing.
In principle, you will be informed about incidental findings (for example in MRI or genetic analyses) that may have implications for your present or future state of health, including the prevention, detection and treatment of existing or potential diseases. If you do not wish to be informed (so-called "right not to know"), please discuss this with your study doctor and ensure that you check the relevant box on your declaration of consent form.

11. Confidentiality of data and samples

For this study, your personal and medical data will be collected. Very few professionals will see your unencrypted data, and only to perform tasks within the study. When data is collected for study purposes, the data is encrypted. Encryption means that all reference data that could identify you (name, date of birth) is replaced by a code. The list of codes always remains in the study centres. Therefore, those persons who do not know the code cannot identify you. In scientific studies, the data is summarized and it is not possible to draw conclusions about you as an individual. Your name will never appear on the internet or in a publication. Sometimes scientific journals require that individual data (so-called "raw data") is submitted. If individual data have to be submitted, the data is always encrypted and therefore not traceable to you as a person. All persons who have access to your data within the scope of the study are subject to confidentiality obligations. All data protection regulations are observed, and you as a participating person have the right to view your data at any time.

If data / samples are stored on site, it is in a database / biobank for research purposes. These data and samples can be sent in encrypted form to another database / biobank within the framework of this study. The biological samples that were collected specifically for this study are usually no longer available for diagnostic purposes.

The data and biological samples will be shared in encrypted form for analysis purposes among the four participating study centres, and also sent to Bern (Switzerland), Leipzig (Max Planck Institute, Germany), London (United Kingdom), Porto (Portugal) and Pittsburgh (USA) for special analyses, where they will be examined for this project and then stored for 10 years. The code list will remain in the recruiting study centre and only the local study team will have access to it. The sponsor is responsible for ensuring that the international sites maintain the same standards as those in Switzerland.

It is possible that your data and samples may be used for other investigations at a later date or may be sent to another database / biobank in Switzerland or abroad for use in additional analyses that are not yet specified (further use). These other databases / biobanks must comply with the same standards as the database / biobank currently used in this study. To allow this further use, we ask you to sign the additional declaration of consent at the very end of this document.

It is possible that this study will be reviewed by the competent ethics committee, Swissmedic, or by the sponsor (or their representative responsible for quality assurance). The study team may need to disclose your personal and medical data for such reviews. It is possible that your treating physician may be contacted to provide information about your state of health. In the course of this project, data will be collected which may also be of interest to your attending doctor and thereby relevant for your personal medical care. Therefore, this data (for example: data from neurological examinations, bladder function, function of muscles and nerves, and magnetic resonance images)

may also be stored in your electronic medical file. This primarily serves to reduce duplicate examinations and thus to reduce your examination burden.

12. Withdrawal
You can withdraw from the study at any time if you no longer wish to participate. The data and samples collected up until that point will still be analysed (in encrypted form), so as not to lower the value of the project. It is not possible to anonymize your data and samples when you withdraw, meaning the data and samples remain encrypted. Please make sure that you agree to this before you participate in this study.

13. Compensation
You will not receive any compensation for participating in this study.

14. Liability
Balgrist University Hospital, which initiated the study and is responsible for conducting it, is liable for any damage you may suffer in relation to the medical product tested or to research activities (for example, the examinations). The requirements and procedure for this are regulated by law. The University of Zürich has an insurance policy with Zürich Versicherungs-Gesellschaft AG (police: 14.970.888) for the department of research at the Spinal Cord Injury Centre at Balgrist University Hospital (Forchstrasse 340, 8008 Zürich) to cover liability in the event of a claim.
In the event of damage that is attributable to an approved therapy administered in accordance with existing medical standards (certified medical device used according to the instructions) or that also would have occurred if a standard treatment had been used, the same liability rules apply as for treatments occurring outside of a research framework. If you have suffered damage, please contact your study doctor or the insurance company mentioned above. The necessary steps will then be taken for you.

15. Funding of the study
The Swiss National Science Foundation (SNSF) is the primary source of funding for this study.

16. Contact information
If any questions, uncertainties or emergencies arise during or after the study, you can always contact the following persons:

Study Doctor (Principal Investigator):	Deputy:
Prof. Thomas M. Kessler, MD<br>Neuro-Urology / Spinal Cord Injury Centre<br>Balgrist University Hospital<br>Forchstrasse 340<br>8008 Zürich<br>phone +41 (0)44 386 39 07 (weekdays during working hours)	Dr. Ulrich Mehnert, MD, PhD<br>Neuro-Urology / Spinal Cord Injury Centre<br>Balgrist University Hospital<br>Forchstrasse 340<br>8008 Zürich<br>phone +41 (0)44 386 39 07 (weekdays during working hours)
Phone: +41 (0)44 386 11 11 (in case of emergency, outside of regular working hours)	

Birkhäuser V, *et al. BMJ Open* 2020; 10:e039164. doi: 10.1136/bmjopen-2020-039164

Declaration of consent to participate in the TASCI study

Please read this form carefully. Please ask if you do not understand something or would like to know something. Your written consent is required for participation in this study.

**Title of the study**:<br>Transcutaneous tibial nerve stimulation in patients with acute spinal cord injury to prevent neurogenic bladder dysfunction<br>(Full title: Transcutaneous tibial nerve stimulation in patients with acute spinal cord injury to prevent neurogenic detrusor overactivity: A nationwide randomised, sham-controlled, double-blind clinical trial)	
**Acronym/Short title:** TASCI	**BASEC-number: 2019-00074**
**Responsible institute (Sponsor with address**)	University of Zürich<br>c/o Balgrist University Hospital<br>Forchstrasse 340<br>8008 Zürich
**Study site**	Neuro-Urology / Spinal Cord Injury Centre<br>Balgrist University Hospital
**Responsible clinical investigator at the study site**<br>First name and last name (in block letters):	
**Participant**<br>First name and last name (in block letters):	
Gender: ☐ female ☐ male	Date of birth: __ __. __ __. __ __ __ __
Participant ID: ____________________	Screening-ID (SWISCI-ID): ________________

- I have been informed verbally and in writing by the undersigned clinical investigator about the purpose and procedure of this study using the stimulation device (ELPHA II 3000) and about possible advantages and disadvantages as well as possible risks.
- I am participating in this study on a voluntary basis and accept the contents of the written study information that has been provided to me. I have had sufficient time to make my decision.
- My questions regarding participation in this study have been answered. I will keep the written information and receive a copy of my consent form.
- I agree that my general practitioner will be informed about my participation in the study.
- I agree that the responsible expert representatives of the sponsor and the competent ethics committee may inspect my unencrypted data for examination and quality control purposes, but in strict compliance with confidentiality requirements.
- In the event that study results or incidental findings have direct implications for my present or future health, I will be informed unless I explicitly state otherwise. If I do not wish to be informed, I hereby notify my study doctor (clinical investigator) and confirm that I do not want to receive this type of information by checking the box below:

  ☐ I do NOT wish to be informed about results or incidental findings

Supplemental material BMJ Publishing Group Limited (BMJ) disclaims all liability and responsibility arising from any reliance placed on this supplemental material which has been supplied by the author(s) BMJ Open

- I am aware that my health-related and personal data (e.g. MRI and samples) can only be transferred in encrypted form (pseudonymised) for research purposes for this study (also abroad).
- In the event of further treatment outside the trial site, I authorize my general practitioner and attending doctors to transmit my post-treatment data relevant to the trial to the clinical investigator.
- I can withdraw from the study at any time and without justification. My continuing medical treatment is always guaranteed regardless of my participation in the study. Any data and samples collected up to the time of my withdrawal will be evaluated in the study.
- The liability insurance of the hospital/institution is responsible for covering any damages.
- I am aware that the requirements mentioned in the participant information must be fulfilled. In the interest of my health, the investigator may exclude me from the study at any time.
- I have also been informed about the accompanying research on molecular biological changes occurring in persons with bladder dysfunction.

☐ I agree,

☐ I do NOT agree,

that tissue samples can be collected from my bladder during the baseline examination and 6-month follow-up examination.

Place:<br><br>Date [dd.mm.yyyy]:<br><br>__ __. __ __. __ __ __ __	Signature of the participant

Confirmation by the investigator: I hereby confirm that I have explained the nature, significance and scope of the study to this participant. I confirm that I will fulfil all obligations in relation to this study in accordance with the applicable laws. If at any time during the course of the study I learn of anything that could influence the participant's willingness to participate in the study, I will inform him/her immediately.

Place:<br><br>Date [dd.mm.yyyy]:<br><br>__ __. __ __. __ __ __ __	Last name and first name of the investigator (in block letters):<br><br>Signature of the investigator

Birkhäuser V, *et al. BMJ Open* 2020; 10:e039164. doi: 10.1136/bmjopen-2020-039164

Consent to further use of data and biological material from this study for biomedical research

**Participant**<br>First name and last name (in block letters):	
Gender: ☐ female ☐ male	Date of birth: __ __. __ __. __ __ __ __
Participant ID:	Screening-ID. (SWISCI-ID):

I allow my data and samples from this study to be used for medical research purposes. This means that the health-related data (such as MRI images) and biological materials may be stored in a database or biobank and used for future, as of yet undefined, research projects for an indefinite period of time. This consent is valid for an unlimited period of time.

I am making this decision on a voluntary basis and can revoke this decision at any time. If I withdraw my consent, my data will be made anonymous and my samples will be destroyed. I only need to inform my study doctor (clinical investigator), and do not have to justify this decision.

I understand that the data and samples are encrypted and that the code list is stored securely. The data and samples can be sent to other databases and biobanks in Switzerland as well as abroad for analysis, provided that they maintain the same standards as in Switzerland. All legal requirements regarding data protection are fulfilled.

Usually, all data and samples are evaluated on a group level and the results are published in summary form. If a result that is important for my health should arise, it is possible that I will be contacted via my study doctor. If I do not wish to be contacted, I will inform my study doctor.

If results from the data and samples are commercialized, I have no claim to a share of the commercial use.

Place:<br><br>Date [dd.mm.yyyy]:<br><br>__ __. __ __. __ __ __ __	Signature of the participant:

Confirmation by the investigator: I hereby confirm that I have informed the participant of the nature, significance and scope of the further use of biological samples and/or genetic data.

Place:<br><br>Date [dd.mm.yyyy]:<br><br>__ __. __ __. __ __ __ __	Last name and first name of the investigator (in block letters):<br><br>Signature of the investigator:
