# Supplementary material for: TASCI—transcutaneous tibial nerve stimulation in patients with acute spinal cord injury to prevent neurogenic detrusor overactivity: protocol for a nationwide, randomised, sham-controlled, double-blind clinical trial
Source: BMJ Open. 2020 Aug 13;10(8):e039164. doi: 10.1136/bmjopen-2020-039164 (PMC7430472; doi:10.1136/bmjopen-2020-039164)
Supplement: Supplementary data [file bmjopen-2020-039164supp003.pdf]

Supplement 3. Secondary outcome parameters. Assessment periods are referenced to the date of spinal cord injury (SCI) diagnosis and include: screening and baseline (S&B; days 5-39), 3-month follow-up (3M; day 91 +/-10), 6-month follow-up (6M; day 182 +/-10), 12-month follow-up (12M; day 365 +/-10). Additionally, some surveys are administered weekly during the TTNS treatment period (TTNS: weekly), and the bowel and bladder diaries are administered once every two weeks during the treatment period (TTNS: bi-weekly).

Outcome and Measurement Parameter (analysis metric)	Assessment Periods
**Occurrence of neurogenic detrusor overactivity (DO) jeopardizing the upper urinary tract over a 1-year time course assessed by: urodynamic investigation and clinical assessment**	
DO amplitude ≥40 cmH_2O, or initiation of DO treatment with antimuscarinics and/or intradetrusor onabotulinumtoxinA injections (time-to-event analysis)	S&B, 3M, 6M, 12M
**Differential changes in lower urinary tract (LUT) function, assessed by urodynamic investigation**	
Maximum cystometric capacity (mL)	S&B, 3M, 6M, 12M
Compliance (mL/cmH_2O)	S&B, 3M, 6M, 12M
DO (present or absent), if present:	S&B, 3M, 6M, 12M
Bladder volume at first DO (mL)	S&B, 3M, 6M, 12M
Maximum DO amplitude (cmH_2O)	S&B, 3M, 6M, 12M
Detrusor leak-point pressure (cmH_2O)	S&B, 3M, 6M, 12M
Maximum detrusor pressure during storage phase (cmH_2O)	S&B, 3M, 6M, 12M
Maximum detrusor pressure during voiding phase (cmH_2O)	S&B, 3M, 6M, 12M
Detrusor pressure at maximum flow rate (cmH_2O)	S&B, 3M, 6M, 12M
Maximum flow rate (mL/s)	S&B, 3M, 6M, 12M
Voided volume (mL)	S&B, 3M, 6M, 12M
Post void residual (mL)	S&B, 3M, 6M, 12M
Pelvic floor electromyography (EMG) (normal/pathological)	S&B, 3M, 6M, 12M
Videography (vesico-uretero-renal reflux, present/absent)	S&B, 3M, 6M, 12M
Free flow curve pattern	S&B, 3M, 6M, 12M
**Differential changes in lower spinal cord function, assessed by neuro-urological physical examination**	
Voluntary sphincter contraction (present/absent; if present, weak/strong)	S&B, 3M, 6M, 12M
Resting anal tonus (weak/strong/spastic)	S&B, 3M, 6M, 12M
Bulbocavernosus reflex(present/absent)	S&B, 3M, 6M, 12M
Anal reflex (present/absent)	S&B, 3M, 6M, 12M
Sensory testing S2-S5 (algesia, aesthesia: present/absent, even between sides, type of sensation)	S&B, 3M, 6M, 12M
**Differential changes in LUT symptoms, assessed by bladder diary, International Prostate Symptom Score (IPSS), Urinary Symptom Profile (USP)**	
Number of voids per 24 hours	S&B, 3M, 6M, 12M, TTNS: bi-weekly
Number of day-time voids	S&B, 3M, 6M, 12M, TTNS: bi-weekly
Number of night-time voids	S&B, 3M, 6M, 12M, TTNS: bi-weekly
Number of intermittent catheterizations	S&B, 3M, 6M, 12M, TTNS: bi-weekly
Number of leakages per 24 hours	S&B, 3M, 6M, 12M, TTNS: bi-weekly
Number of used pads per 24 hours	S&B, 3M, 6M, 12M, TTNS: bi-weekly
Mean post void residual (mL)	S&B, 3M, 6M, 12M, TTNS: bi-weekly
Mean voided volume (mL)	S&B, 3M, 6M, 12M, TTNS: bi-weekly
Pain level (scale, 0-10)	S&B, 3M, 6M, 12M, TTNS: bi-weekly
Level of urgency (scale, 0-4)	S&B, 3M, 6M, 12M, TTNS: bi-weekly
IPSS Symptom Score (sum score, 0-35)	S&B, 3M, 6M, 12M, TTNS: weekly
USP Stress urinary incontinence score (sum score, 0-9)	S&B, 3M, 6M, 12M, TTNS: weekly
USP Overactive bladder score (sum score, 0-21)	S&B, 3M, 6M, 12M, TTNS: weekly
USP Low stream score (sum score, 0-9)	S&B, 3M, 6M, 12M, TTNS: weekly

**Differential changes in quality of life, assessed by IPSS, Qualiveen**	
IPSS Quality of life score (scale, 0-6)	S&B, 3M, 6M, 12M, TTNS: weekly
Qualiveen total score (mean score, 0-4)	S&B, 3M, 6M, 12M, TTNS: weekly
Qualiveen Limitations / Inconvenience subscale (mean score, 0-4)	S&B, 3M, 6M, 12M, TTNS: weekly
Qualiveen Constraints / Restrictions subscale (mean score, 0-4)	S&B, 3M, 6M, 12M, TTNS: weekly
Qualiveen Fears subscale (mean score, 0-4)	S&B, 3M, 6M, 12M, TTNS: weekly
Qualiveen Feelings / Impact on Daily Life subscale (mean score, 0-4)	S&B, 3M, 6M, 12M, TTNS: weekly
**Differential changes in sexual function, assessed by clinical assessment, FSFI (females), IIEF (males)**	
Psychogenic erection, M (present / reduced / absent / unknown / not applicable)	S&B, 3M, 6M, 12M
Reflex erection, M (present / reduced / absent / unknown / not applicable)	S&B, 3M, 6M, 12M
Ejaculation, M (present / reduced / absent / unknown / not applicable)	S&B, 3M, 6M, 12M
Orgasmic function, M, F (present / reduced / absent / unknown / not applicable)	S&B, 3M, 6M, 12M
Psychogenic genital arousal, F (present / reduced / absent / unknown / not applicable)	S&B, 3M, 6M, 12M
Reflex genital arousal, F (present / reduced / absent / unknown / not applicable)	S&B, 3M, 6M, 12M
Menstruation, F (present / reduced / absent / unknown / not applicable)	S&B, 3M, 6M, 12M
FSFI, F total score (sum score, 0-36)	S&B, 3M, 6M, 12M
FSFI, F Desire domain (sum score, 0-6)	S&B, 3M, 6M, 12M
FSFI, F Arousal domain (sum score, 0-6)	S&B, 3M, 6M, 12M
FSFI, F Lubrication domain (sum score, 0-6)	S&B, 3M, 6M, 12M
FSFI, F Orgasm domain (sum score, 0-6)	S&B, 3M, 6M, 12M
FSFI, F Satisfaction domain (sum score, 0-6)	S&B, 3M, 6M, 12M
FSFI, F Pain domain (sum score, 0-6)	S&B, 3M, 6M, 12M
IIEF, M total score (sum score, 5-75)	S&B, 3M, 6M, 12M
IIEF, M Erectile Function domain (sum score, 1-30)	S&B, 3M, 6M, 12M
IIEF, M Orgasmic Function domain (sum score, 0-10)	S&B, 3M, 6M, 12M
IIEF, M Sexual Desire domain (sum score, 2-10)	S&B, 3M, 6M, 12M
IIEF, M Intercourse Satisfaction domain (sum score, 0-15)	S&B, 3M, 6M, 12M
IIEF, M Overall Satisfaction domain (sum score, 2-10)	S&B, 3M, 6M, 12M
**Differential changes in bowel symptoms, assessed by NBD, bowel diary**	
NBD Score (version from ISCoS Bowel Function Dataset) (sum score, 0-47)	S&B, 3M, 6M, 12M, TTNS: weekly
Number of defecations per 24h	S&B, 3M, 6M, 12M, TTNS: bi-weekly
Number of urgency episodes per 24h	S&B, 3M, 6M, 12M, TTNS: bi-weekly
Quantity of stool per defecation (Small/Medium/Large)	S&B, 3M, 6M, 12M, TTNS: bi-weekly
Feeling of incomplete evacuation per 24h	S&B, 3M, 6M, 12M, TTNS: bi-weekly
Number of accidental bowel leakages per 24h	S&B, 3M, 6M, 12M, TTNS: bi-weekly
Quantity per bowel leakage (Small/Medium/Large)	S&B, 3M, 6M, 12M, TTNS: bi-weekly
Stool consistency score (Bristol Stool Scale)	S&B, 3M, 6M, 12M, TTNS: bi-weekly
Pain related to defecation (score)	S&B, 3M, 6M, 12M, TTNS: bi-weekly
Medications for bowel	S&B, 3M, 6M, 12M, TTNS: bi-weekly
**Differential changes in bowel function and pelvic floor function, assessed by rectal sensitivity examination, anorectal manometry, and barostat assessment**	
Initial sensation (mL)	S&B, 3M, 6M, 12M
Urgency to defecate (mL)	S&B, 3M, 6M, 12M
Maximum tolerated volume (mL)	S&B, 3M, 6M, 12M
Basal internal anal sphincter pressure (mmHg)	S&B, 3M, 6M, 12M
Squeeze external anal sphincter pressure (mmHg)	S&B, 3M, 6M, 12M
Relaxation internal anal sphincter pressure during defecation (%, mmHg)	S&B, 3M, 6M, 12M
Intraabdominal pressure during defecation (mmHg)	S&B, 3M, 6M, 12M

Defecatory disorder (Rao Classification I-IV)	S&B, 3M, 6M, 12M
Rectal capacity (mL)	S&B, 3M, 6M, 12M
Rectal compliance (mL/mmHg)	S&B, 3M, 6M, 12M
**Differential changes in motor and sensory function, assessed by the International Standards for Neurological Classification of Spinal Cord Injury (ISNCSCI) protocol**	
American Spinal Injury Association (ASIA) Impairment scale (AIS) Grade (A,B,C,D,E)	S&B, 3M, 6M, 12M
ISNCSCI upper extremity motor score (UEMS) (score, 0-50)	S&B, 3M, 6M, 12M
ISNCSCI lower extremity motor score (LEMS) (score, 0-50)	S&B, 3M, 6M, 12M
ISNCSCI light touch (LT) score (score, 0-112)	S&B, 3M, 6M, 12M
ISNCSCI pin prick (PP) score (score, 0-112)	S&B, 3M, 6M, 12M
**Differential changes in functional independence assessed by the Spinal Independence Measure (SCIM) III**	
SCIM III Score (sum score, 0-100)	S&B, 3M, 6M, 12M
**Differential changes in spasticity, assessed by Spinal Cord Injury Spasticity Evaluation Tool (SCI-SET), Modified Ashworth Scale (MAS)**	
SCI-SET Score (sum score, -105 to +105; mean score: -3 to +3, number of positive responses, number of negative responses)	S&B, 3M, 6M, 12M, TTNS: weekly
Muscle tone of knee flexors and extensors (scale, 0-4)	S&B, 3M, 6M, 12M
Muscle tone of elbow flexors and extensors (scale, 0-4)	S&B, 3M, 6M, 12M
**Differential changes in brain and spinal cord function as well as cortico-spinal integrity, assessed using neurophysiological and neuroimaging methods: nerve conduction studies (NCS), EMG, electroencephalography (EEG), transcranial magnetic stimulation (TMS)**	
Latencies and amplitudes of F-waves to electrical stimulation	S&B, 3M, 6M, 12M
Compound muscle action potentials (cMAP)	S&B, 3M, 6M, 12M
Current perception thresholds (CPTs)	S&B, 3M, 6M, 12M
Sensory evoked potentials (SEPs): latencies and amplitudes in response to electrical nerve stimulation	S&B, 3M, 6M, 12M
Long latency sensory evoked potentials (LL-SEPs): latencies and amplitudes, in response to electrical nerve stimulation	S&B, 3M, 6M, 12M
Sympathetic skin response (SSR): latencies and amplitudes	S&B, 3M, 6M, 12M
Frequency and coherence measures of surface EMG and EEG	S&B, 3M, 6M, 12M
Lower urinary tract sensory evoked potentials (LUTSEPs): latencies and amplitudes in response to electrical LUT stimulation	S&B, 3M, 6M, 12M
Changes in white and grey matter area in the lumbosacral enlargement (LSE) and their relation to clinical outcomes	S&B, 3M, 6M, 12M
Changes in white and grey matter area in upper cervical spinal cord (at C2/C3M) and their relation to clinical outcomes	S&B, 3M, 6M, 12M
Changes in white and grey matter volume of the conus medullaris (CM) and their relation to clinical outcomes	S&B, 3M, 6M, 12M
Changes in grey and white matter volume in the brain and their relation to clinical outcomes	S&B, 3M, 6M, 12M
Changes in fractional anisotropy (FA) in the brain and spinal cord and their relation to clinical outcomes	S&B, 3M, 6M, 12M
Changes in diffusivity in the brain and spinal cord and their relation to clinical outcomes	S&B, 3M, 6M, 12M
Changes in brain and spinal cord tissue microstructure and their relation to clinical outcomes	S&B, 3M, 6M, 12M
Changes in brain function and connectivity during rest and electrical stimulation of the tibial nerve and their relation to clinical outcomes	S&B, 3M, 6M, 12M
**Differential changes in biochemical and molecular biomarkers in urine, blood, stool, and bladder tissue, assessed by histomorphology, enzyme-linked immunosorbent assay (ELISA), western blot, reverse transcription polymerase chain reaction (RT-PCR), microbiome analysis**	
Biomarkers reflecting oxidative stress (presence/absence and quantities of peptides, proteins, antibodies, and small molecules)	S&B, 3M, 6M, 12M
Expression profile of messenger RNA (mRNA) and microRNA (miRNA) in urine and blood as well as their relation to clinical outcomes	S&B, 3M, 6M, 12M
Changes in inflammatory markers in bladder tissue, blood, and urine, as well as their relation to clinical outcomes	S&B, 3M, 6M, 12M
Changes in levels of neurotransmitters (neurotrophins) in blood, and urine, as well as their relation to clinical outcomes	S&B, 3M, 6M, 12M
Changes in the composition of urinary and stool microbiomes (analysed by sequencing and microbial culturing) and their relation to clinical outcomes	S&B, 3M, 6M, 12M
Changes in the density / quality of afferent / efferent innervation of the bladder tissue and their relation to clinical outcomes	S&B 6M

**Safety of TTNS, adverse events and serious adverse events as assessed by clinical record check and patient interview**	
Urinary tract infection (UTI) (number of occurrences and intensity/severity)	Any time
Infection other than UTI (number of occurrences and intensity/severity)	Any time
Skin irritation at the electrode site (number of occurrences and intensity/severity)	Any time
Irritation-related symptoms (e.g. dysuria, hematuria) with a temporal link to a LUT intervention (i.e. immediately following the intervention) (number of occurrences and intensity/severity)	Any time
Neuro-urological deterioration (number of occurrences and intensity/severity)	Any time
Pressure ulcer (location, number of occurrences and intensity/severity)	Any time
Neurological deterioration (number of occurrences and intensity/severity)*	Any time
Increase in pain (number of occurrences and intensity/severity)*	Any time
Increase in spasticity (number of occurrences and intensity/severity)*	Any time
Deep vein thrombosis / pulmonary embolism (number of occurrences and intensity/severity)	Any time
Autonomic dysreflexia (number of occurrences (suspected and verified) and intensity/severity)	Any time
Transfer/admittance to an acute care facility (number of occurrences and intensity/severity)*	Any time

*only events that qualify as adverse events and serious adverse events are recorded
cMAP compound muscle action potentials; CPTs, current perception thresholds; DO, detrusor overactivity; ELISA, enzyme-linked immunosorbent assay; EEG, electroencephalography; EMG, electromyography; F, female; FSFI, Female Sexual Function Index; IIEF, International Index of Erectile Dysfunction; IPSS, International Prostate Symptom Score; ISNCSCI, the International Standards for Neurological Classification of Spinal Cord Injury; LL-SEP, long latency sensory evoked potential; LUT, lower urinary tract; LUTSEP lower urinary tract sensory evoked potential; M, male; MAS, Modified Ashworth Scale; miRNA, microRNA; mRNA, messenger RNA; NBD, neurogenic bowel dysfunction; NCS, nerve conduction studies; RT-PCR, Reverse transcription polymerase chain reaction; S&B, screening and baseline; SCI, spinal cord injury; SCI-SET, Spinal Cord Injury Spasticity Evaluation Tool; SCIM, Spinal Cord Independence Measure; SEP, sensory evoked potentials; SSR, Sympathetic skin response; TMS, transcranial magnetic stimulation; TTNS, transcutaneous tibial nerve stimulation; USP Urinary Symptom Profile; UTI, urinary tract infection
