# Supplementary material for: TASCI—transcutaneous tibial nerve stimulation in patients with acute spinal cord injury to prevent neurogenic detrusor overactivity: protocol for a nationwide, randomised, sham-controlled, double-blind clinical trial
Source: BMJ Open. 2020 Aug 13;10(8):e039164. doi: 10.1136/bmjopen-2020-039164 (PMC7430472; doi:10.1136/bmjopen-2020-039164)
Supplement: Author's manuscript [file bmjopen-2020-039164.draft_revisions.pdf]

BMJ Open is committed to open peer review. As part of this commitment we make the peer review history of every article we publish publicly available.

When an article is published we post the peer reviewers' comments and the authors' responses online. We also post the versions of the paper that were used during peer review. These are the versions that the peer review comments apply to.

The versions of the paper that follow are the versions that were submitted during the peer review process. They are not the versions of record or the final published versions. They should not be cited or distributed as the published version of this manuscript.

BMJ Open is an open access journal and the full, final, typeset and author-corrected version of record of the manuscript is available on our site with no access controls, subscription charges or pay-per-view fees (http://bmjopen.bmj.com).

If you have any questions on BMJ Open's open peer review process please email info.bmjopen@bmj.com

BMJ Open

TASCI: Transcutaneous tibial nerve stimulation in patients with Acute Spinal Cord Injury to prevent neurogenic detrusor overactivity: Protocol for a nationwide, randomised, sham-controlled, double-blind clinical trial

Journal:	*BMJ Open*
Manuscript ID	bmjopen-2020-039164
Article Type:	Protocol
Date Submitted by the Author:	10-Apr-2020

Complete List of Authors:	Birkhäuser, Veronika; Balgrist University Hospital, University of Zürich, Department of Neuro-Urology<br>Liechti, Martina; Balgrist University Hospital, University of Zürich, Department of Neuro-Urology<br>Anderson, Collene; Swiss Paraplegic Research; University of Lucerne, Department of Health Sciences and Medicine<br>Bachmann, Lucas; Medignition Inc., Research Consultants<br>Baumann, Sarah; Balgrist University Hospital, University of Zürich, Department of Neuro-Urology<br>Baumberger, Michael; Swiss Paraplegic Centre, Spinal Cord and Rehabilitation Medicine<br>Birder, Lori; University of Pittsburgh School of Medicine, Neuro-Urology<br>Botter, Sander; Balgrist Campus AG, Swiss Center for Musculoskeletal Biobanking<br>Büeler, Silvan; Balgrist University Hospital, University of Zürich, Department of Neuro-Urology<br>Cruz, Célia; Universidade do Porto, Instituto de Investigação e Inovação em Saúde, Translational Neuro-urology Group; Universidade do Porto, Faculdade de Medicina, Departemento de Biomedicina, Unidade de Biologia Experimental<br>David, Gergely; Balgrist University Hospital, University of Zürich, Department of Neuro-Urology; Balgrist University Hospital, University of Zürich, Spinal Cord Injury Center<br>Freund, Patrick; Balgrist University Hospital, University of Zürich, Spinal Cord Injury Center<br>Friedl, Susanne; Balgrist University Hospital, University of Zürich, Spinal Cord Injury Center<br>Gross, Oliver; Balgrist University Hospital, University of Zürich, Department of Neuro-Urology<br>Hund-Georgiadis , Margret; REHAB Basel, Clinic of neurorehabilitation and paraplegiology<br>Husmann, Knut; Balgrist Campus AG, Swiss Center for Musculoskeletal Biobanking<br>Jordan, Xavier; Clinique romande de réadaptation, Spinal Cord Injury Department<br>Koschorke, Miriam; Balgrist University Hospital, University of Zürich, Department of Neuro-Urology<br>Leitner, Lorenz; Balgrist University Hospital, University of Zürich,

Department of Neuro-Urology
Luca, Eugenia; Clinique romande de réadaptation, Spinal Cord Injury Department
Mehnert, Ulrich; Balgrist University Hospital, University of Zürich, Department of Neuro-Urology
Möhr, Sandra; REHAB Basel, Clinic of neurorehabilitation and paraplegiology
Mohammadzada, Freschta; Balgrist University Hospital, University of Zürich, Spinal Cord Injury Center
Monastyrskaya, Katia; University of Bern, Urology Research Laboratory, DBMR
Pfender, Nikolai; Balgrist University Hospital, University of Zürich, Spinal Cord Injury Center
Pohl, Daniel; University Hospital Zürich, Department of Gastroenterology and Hepatology
Sadri, Helen; Balgrist University Hospital, University of Zürich, Department of Neuro-Urology
Sartori, Andrea; Balgrist University Hospital, University of Zürich, Department of Neuro-Urology; University of Zürich, Institute for Regenerative Medicine
Schubert, Martin; Balgrist University Hospital, University of Zürich, Spinal Cord Injury Center
Sprengel, Kai; University Hospital Zürich, Department of Trauma
Stalder, Stephanie; Balgrist University Hospital, University of Zürich, Department of Neuro-Urology
Stoyanov, Jivko; Swiss Paraplegic Research; University of Lucerne, Department of Health Sciences and Medicine
Stress, Cornelia; Balgrist University Hospital, University of Zürich, Department of Neuro-Urology
Tatu, Aurora; University Hospital Zürich, Department of Gastroenterology and Hepatology
Tawadros, Cécile; Clinique romande de réadaptation, Spinal Cord Injury Department
van der Lely, Stéphanie; Balgrist University Hospital, University of Zürich, Department of Neuro-Urology
Wöllner, Jens; Swiss Paraplegic Centre, Neuro-Urology
Zubler, Veronika; Balgrist University Hospital, Department of Radiology
Curt, Armin; Balgrist University Hospital, University of Zürich, Spinal Cord Injury Center
Pannek, Jürgen; Swiss Paraplegic Centre, Neuro-Urology; Inselspital University Hospital Bern, Department of Urology
Brinkhof, Martin; Swiss Paraplegic Research; University of Lucerne, Department of Health Sciences and Medicine
Kessler, Thomas; Balgrist University Hospital, University of Zürich, Department of Neuro-Urology

Keywords: Neuro-urology < UROLOGY, Urinary incontinences < UROLOGY, Neurological injury < NEUROLOGY, REHABILITATION MEDICINE, Bladder disorders < UROLOGY

SCHOLARONE™
Manuscripts

I, the Submitting Author has the right to grant and does grant on behalf of all authors of the Work (as defined in the below author licence), an exclusive licence and/or a non-exclusive licence for contributions from authors who are: i) UK Crown employees; ii) where BMJ has agreed a CC-BY licence shall apply, and/or iii) in accordance with the terms applicable for US Federal Government officers or employees acting as part of their official duties; on a worldwide, perpetual, irrevocable, royalty-free basis to BMJ Publishing Group Ltd ("BMJ") its licensees and where the relevant Journal is co-owned by BMJ to the co-owners of the Journal, to publish the Work in this journal and any other BMJ products and to exploit all rights, as set out in our licence.

The Submitting Author accepts and understands that any supply made under these terms is made by BMJ to the Submitting Author unless you are acting as an employee on behalf of your employer or a postgraduate student of an affiliated institution which is paying any applicable article publishing charge ("APC") for Open Access articles. Where the Submitting Author wishes to make the Work available on an Open Access basis (and intends to pay the relevant APC), the terms of reuse of such Open Access shall be governed by a Creative Commons licence – details of these licences and which Creative Commons licence will apply to this Work are set out in our licence referred to above.

Other than as permitted in any relevant BMJ Author's Self Archiving Policies, I confirm this Work has not been accepted for publication elsewhere, is not being considered for publication elsewhere and does not duplicate material already published. I confirm all authors consent to publication of this Work and authorise the granting of this licence.

For peer review only

Target Journal: BMJ Open

TASCI: Transcutaneous tibial nerve stimulation in patients with Acute Spinal Cord Injury to prevent neurogenic detrusor overactivity: Protocol for a nationwide, randomised, sham-controlled, double-blind clinical trial

Veronika Birkhäuser[1,a], Martina D. Liechti[1,a], Collene E. Anderson[1,2,3,a], Lucas M. Bachmann[4], Sarah Baumann[1], Michael Baumberger[5], Lori A. Birder[6], Sander M. Botter[7], Silvan Büeler[1], Célia D. Cruz[8,9], Gergely David[1,10], Patrick Freund[10], Susanne Friedl[10], Oliver Gross[1], Margret Hund-Georgiadis[11], Knut Husmann[7], Xavier Jordan[12], Miriam Koschorke[1], Lorenz Leitner[1], Eugenia Luca[12], Ulrich Mehnert[1], Sandra Möhr[11], Freschta Mohammadzada[10], Katia Monastyrskaya[13], Nikolai Pfender[10], Daniel Pohl[14], Helen Sadri[1], Andrea M. Sartori[1,15], Martin Schubert[10], Kai Sprengel[16], Stephanie A. Stalder[1], Jivko Stoyanov[2,3], Cornelia Stress[1], Aurora Tatu[14], Cécile Tawadros[12], Stéphanie van der Lely[1], Jens Wöllner[17], Veronika Zubler[18], Armin Curt[10], Jürgen Pannek[17,19], Martin W.G. Brinkhof[2,3], Thomas M. Kessler[1,#]

Author affiliations

[1] Department of Neuro-Urology, Balgrist University Hospital, University of Zürich, Zürich, Switzerland

[2] Swiss Paraplegic Research, Nottwil, Switzerland

[3] Department of Health Sciences and Medicine, University of Lucerne, Lucerne, Switzerland

[4] Medignition Inc., Research Consultants, Zürich, Switzerland

[5] Spinal Cord and Rehabilitation Medicine, Swiss Paraplegic Centre, Nottwil, Switzerland

[6] Neuro-Urology, University of Pittsburgh School of Medicine, Pittsburgh, PA, USA

[7] Swiss Center for Musculoskeletal Biobanking, Balgrist Campus AG, Zürich, Switzerland

[8] Instituto de Investigação e Inovação em Saúde, Translational Neuro-urology Group, Universidade do Porto, Porto, Portugal

For peer review only - http://bmjopen.bmj.com/site/about/guidelines.xhtml

[9] Faculdade de Medicina, Departemento de Biomedicina, Unidade de Biologia Experimental, Universidade do Porto, Porto, Portugal

[10] Spinal Cord Injury Center, Balgrist University Hospital, University of Zürich, Zürich, Switzerland

[11] REHAB Basel, Clinic of neurorehabilitation and paraplegiology, Basel, Switzerland

[12] Spinal Cord Injury Department, Clinique romande de réadaptation, Sion, Switzerland

[13] Urology Research Laboratory, DBMR, University of Bern, Bern, Switzerland

[14] Department of Gastroenterology and Hepatology, University Hospital Zürich, Zürich, Switzerland

[15] Institute for Regenerative Medicine, University of Zürich, Zürich, Switzerland

[16] Department of Trauma, University Hospital Zürich, Zürich, Switzerland

[17] Neuro-Urology, Swiss Paraplegic Centre, Nottwil, Switzerland

[18] Department of Radiology, Balgrist University Hospital, Zürich, Switzerland.

[19] Department of Urology, Inselspital, Bern University Hospital, Bern, Switzerland

# Corresponding author: Prof. Thomas M. Kessler, MD
Department of Neuro-Urology
Balgrist University Hospital
University of Zürich
Forchstrasse 340
8008 Zürich
Switzerland
Email: tkessler@gmx.ch

[a] VB, MDL, and CEA contributed equally

For peer review only

Word count: 3696

Key Words (MeSH): Urinary Bladder, Neurogenic; Urinary Bladder, Overactive; Transcutaneous Electric Nerve Stimulation; Spinal Cord Injuries; Clinical Trial Protocol

For peer review only - http://bmjopen.bmj.com/site/about/guidelines.xhtml

ABSTRACT

Introduction: Neurogenic lower urinary tract dysfunction (NLUTD), including neurogenic detrusor overactivity (NDO) and detrusor-sphincter dyssynergia, is one of the most frequent and devastating sequelae of spinal cord injury (SCI), as it can lead to urinary incontinence and secondary damage such as renal failure. Transcutaneous tibial nerve stimulation (TTNS) is a promising, non-invasive neuromodulatory intervention that may prevent the emergence of the C-fibre evoked bladder reflexes that are thought to cause NDO. This paper presents the protocol for TASCI (TTNS in Acute SCI), which will evaluate the efficacy of TTNS in preventing NDO. Furthermore, TASCI will provide insight into the mechanisms underlying TTNS, and the course of NLUTD development after SCI.

Methods and analysis: TASCI is a nationwide, randomised, sham-controlled, double-blind clinical trial, conducted at all four SCI centres in Switzerland. The longitudinal design includes a baseline assessment period 5-39 days after acute SCI and follow-up assessments occurring three, six, and twelve months after SCI. A planned 114 participants will be randomised into verum or sham TTNS groups (1:1 ratio), stratified on study centre and lower extremity motor score. TTNS is performed for 30-minutes per day, five days per week, for 6-9 weeks starting within 40 days after SCI. The primary outcome is the occurrence of NDO jeopardizing the upper urinary tract at one year after SCI, assessed by urodynamic investigation. Secondary outcome measures assess bladder and bowel function and symptoms, sexual function, neurological structure and function, functional independence, quality of life, as well as changes in biomarkers in the urine, blood, stool, and bladder tissue. Safety of TTNS is the tertiary outcome.

Ethics and dissemination: TASCI is approved by the Swiss Ethics Committee for Northwest/Central Switzerland, the Swiss Ethics Committee Vaud and the Swiss

For peer review only - http://bmjopen.bmj.com/site/about/guidelines.xhtml

Ethics Committee Zürich (#2019-00074). Findings will be disseminated through peer-reviewed publications.

Trial registration number: NCT03965299, ClinicalTrials.gov.

Article Summary: Strengths and limitations of this study

- This study is a nationwide, randomised, sham-controlled, double-blind clinical trial conducted at all four spinal cord injury (SCI) centres in Switzerland.
- Transcutaneous tibial nerve stimulation (TTNS) is a well-tolerated, low-risk, non-invasive intervention.
- The inpatient setting facilitates study procedures and monitoring that maximize intervention adherence.
- The design of the intervention procedure ensures the blinding of the participants and outcome assessors; however, in order to ensure that participants receive an optimal dosage of electrical stimulation, the TTNS operators need to remain unblinded.
- Secondary outcomes provide valuable information regarding the impact of TTNS on additional factors that are relevant to patients as well as clinicians, and also facilitate targeted analyses aimed at better understanding the mechanism underlying TTNS therapy, and the development of neurogenic lower urinary tract dysfunction after SCI.

For peer review only

INTRODUCTION

Most patients with spinal cord injury (SCI) progressively develop neurogenic lower urinary tract dysfunction (NLUTD),[1] which is one of the most devastating sequelae of SCI. Recovery of lower urinary tract (LUT) function is often identified as a top patient concern after SCI, and sometimes is even prioritized above walking.[2-4] The consequences of NLUTD, including urinary incontinence, dependence on catheters, and recurrent urinary tract infections, negatively impact quality of life (QoL).[5-7] On a neurological level, intact pathways between the brain and the LUT are required for the complex multilevel neural network governing the LUT to function properly.[8] SCI is thought to trigger neuroplastic changes in C-fibre-mediated spinal reflex pathways in the spinal cord, which lead to the emergence of aberrant micturition reflexes.[9 10] Thus, within a few weeks after acute SCI, most patients start experiencing involuntary bladder contractions, neurogenic detrusor overactivity (NDO), which can cause urinary incontinence.[8] Furthermore, NDO, in combination with detrusor sphincter dyssynergia (DSD) and/or low bladder compliance, can produce high intravesical pressure, which is a relevant risk factor for renal failure.[11 12] NDO also predisposes patients to vesico-uretero-renal reflux, which can endanger the upper urinary tract (UUT).[8 13] Accordingly, the current urological management approach is centred around protecting the UUT and treating long-term complications of NLUTD.[13 14] Antimuscarinic medication and intradetrusor onabotulinumtoxinA injections are the first- and second-line treatments for NDO, but both treatments are often discontinued due to limited effectiveness and bothersome side effects.[15-17] If conventional conservative and minimally invasive therapies fail, the only remaining options are more invasive treatments which are associated with substantial morbidity, such as bladder augmentation and urinary diversion.[18 19] While neuro-urological

For peer review only - http://bmjopen.bmj.com/site/about/guidelines.xhtml

treatment of patients with SCI has greatly improved during the last decades, it is only applied when NLUTD is already established with little chance of recovery.

Here we present the protocol for a randomised controlled trial (RCT) that aims at producing evidence for the use of non-invasive electrical stimulation (neuromodulation) to prevent the development of NDO. Early neuromodulatory therapy has the potential to impede or alter neuroplasticity, preventing the sensitisation of 'silent' C fibres and the formation of spinal reflex pathways thought to be responsible for NDO. Indeed, in a pilot study, bilateral sacral neuromodulation during the early phase of SCI prevented the development of NDO and also improved erectile and bowel function.[20] Furthermore, in animal models, early neuromodulatory interventions after SCI can modulate the micturition reflex[21] and inhibit detrusor overactivity.[22] In humans, non-invasive neuromodulatory techniques such as tibial nerve stimulation[23] and transcutaneous electrical nerve stimulation (TENS)[24] have shown promise in a treatment context[25-28] and warrant well-designed RCTs. Transcutaneous tibial nerve stimulation (TTNS) has generated interest, as a neuromodulatory treatment that is completely non-invasive (current is applied through surface electrodes) with no known safety risks, easy to apply, and well-accepted by patients. A randomised pilot trial in patients with acute SCI concluded that TTNS was feasible and safe in an inpatient rehabilitation setting.[29] Furthermore, at the end of the study, the participants in the intervention group had lower detrusor pressures and fewer developed DSD compared to the sham group,[30] suggesting that TTNS could alter the course of NLUTD. We hypothesize that TTNS applied in the acute phase after SCI will prevent the emergence of C-fibre evoked bladder reflexes, thereby the development of NDO, and avert UUT damage and urinary incontinence. TASCI (TTNS in Acute SCI) aims to provide evidence regarding the clinical efficacy of TTNS as a preventative measure for NDO, along with additional insight into the mechanism

For peer review only - http://bmjopen.bmj.com/site/about/guidelines.xhtml

underlying TTNS and the course of NLUTD development during the first year after SCI. We operationalized this RCT as a population-based study in the context of specialized rehabilitation for SCI in Switzerland.

METHODS AND ANALYSES

This protocol follows the recommendations of the SPIRIT (Standard Protocol Items: Recommendations for Interventional Trials) Statement (Supplement 1).[31]

Study design and setting

TASCI is a nationwide, randomised, sham-controlled, double-blind clinical trial investigating the effects of TTNS in patients with acute SCI.[32] The intervention will take place during the inpatient rehabilitation stay in one of the four SCI centres in Switzerland (Basel: REHAB Basel, Nottwil: Swiss Paraplegic Center, Sion: Clinique romande de réadaptation, and Zürich: Balgrist University Hospital). Furthermore, TASCI is nested in the inception cohort of the prospective Swiss Spinal Cord Injury (SwiSCI) cohort study.[33]

The TASCI schedule (Figure 1) includes a screening and baseline period from days 5-39 after SCI diagnosis, followed by a 6-9-week intervention period that ends when all 3-month follow-up assessments have been completed. Three follow-up visits are planned: the 3-month visit (day 91 +/- 10), 6-month visit (day 182 +/- 10), and 12-month visit (day 365 +/- 10). The one-year post-SCI measurement time point was chosen for the primary outcome variable because it represents the time when the patient's situation is stable enough to start considering invasive treatment options.[14] The additional time points were selected to maximize the chances of detecting treatment effects, as well as to coincide with routine follow-up assessments of patients with SCI.

For peer review only - http://bmjopen.bmj.com/site/about/guidelines.xhtml

Participants

All SCI patients admitted to rehabilitation in one of the participating centres are screened for eligibility using information from their routine clinical examinations. For a full listing of inclusion and exclusion criteria refer to Box 1. Eligible patients are then approached by an investigator or supervising clinician who obtains informed consent. Patients who consent undergo additional baseline assessments and are randomised after their eligibility for the trial has been confirmed.

For peer review only

For peer review only - http://bmjopen.bmj.com/site/about/guidelines.xhtml

Box 1. TASCI eligibility criteria

Inclusion Criteria	Exclusion Criteria
➢ Age ≥ 18 years<br>➢ Patients with acute SCI (traumatic SCI and sudden onset (<7 days) non-traumatic SCI), within 40 days after injury<br>➢ Patients with acute SCI at cervical or thoracic level<br>➢ Willingness to take part and follow the requirements of the protocol during TASCI (up to 1 year after SCI):<br>• No percutaneous tibial nerve stimulation (PTNS)<br>• No functional electrical stimulation (FES) apart from upper limb FES<br>• No electrical muscle stimulation (EMS)<br>➢ Informed consent	➢ Contraindications to the investigational product<br>➢ Treatment with antimuscarinics or mirabegron<br>➢ DO with contractions greater than 40 cmH_2O at a bladder filling volume of less than 500 mL at baseline visit<br>➢ Known or suspected non-adherence, drug or alcohol abuse<br>➢ Inability to follow the procedures of the study, e.g. due to language problems, psychological disorders, dementia, etc. of the participant<br>➢ Participation in another study with investigational drug or product within the 30 days preceding and during the present study<br>➢ Neuromodulation treatment for urological or bowel indication in the last six months or ongoing<br>➢ Botulinum toxin injections in the detrusor and/or urethral sphincter in the last six months<br>➢ Bilaterally absent tibial nerve compound muscle action potential (cMAP, amplitude < 1mV)<br>➢ Women who are pregnant or breast feeding<br>➢ Intention to become pregnant during the course of the study<br>➢ Individuals especially in need of protection (according to Research with Human Subjects published by the Swiss Academy of Medical Sciences [www.samw.ch/en/News/News.html])<br>➢ Enrolment of the investigator, their family members, employees and other dependent persons<br>➢ Pre-existing or concomitant medical condition apart from SCI that might pose a safety issue or would interfere with interpretation of study results or study conduct (e.g. Parkinson's disease, neurodegenerative disorders including multiple sclerosis and amyotrophic lateral sclerosis, urological malignancies)

Urinary tract infection (UTI) is a transitory exclusion criterion for all functional assessments, including urodynamics. Before undergoing functional assessments, participants are screened for UTIs and haematuria. If a UTI is identified, treatment is initiated, and the functional assessments are rescheduled once the participant has been symptom-free for at least three days. DO, detrusor overactivity; SCI, spinal cord injury; TASCI, transcutaneous tibial nerve stimulation in acute spinal cord injury

For peer review only

For peer review only - http://bmjopen.bmj.com/site/about/guidelines.xhtml

Randomisation and blinding

Participants are randomly allocated with a 1:1 ratio into TTNS verum or TTNS sham stimulation groups, using computer-generated permuted block randomisation lists with varying block sizes. The randomisation lists are stratified according to study centre and the primary prognostic indicator, lower extremity motor score (LEMS) from the International Standards for Neurological Classification of SCI (ISNCSCI) assessment.[34] Prognostic models have shown that LEMS measured within 40 days of SCI is a reliable predictor of urinary continence and complete bladder emptying one year after SCI.[35] Based on this model, LEMS cut-offs of <12, 12-37, and >37 were chosen, which correspond to a population-average estimated probability of <20%, 20%-80% and >80% for urinary continence and complete bladder emptying at 1 year after SCI.[35] The allocation sequence and assignment of participants is centrally coordinated, and performed via the database software (Research Electronic Data Capture, REDCap[36 37]). To safeguard the allocation concealment, the randomisation table will be password secured and only accessible to investigators responsible for randomisation and application of the TTNS intervention.

To maintain blinding of the participants, care providers, and outcome assessors, throughout the duration of the participants' involvement in the study, the randomisation and TTNS intervention will be performed by trained operators, who are not involved in the clinical management. Additionally, the verum and sham TTNS setups are identical in terms of appearance, application procedure, and intervention duration, and all participants receive the same information.

Intervention

A commercially available TENS device (ELPHA II 3000, CE 0543 Certification, FH Service, Odense, Denmark) is used for both the TTNS verum and sham

For peer review only - http://bmjopen.bmj.com/site/about/guidelines.xhtml

intervention. The current frequency (20 Hz) and pulse width (200 μs) are pre-programmed (Figure 2). The current amplitude, which can be set between 0 and 100 mA, is manually adjusted for each participant during the initial set-up period. Each TTNS session starts with a preparatory phase where the participant is asked to lie in a comfortable supine position and a pillow or blanket is placed on the lower part of the leg to restrict the participant's view of their foot. Four electrodes are placed according to a pre-specified order on the foot and lower leg. The first electrode is placed 4-5cm above and 4-5cm posterior to the medial malleolus. The next two electrodes are placed around the 5th metatarsophalangeal joint, one dorsally over the bone, and one on the plantar fat pad. A final electrode is placed distal to, and in line with, the medial malleolus in the longitudinal arch of the foot (Figure 2). During an initial test period of several minutes, sensory and motor thresholds (if applicable) are assessed, current amplitudes are adjusted and the operator is carefully observing foot movement, i.e. flexion or fanning of the big toe or any other motor response. The operator may reposition electrodes to optimise the electrical stimulation setup. After the correct positioning of the electrodes has been verified by observation of the appropriate motor response,[23] the stimulation amplitude is reduced to a sub-motor level, defined as 0.5-1.0 mA below the motor threshold. In the sham setup, a cable with an interposed 1000 Ω resistance allows a current amplitude to be displayed and changed on the device without providing electrical stimulation to the participant.

The TTNS intervention is performed for 30 minutes per day, five days per week, starting within 40 days after SCI and ending after all 3-month follow-up assessments have been completed (total intervention period: 6-9 weeks). The majority of the participants are expected to be inpatients for the duration of the intervention, a setting that maximizes participant adherence. The application of the TTNS intervention by designated and trained personnel ensures that the treatment is

For peer review only - http://bmjopen.bmj.com/site/about/guidelines.xhtml

applied correctly while blinding is maintained. In addition, the remaining follow-up visits are part of standardized SCI care in all four participating centres, limiting the potential loss to follow-up, and ensuring that the participants receive necessary ancillary care.

Participants may be withdrawn when the study protocol is not followed, a health problem occurs with a suspected link to the stimulation, if a severe health condition/illness makes an intensive outpatient treatment or new hospitalization necessary, consent is withdrawn, or if a pregnancy is detected. Any concomitant treatment and medication intake will be carefully documented, so that the information can be utilized in secondary analyses.

Outcomes

The primary outcome is the occurrence of NDO jeopardizing the upper urinary tract up to 12 months after SCI, i.e. at 1 year after SCI or any earlier time point if NDO treatment is needed. This outcome is a composite measure including: NDO with an amplitude ≥ 40 cmH_2O identified during urodynamic investigation; or else the initiation of NDO treatment with antimuscarinics and/or intradetrusor onabotulinumtoxinA injections. Urodynamics will be performed before starting TTNS (within 40 days after SCI) at follow-up visits three-months, six-months and one year after SCI, or at any earlier time point if there is reason to believe that the participant has developed NDO, before starting NDO treatment.

Secondary outcomes were selected to further evaluate the effects of the TTNS intervention, and include clinical outcomes such as differential changes in LUT and bowel function and symptoms, sexual function, neurological function, as well as functional independence and QoL. Moreover, the evaluation of differential changes in brain and spinal cord function as well as cortico-spinal integrity, and biomarkers in

For peer review only - http://bmjopen.bmj.com/site/about/guidelines.xhtml

the urine, blood, stool, and bladder tissue will also provide insight into the mechanism of action underlying the TTNS intervention. All secondary outcomes may also be used to better understand the pathogenesis of NLUTD after SCI. Information collected will provide a detailed description of the disease course, and when possible, inform prognostic modelling and address causal questions. Finally, adverse events will be recorded for the duration of the participant's enrolment in the study to evaluate the safety of TTNS. Table 1 shows an overview of the outcome measurement assessments and assessment periods, and outcome parameters are listed in detail in Supplement 2.

Generally, the outcome assessments are administered at screening and baseline as well as during each of the three follow-up visits (Figure 1). One notable exception is the bladder biopsy, which will take place at baseline and the 6-month follow-up visit, and only with additional consent. Moreover, during the intervention period, participants will complete some of the questionnaires once per week, and the bladder and bowel diaries once every two weeks. Withdrawal from the study can occur at any time if treatment for NDO is indicated. If withdrawal occurs, all study completion assessments should be performed whenever possible. Furthermore, the trial is nested in the SwiSCI cohort study, so observation of the participants will continue through the cohort study after the end of TASCI. SwiSCI can provide long-term, open-label outcome data regarding morbidity, activities and participation (e.g. labour market participation), QoL and life expectancy.

For peer review only

Table 1. Secondary outcomes and outcome measures. Assessment periods are referenced to the date of spinal cord injury (SCI) diagnosis and include: screening and baseline (S&B) (days 5-39), 3-month follow-up (day 91 +/- 10), 6-month follow-up (day 182 +/-10), 12-month follow-up (day 365 +/-10). Additionally, some surveys are administered weekly during the TTNS treatment period (TTNS: weekly), and the bowel and bladder diaries are administered once every two weeks during the treatment period (TTNS: bi-weekly).

Outcome & Outcome Measure(s)	Assessment Periods
**Occurrence of neurogenic DO jeopardizing the upper urinary tract over a 1-year time course (time-to-event analysis)**	
Urodynamic investigation	S&B, 3, 6, 12 m
Clinical assessment	S&B, 3, 6, 12 m
**Differential changes in lower urinary tract function**	
Urodynamic investigation	S&B, 3, 6, 12 m
**Differential changes in lower urinary tract symptoms**	
Bladder diary	S&B, 3, 6, 12 m; TTNS: bi-weekly
International Prostate Symptom Score (IPSS)	S&B, 3, 6, 12 m; TTNS: weekly
Urinary Symptom Profile (USP)	S&B, 3, 6, 12 m; TTNS: weekly
**Differential changes in neurological structure and function**	
Neuro-urological physical examination	S&B, 3, 6, 12 m
International Standards for Neurological Classification of Spinal Cord Injury (ISNCSCI) assessment	S&B, 3, 6, 12 m
Nerve conduction studies (NCS)	S&B, 3, 6, 12 m
Electromyography (EMG)	S&B, 3, 6, 12 m
Electroencephalography (EEG)	S&B, 3, 6, 12 m
Transcranial magnetic stimulation (TMS)	S&B, 3, 6, 12 m
Magnetic resonance imaging (MRI)	S&B, 3, 6, 12 m
**Differential changes in bowel and pelvic floor function**	
Rectal sensitivity examination	S&B, 3, 6, 12 m
Anorectal manometry	S&B, 3, 6, 12 m
Barostat assessment	S&B, 3, 6, 12 m
**Differential changes in bowel symptoms**	
Bowel diary	S&B, 3, 6, 12 m; TTNS: bi-weekly
Neurogenic Bowel Dysfunction (NBD) Score	S&B, 3, 6, 12 m; TTNS: weekly
**Differential changes in sexual function**	
Female Sexual Function Index (FSFI)	S&B, 3, 6, 12 m
International Index of Erectile Function (IIEF)	S&B, 3, 6, 12 m
Clinical assessment	S&B, 3, 6, 12 m
**Differential changes in spasticity**	
Modified Ashworth Scale (MAS)	S&B, 3, 6, 12 m
Spinal Cord Injury Spasticity Evaluation Tool (SCI-SET)	S&B, 3, 6, 12 m; TTNS: weekly
**Differential changes in functional independence**	
Spinal Cord Independence Measure (SCIM) III	S&B, 3, 6, 12 m
**Differential changes in quality of life**	
International Prostate Symptom Score (IPSS)	S&B, 3, 6, 12 m; TTNS: weekly
Qualiveen	S&B, 3, 6, 12 m; TTNS: weekly
**Differential changes in biochemical and molecular biomarkers in the urine, blood, and stool, as well as in the bladder tissue**	
Histomorphology	S&B, 6 m
Enzyme-linked immunosorbent assay (ELISA)	S&B, 3, 6, 12 m
Western immunoblotting	S&B, 3, 6, 12 m
Reverse transcription polymerase chain reaction (RT-PCR)	S&B, 3, 6, 12 m
Microbiome analysis	S&B, 3, 6, 12 m
**Safety of TTNS**	
Clinical record check	Any time
Medical history	Any time
Health diary, confirmed by interview	Any time

DO, detrusor overactivity; m, months; SCI, spinal cord injury; S&B, screening and baseline; TTNS, transcutaneous tibial nerve stimulation

For peer review only

For peer review only - http://bmjopen.bmj.com/site/about/guidelines.xhtml

Sample size

Presuming a proportion of spontaneous success among the sham group of 0.15,[23] 114 participants (57 verum, 57 sham) need to be included to detect a difference of 0.25 in the proportion of participants who develop NDO, and thereby to correctly reject the null hypothesis with a probability (power) of 0.8. The type I error probability associated with the test of this null hypothesis is 0.05. Since the primary outcome variable is binary, this sample size analysis included a continuity correction to avoid type I error inflation.

Using prospective data collected by the SwiSCI inception cohort for the years 2014-2016 and applying a modified version of the TASCI inclusion/exclusion criteria, a total of 193 eligible patients are predicted during the 3-year recruitment period of TASCI. Anticipating a recruitment rate of 66%, this implies that the requisite 114 participants can be included within 3 years. Expected participant numbers per centre during the study period are 20 for Basel, 42 for Nottwil, 13 for Sion, and 39 for Zürich. Moreover, this data implies that participants who withdraw or drop out can be replaced.

Data collection and management

All investigators are responsible for complete and correct data entry. Data will be recorded in electronic case report forms (eCRFs) using an internet-based, secure database – REDCap,[36 37] which fulfils the Good Clinical Practice (GCP) requirements and is hosted at Balgrist University Hospital. Participants will be assigned a unique 10-digit identification number, and personal data (e.g. name) are not entered into the main study database. Confidentiality will be ensured by restricting the access to the trial database to only a minimal number of people, and by pseudonymisation of the data before analyses. The database includes built-in quality control mechanisms,

For peer review only - http://bmjopen.bmj.com/site/about/guidelines.xhtml

such as range-restricted data entry, and identification of missing data. A monitoring system clearly indicates that an expected assessment is late or missing. Additionally, a manual check is performed of each dataset after it is closed and marked as complete, first by a research assistant at the study site, then by a study coordinator.

Statistical analyses

The primary analysis will be an intention-to-treat analysis. The general statistical methodology for primary and secondary outcomes is comprised of basic descriptive statistics and multivariable regression analyses. Continuous variables will be tested for normality, and displayed as either means and standard deviations or medians and ranges; categorical variables will be displayed as frequencies and proportions. Multivariable regression techniques will be employed to control for possible confounding due to unequal distribution of baseline parameters across the trial arms, as well as systematic variation in outcomes across RCT strata, i.e. centre and LEMS. Longitudinal analysis techniques (e.g. mixed-effects models, structural equation models for growth, and time-to-event models) will be used to account for repeated measures, facilitating time-updated covariate adjustment and appropriately modelling trends with respect to time.

The primary outcome "the occurrence of NDO jeopardizing the upper urinary tract one year after SCI" is a binary response variable, which will be described using proportions with 95% binomial confidence intervals (CIs) and analysed using logistic regression, thus expressing treatment effect sizes as odds ratios (with 95% CIs). Sensitivity analyses will include a time-to-event analysis to evaluate the impact of TTNS on the risk of early drop-out or pre-term endpoints (< 1year). Protocol non-adherence, early withdrawals, and loss to follow-up will be examined and used to inform per-protocol sensitivity analyses. Sub-group analyses stratified on LEMS or

For peer review only - http://bmjopen.bmj.com/site/about/guidelines.xhtml

study centre may be performed if an intervention-subgroup interaction is found, with the understanding that these analyses will likely be underpowered. In cases where outcome data is used in an observational context, usually to examine prognosis or NLUTD aetiology, causal claims will be supported by an underlying theoretical framework.

Multiple imputation will be used when appropriate to account for missing data in the predictor variables.[38] Pattern mixture models may be applied to address missing outcome data for different scenarios of the missing data process.[39] Sensitivity analysis will be used to evaluate the robustness of the chosen imputation strategy. Any sensitivity analyses added after the breaking of the blind (post-hoc sensitivity analyses) will be clearly identified as such in the trial report.

No interim analyses are planned. In the event that severe clinical or neurological deterioration is detected in more than one subject, or on the recommendation of the study monitoring board following a serious adverse event, the study will be suspended until a comprehensive safety review has been completed. If the trial is suspended or halted, an interim analysis will be performed.

Quality assurance and safety oversight

Monitoring will be carried out by the Unit for Clinical and Applied Research (UCAR) at Balgrist University Hospital. Monitoring visits will occur at each study site before the start of the study, after three-month follow-up assessments have been completed for the first participant, and then annually thereafter, with a final closeout visit at study end. The site qualification and initiation visit(s) will assure that the study site has the appropriate facilities and personnel, and that the personnel are able to conduct the study according to protocol. Routine visits will focus on source document verification, in particular documents related to eligibility, consent, key outcome

For peer review only - http://bmjopen.bmj.com/site/about/guidelines.xhtml

parameters, and ensure the documentation and reporting of adverse events occurred in accordance with all legal, ethical and protocol-specific requirements.

Patient and public involvement

Feedback on the intervention procedure was obtained from patients and healthy volunteers during the piloting phase and used to refine the study protocol. All participants will receive a study update newsletter annually.

ETHICS AND DISSEMINATION

Ethics

TASCI will be conducted in accordance with the protocol and the principles contained in the current version of the Declaration of Helsinki, the GCP guidelines issued by the ICH, the European Regulation on medical devices 2017/745 and the ISO Norm 14155 and ISO 14971, the Swiss Law and Swiss regulatory authority's requirements. The study has been approved by the Swiss Ethics Committee for Northwest/Central Switzerland, the Swiss Ethics Committee Vaud and the Swiss Ethics Committee Zürich (2019-00074) and has been registered at ClinicalTrials.gov (NCT03965299). The ethics committees and regulatory authorities will receive annual safety and interim reports and be informed about study end. All investigators will respect the participant's right to confidentiality, and adhere to the Swiss Federal Act on Data Protection. There are no conflicts of interest.

Dissemination

The study results will be published in open-access peer-reviewed journals, and authorship will be defined according to the recommendations of the International Committee of Medical Journal Editors (ICMJE). All persons wishing to publish using

For peer review only - http://bmjopen.bmj.com/site/about/guidelines.xhtml

TASCI data must submit a project plan to the TASCI steering committee for approval before the data are released for analysis. Datasets and statistical coding will be deposited in a public repository, as long as participant privacy is not compromised. Results will be presented at conferences of relevant societies such as the American Urological Association (AUA), Deutschsprachige Medizinische Gesellschaft für Paraplegie (DMGP), European Association of Urology (EAU), International Continence Society (ICS), International Neuro-Urology Society (INUS), International Spinal Cord Society (ISCoS). Furthermore, these societies will be used as platforms to advocate the implementation of the study findings in daily clinical practice and incorporation into clinical guidelines. Dissemination among persons with SCI will be achieved through the newsletters Paraplegie and Paracontact as well as online media.

DISCUSSION

TASCI is the first RCT to investigate the efficacy of TTNS in preventing the development of NDO after acute SCI. It represents a transition in the approach to NLUTD management from treatment of manifested problems to prevention of the disorder itself. The structured follow-up of participants could further inform clinical practice, for example, providing much-needed evidence about the timing of key examinations. Moreover, the inclusion of 'research oriented' secondary outcomes will provide data that may shed some light on the mechanism underlying neuromodulatory therapy, which up until now, is essentially a black box. Additionally, the secondary outcome data could deepen the understanding of the natural trajectory of NLUTD development in the first year after SCI. A final potential benefit of this trial is that it utilizes a multidisciplinary approach to maximise the chances of identifying and understanding the potential preventative effects of neuromodulatory therapy. The

For peer review only - http://bmjopen.bmj.com/site/about/guidelines.xhtml

requisite collaboration among experts in neuro-urology, neuroscience, SCI, rehabilitation medicine, basic and translational research, and biostatistics improves the overall quality of the research and promotes future collaborative endeavours.

The successful development of a therapy that prevents NDO after SCI would represent a huge achievement, especially since there is no known cure, only management of consequences. An effective preventative measure could eliminate lifelong issues associated with SCI, lowering barriers to work force participation and improving QoL, at the same time reducing lifetime healthcare costs. Thus, TASCI represents an important and timely RCT with the potential to revolutionise clinical practice, as well as to provide conceptual insight regarding the mechanism of neuromodulation, and the developmental course of NLUTD following SCI.

Trial status

This publication is based on version 2 of the TASCI protocol dated October 29th, 2019. The official start of recruitment was on June 19th, 2019. The estimated end date of the trial is June 30th, 2024 and recruitment of patients is ongoing. As of the time of submission, temporary interruptions to recruitment due to the COVID-19 pandemic are a possibility and in case of extended disruption, a schedule adjustment may be required.

Acknowledgements: We thank all members of the TASCI Study Group for their support. We thank all the study participants for their involvement in this trial.

Author Contributions: TMK is the chief investigator and responsible for overseeing the trial. AC, JP, MWGB, and TMK are study PIs, were responsible for the study conception and obtained funding. XJ, SM, JP, and TMK are site PIs. The TASCI

For peer review only - http://bmjopen.bmj.com/site/about/guidelines.xhtml

steering committee consists of the study PIs and the site PIs. All authors contributed to the methodology and study protocol. CEA, LMB, and MWGB provided statistical expertise. VB, MDL, CEA, MWGB, and TMK drafted the manuscript. All authors reviewed and revised the manuscript before submission and approved its content.

Funding: This work is financially supported by the Swiss National Science Foundation (SNSF), grant number: SNSF, 33IC30_179644. Start-up funding was provided by the Swiss Paraplegic Foundation. Neither funding body had (or will have) a role in the study design; collection, management, analysis, and interpretation of data; writing of the report; or the decision to submit the report for publication.

Competing interests: None declared.

Data Access Statement: Data generated by this research will be anonymised and uploaded to a public repository after publication wherever legally and ethically possible. When this is not possible, data will be made available upon reasonable request.

Patient consent for publication: Not required.

Ethics approval: The study has been approved by the Swiss Ethics Committee for Northwest/Central Switzerland, the Swiss Ethics Committee Vaud and the Swiss Ethics Committee Zürich (2019-00074) and has been registered at ClinicalTrials.gov (NCT03965299).

For peer review only

REFERENCES

1. Weld KJ, Dmochowski RR. Association of level of injury and bladder behavior in patients with post-traumatic spinal cord injury. *Urology* 2000;55(4):490-4. [published Online First: 2000/03/29]
2. Simpson LA, Eng JJ, Hsieh JT, et al. The health and life priorities of individuals with spinal cord injury: a systematic review. *Journal of neurotrauma* 2012;29(8):1548-55. doi: 10.1089/neu.2011.2226 [published Online First: 2012/02/11]
3. Lo C, Tran Y, Anderson K, et al. Functional Priorities in Persons with Spinal Cord Injury: Using Discrete Choice Experiments To Determine Preferences. *Journal of neurotrauma* 2016;33(21):1958-68. doi: 10.1089/neu.2016.4423 [published Online First: 2016/11/02]
4. Brinkhof MW, Al-Khodairy A, Eriks-Hoogland I, et al. Health conditions in people with spinal cord injury: Contemporary evidence from a population-based community survey in Switzerland. *Journal of rehabilitation medicine* 2016;48(2):197-209. doi: 10.2340/16501977-2039 [published Online First: 2016/03/05]
5. Liu CW, Attar KH, Gall A, et al. The relationship between bladder management and health-related quality of life in patients with spinal cord injury in the UK. *Spinal cord* 2010;48(4):319-24. doi: 10.1038/sc.2009.132 [published Online First: 2009/10/21]
6. Crescenze IM, Myers JB, Lenherr SM, et al. Predictors of low urinary quality of life in spinal cord injury patients on clean intermittent catheterization. *Neurourology and urodynamics* 2019 doi: 10.1002/nau.23983 [published Online First: 2019/03/27]
7. Myers JB, Lenherr SM, Stoffel JT, et al. Patient Reported Bladder Related Symptoms and Quality of Life after Spinal Cord Injury with Different Bladder Management Strategies. *The Journal of urology* 2019:101097JU0000000000000270. doi: 10.1097/ju.0000000000000270 [published Online First: 2019/04/09]
8. Panicker JN, Fowler CJ, Kessler TM. Lower urinary tract dysfunction in the neurological patient: clinical assessment and management. *The Lancet Neurology* 2015;14(7):720-32. doi: 10.1016/s1474-4422(15)00070-8 [published Online First: 2015/06/13]
9. de Groat WC, Yoshimura N. Mechanisms underlying the recovery of lower urinary tract function following spinal cord injury. *Progress in brain research* 2006;152:59-84. doi: 10.1016/s0079-6123(05)52005-3 [published Online First: 2005/10/04]
10. Blok BF, Holstege G. The pontine micturition center in rat receives direct lumbosacral input. An ultrastructural study. *Neuroscience letters* 2000;282(1-2):29-32. doi: 10.1016/s0304-3940(00)00833-8 [published Online First: 2000/03/14]
11. Shingleton WB, Bodner DR. The development of urologic complications in relationship to bladder pressure in spinal cord injured patients. *The Journal of the American Paraplegia Society* 1993;16(1):14-7. doi: 10.1080/01952307.1993.11735878 [published Online First: 1993/01/01]
12. Lawrenson R, Wyndaele JJ, Vlachonikolis I, et al. Renal failure in patients with neurogenic lower urinary tract dysfunction. *Neuroepidemiology* 2001;20(2):138-43. doi: 10.1159/000054774 [published Online First: 2001/05/19]
13. Groen J, Pannek J, Castro Diaz D, et al. Summary of European Association of Urology (EAU) Guidelines on Neuro-Urology. *European urology* 2016;69(2):324-33. doi: 10.1016/j.eururo.2015.07.071 [published Online First: 2015/08/26]
14. Welk B, Schneider MP, Thavaseelan J, et al. Early urological care of patients with spinal cord injury. *World journal of urology* 2018;36(10):1537-44. doi: 10.1007/s00345-018-2367-7 [published Online First: 2018/06/28]
15. Wollner J, Kessler TM. Botulinum toxin injections into the detrusor. *BJU international* 2011;108(9):1528-37. doi: 10.1111/j.1464-410X.2011.10675.x [published Online First: 2011/10/26]
16. Mangera A, Apostolidis A, Andersson KE, et al. An updated systematic review and statistical comparison of standardised mean outcomes for the use of botulinum toxin in the management of lower urinary tract disorders. *European urology*

For peer review only - http://bmjopen.bmj.com/site/about/guidelines.xhtml

2014;65(5):981-90. doi: 10.1016/j.eururo.2013.10.033 [published Online First: 2013/11/19]
17. Leitner L, Guggenbuhl-Roy S, Knupfer SC, et al. More Than 15 Years of Experience with Intradetrusor OnabotulinumtoxinA Injections for Treating Refractory Neurogenic Detrusor Overactivity: Lessons to Be Learned. *European urology* 2016;70(3):522-8. doi: 10.1016/j.eururo.2016.03.052 [published Online First: 2016/04/24]
18. Hoen L, Ecclestone H, Blok BFM, et al. Long-term effectiveness and complication rates of bladder augmentation in patients with neurogenic bladder dysfunction: A systematic review. *Neurourology and urodynamics* 2017;36(7):1685-702. doi: 10.1002/nau.23205 [published Online First: 2017/02/09]
19. Phe V, Boissier R, Blok BFM, et al. Continent catheterizable tubes/stomas in adult neuro-urological patients: A systematic review. *Neurourology and urodynamics* 2017;36(7):1711-22. doi: 10.1002/nau.23213 [published Online First: 2017/02/01]
20. Sievert KD, Amend B, Gakis G, et al. Early sacral neuromodulation prevents urinary incontinence after complete spinal cord injury. *Annals of neurology* 2010;67(1):74-84. doi: 10.1002/ana.21814 [published Online First: 2010/02/27]
21. Shi P, Zhao X, Wang J, et al. Effects of acute sacral neuromodulation on bladder reflex in complete spinal cord injury rats. *Neuromodulation : journal of the International Neuromodulation Society* 2013;16(6):583-9; discussion 89. doi: 10.1111/j.1525-1403.2012.00528.x [published Online First: 2012/11/07]
22. Li P, Liao L, Chen G, et al. Early low-frequency stimulation of the pudendal nerve can inhibit detrusor overactivity and delay progress of bladder fibrosis in dogs with spinal cord injuries. *Spinal cord* 2013;51(9):668-72. doi: 10.1038/sc.2013.60 [published Online First: 2013/06/26]
23. Schneider MP, Gross T, Bachmann LM, et al. Tibial nerve stimulation for treating neurogenic lower urinary tract dysfunction: a systematic review. *European urology* 2015;68(5):859-67. doi: 10.1016/j.eururo.2015.07.001 [published Online First: 2015/07/22]
24. Gross T, Schneider MP, Bachmann LM, et al. Transcutaneous electrical nerve stimulation for treating neurogenic lower urinary tract dysfunction: a systematic review. *European urology* 2016;69(6):1102-11. doi: 10.1016/j.eururo.2016.01.010 [published Online First: 2016/02/03]
25. Finazzi-Agro E, Petta F, Sciobica F, et al. Percutaneous tibial nerve stimulation effects on detrusor overactivity incontinence are not due to a placebo effect: a randomized, double-blind, placebo controlled trial. *The Journal of urology* 2010;184(5):2001-6. doi: 10.1016/j.juro.2010.06.113 [published Online First: 2010/09/21]
26. Gad PN, Kreydin E, Zhong H, et al. Non-invasive Neuromodulation of Spinal Cord Restores Lower Urinary Tract Function After Paralysis. *Frontiers in neuroscience* 2018;12:432. doi: 10.3389/fnins.2018.00432 [published Online First: 2018/07/17]
27. Peters KM, Carrico DJ, Perez-Marrero RA, et al. Randomized trial of percutaneous tibial nerve stimulation versus Sham efficacy in the treatment of overactive bladder syndrome: results from the SUmiT trial. *The Journal of urology* 2010;183(4):1438-43. doi: 10.1016/j.juro.2009.12.036 [published Online First: 2010/02/23]
28. Finazzi-Agro E, Rocchi C, Pachatz C, et al. Percutaneous tibial nerve stimulation produces effects on brain activity: study on the modifications of the long latency somatosensory evoked potentials. *Neurourology and urodynamics* 2009;28(4):320-4. doi: 10.1002/nau.20651 [published Online First: 2008/12/19]
29. Stampas A, Korupolu R, Zhu L, et al. Safety, Feasibility, and Efficacy of Transcutaneous Tibial Nerve Stimulation in Acute Spinal Cord Injury Neurogenic Bladder: A Randomized Control Pilot Trial. *Neuromodulation : journal of the International Neuromodulation Society* 2019;22(6):716-22. doi: 10.1111/ner.12855 [published Online First: 2018/10/05]
30. Stampas A, Gustafson K, Korupolu R, et al. Bladder Neuromodulation in Acute Spinal Cord Injury via Transcutaneous Tibial Nerve Stimulation: Cystometrogram and Autonomic Nervous System Evidence From a Randomized Control Pilot Trial. *Frontiers in neuroscience* 2019;13:119-19. doi: 10.3389/fnins.2019.00119

For peer review only - http://bmjopen.bmj.com/site/about/guidelines.xhtml

31. Chan AW, Tetzlaff JM, Altman DG, et al. SPIRIT 2013: new guidance for content of clinical trial protocols. *Lancet (London, England)* 2013;381(9861):91-2. doi: 10.1016/s0140-6736(12)62160-6 [published Online First: 2013/01/12]
32. Liechti MD, van der Lely S, Stalder SA, et al. Update from TASCI, a Nationwide, Randomized, Sham-controlled, Double-blind Clinical Trial on Transcutaneous Tibial Nerve Stimulation in Patients with Acute Spinal Cord Injury to Prevent Neurogenic Detrusor Overactivity. *European urology focus* 2019 doi: 10.1016/j.euf.2019.09.019 [published Online First: 2019/10/12]
33. Post MW, Brinkhof MW, von Elm E, et al. Design of the Swiss Spinal Cord Injury Cohort Study. *American journal of physical medicine & rehabilitation / Association of Academic Physiatrists* 2011;90(11 Suppl 2):S5-16. doi: 10.1097/PHM.0b013e318230fd41 [published Online First: 2011/11/10]
34. Association ASI. International standards for neurological classification of spinal cord injury (ISNCSCI). Richmond, VA, USA: American Spinal Injury Association, 2019.
35. Pavese C, Schneider MP, Schubert M, et al. Prediction of Bladder Outcomes after Traumatic Spinal Cord Injury: A Longitudinal Cohort Study. *PLoS medicine* 2016;13(6):e1002041. doi: 10.1371/journal.pmed.1002041 [published Online First: 2016/06/22]
36. Harris PA, Taylor R, Minor BL, et al. The REDCap consortium: Building an international community of software platform partners. *Journal of biomedical informatics* 2019;95:103208. doi: 10.1016/j.jbi.2019.103208 [published Online First: 2019/05/13]
37. Harris PA, Taylor R, Thielke R, et al. Research electronic data capture (REDCap)--a metadata-driven methodology and workflow process for providing translational research informatics support. *Journal of biomedical informatics* 2009;42(2):377-81. doi: 10.1016/j.jbi.2008.08.010 [published Online First: 2008/10/22]
38. Sterne JA, White IR, Carlin JB, et al. Multiple imputation for missing data in epidemiological and clinical research: potential and pitfalls. *BMJ (Clinical research ed)* 2009;338:b2393. doi: 10.1136/bmj.b2393 [published Online First: 2009/07/01]
39. Carpenter J, Kenward, M.G. Multiple Imputatation and its Application: Wiley, Statistics in Practice 2013.

For peer review only

For peer review only - http://bmjopen.bmj.com/site/about/guidelines.xhtml

FIGURE LEGENDS

Figure 1: Trial flowchart. Safety data will be recorded on a continuous basis the entire time the participant is enrolled in the study.

* Additional clinical assessments target urological and bowel function and symptoms, neurological structure and function, sexual function, spasticity, functional independence, quality of life, and biochemical and molecular biomarkers in the urine, blood, stool, and in bladder tissue (additional consent required for bladder biopsy).

** Histomorphology is based on bladder biopsy which requires additional consent.

DO, detrusor overactivity; SCI, spinal cord injury; TTNS, transcutaneous tibial nerve stimulation.

Figure 2. TTNS Intervention. Pictures of the TTNS device, cables and electrode placements. A) The TTNS device has two channels that can be adjusted independently. Electrodes are placed in a specified, standardised order (1-4). B) Lateral view of the foot, showing the electrode placement on the dorsal bone and plantar fat pad of the 5th metatarsophalangeal joint. C) Medial view of the foot, electrodes are placed 4-5 cm proximal and posterior to the medial malleolus, as well as in the longitudinal arch of the foot, distal to, and in line with, the medial malleolus. TTNS, transcutaneous tibial nerve stimulation.

For peer review only - http://bmjopen.bmj.com/site/about/guidelines.xhtml

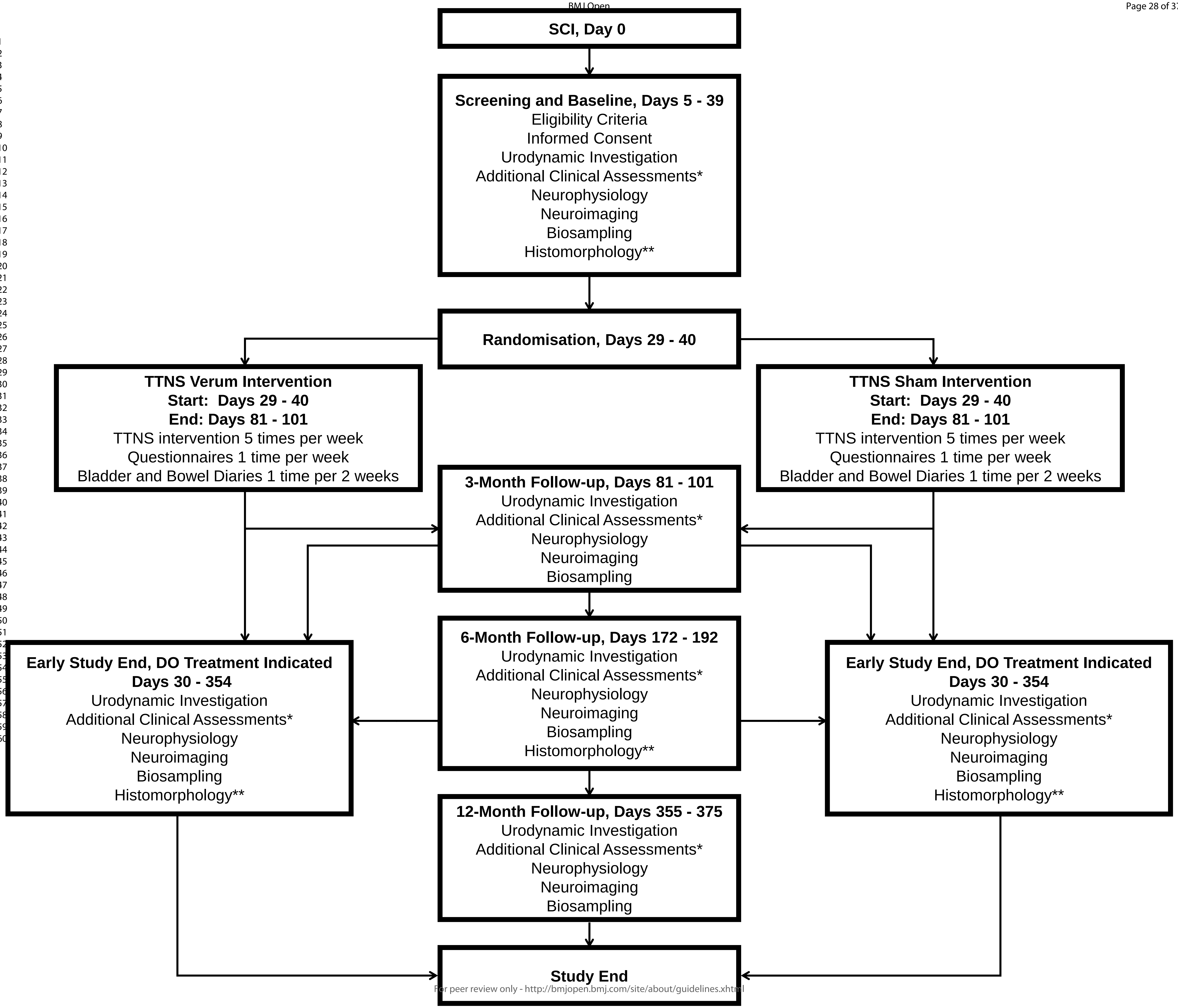
SCI, Day 0
Screening and Baseline, Days 5 - 39
Eligibility Criteria
Informed Consent
Urodynamic Investigation
Additional Clinical Assessments*
Neurophysiology
Neuroimaging
Biosampling
Histomorphology**
Randomisation, Days 29 - 40
TTNS Verum Intervention
Start: Days 29 - 40
End: Days 81 - 101
TTNS intervention 5 times per week
Questionnaires 1 time per week
Bladder and Bowel Diaries 1 time per 2 weeks
TTNS Sham Intervention
Start: Days 29 - 40
End: Days 81 - 101
TTNS intervention 5 times per week
Questionnaires 1 time per week
Bladder and Bowel Diaries 1 time per 2 weeks
3-Month Follow-up, Days 81 - 101
Urodynamic Investigation
Additional Clinical Assessments*
Neurophysiology
Neuroimaging
Biosampling
6-Month Follow-up, Days 172 - 192
Urodynamic Investigation
Additional Clinical Assessments*
Neurophysiology
Neuroimaging
Biosampling
Histomorphology**
Early Study End, DO Treatment Indicated
Days 30 - 354
Urodynamic Investigation
Additional Clinical Assessments*
Neurophysiology
Neuroimaging
Biosampling
Histomorphology**
Early Study End, DO Treatment Indicated
Days 30 - 354
Urodynamic Investigation
Additional Clinical Assessments*
Neurophysiology
Neuroimaging
Biosampling
Histomorphology**
12-Month Follow-up, Days 355 - 375
Urodynamic Investigation
Additional Clinical Assessments*
Neurophysiology
Neuroimaging
Biosampling
Study End

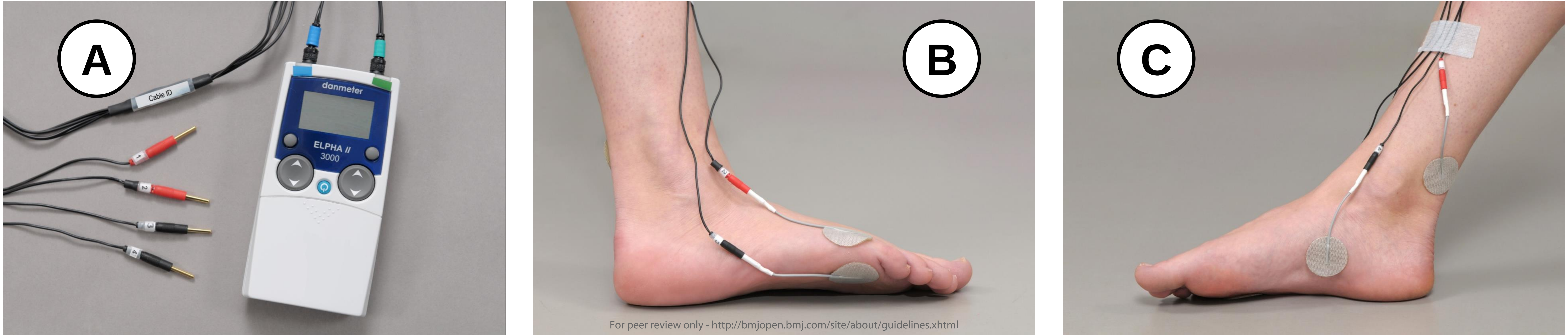

For peer review only - http://bmjopen.bmj.com/site/about/guidelines.xhtml

STANDARD PROTOCOL ITEMS: RECOMMENDATIONS FOR INTERVENTIONAL TRIALS

Supplement 1 – SPIRIT checklist for the TASCI (Transcutaneous Tibial Nerve Stimulation in Acute Spinal Cord Injury) Trial Protocol

SPIRIT 2013 Checklist: Recommended items to address in a clinical trial protocol and related documents*

Section/item	Item No	Description
**Administrative information**		
Title	1	Descriptive title identifying the study design, population, interventions, and, if applicable, trial acronym ✔ **Title, page 1**
Trial registration	2a	Trial identifier and registry name. If not yet registered, name of intended registry ✔ **Abstract, page 4**
	2b	All items from the World Health Organization Trial Registration Data Set – ✔ **Throughout the text, pages 1-21, and publicly accessible information on clinicaltrials.gov, NCT03965299**
Protocol version	3	Date and version identifier ✔ **Trial Status, page 20**
Funding	4	Sources and types of financial, material, and other support - ✔ **Funding Statement, page 21**
Roles and responsibilities	5a	Names, affiliations, and roles of protocol contributors ✔ **Title Page, pages 1-2; Contributor Statement, pages 20-21**
	5b	Name and contact information for the trial sponsor ✔ **Title Page (Corresponding Author), page 2**
	5c	Role of study sponsor and funders, if any, in study design; collection, management, analysis, and interpretation of data; writing of the report; and the decision to submit the report for publication, including whether they will have ultimate authority over any of these activities ✔ **Contributor Statement, Funding Statement , pages 20, 21**
	5d	Composition, roles, and responsibilities of the coordinating centre, steering committee, endpoint adjudication committee, data management team, and other individuals or groups overseeing the trial, if applicable (see Item 21a for data monitoring committee) ✔ **Dissemination, pages 18-19; Contributor Statement, pages 20-21**

For peer review only

Introduction

Background and rationale	6a	Description of research question and justification for undertaking the trial, including summary of relevant studies (published and unpublished) examining benefits and harms for each intervention ✔ **Introduction, pages 5-7**
	6b	Explanation for choice of comparators – ✔ **Introduction, page 6; Methods: Randomisation and blinding, Intervention, page 10**
Objectives	7	Specific objectives or hypotheses ✔ **Introduction, page 6-7**
Trial design	8	Description of trial design including type of trial (eg, parallel group, crossover, factorial, single group), allocation ratio, and framework (eg, superiority, equivalence, noninferiority, exploratory) ✔ **Methods: Study design and setting, page 7; Randomisation and blinding, page 10**

Methods: Participants, interventions, and outcomes

Study setting	9	Description of study settings (eg, community clinic, academic hospital) and list of countries where data will be collected. Reference to where list of study sites can be obtained ✔ **Methods: Study design and setting, page 7**
Eligibility criteria	10	Inclusion and exclusion criteria for participants. If applicable, eligibility criteria for study centres and individuals who will perform the interventions (eg, surgeons, psychotherapists) ✔ **Methods: Participants, page 8; Box 1: Eligibility criteria, page 9**
Interventions	11a	Interventions for each group with sufficient detail to allow replication, including how and when they will be administered ✔ **Methods: Intervention, pages 10-12. Please note: some details have been left vague in order to prevent participants from unblinding themselves with a Google search.**
	11b	Criteria for discontinuing or modifying allocated interventions for a given trial participant (eg, drug dose change in response to harms, participant request, or improving/worsening disease) ✔ **Methods: Intervention, page 12**
	11c	Strategies to improve adherence to intervention protocols, and any procedures for monitoring adherence (eg, drug tablet return, laboratory tests) ✔ **Methods: Intervention, pages 11-12**
	11d	Relevant concomitant care and interventions that are permitted or prohibited during the trial ✔ **Methods: Intervention, pages 11-12, Box 1 Eligibility criteria, page 9**

For peer review only - http://bmjopen.bmj.com/site/about/guidelines.xhtml

Outcomes	12	Primary, secondary, and other outcomes, including the specific measurement variable (eg, systolic blood pressure), analysis metric (eg, change from baseline, final value, time to event), method of aggregation (eg, median, proportion), and time point for each outcome. Explanation of the clinical relevance of chosen efficacy and harm outcomes is strongly recommended ✔ **Methods: Outcomes, pages 12-13; Table 1. Secondary outcomes and outcome measures, page 14; Supplement 2. Secondary outcome parameters**
Participant timeline	13	Time schedule of enrolment, interventions (including any run-ins and washouts), assessments, and visits for participants. A schematic diagram is highly recommended (see Figure) ✔ **Methods: Study design and setting, page 7, Intervention, pages 11-12, Figure 1. Trial Flowchart**
Sample size	14	Estimated number of participants needed to achieve study objectives and how it was determined, including clinical and statistical assumptions supporting any sample size calculations ✔ **Methods: Sample size, page 15**
Recruitment	15	Strategies for achieving adequate participant enrolment to reach target sample size **– Not explicitly mentioned, all patients with a spinal cord injury (SCI) coming through all Swiss specialized SCI centres will be screened for eligibility.**

Methods: Assignment of interventions (for controlled trials)

Allocation:

Sequence generation	16a	Method of generating the allocation sequence (eg, computer-generated random numbers), and list of any factors for stratification. To reduce predictability of a random sequence, details of any planned restriction (eg, blocking) should be provided in a separate document that is unavailable to those who enrol participants or assign interventions ✔ **Methods: Randomisation and blinding, page 10**
Allocation concealment mechanism	16b	Mechanism of implementing the allocation sequence (eg, central telephone; sequentially numbered, opaque, sealed envelopes), describing any steps to conceal the sequence until interventions are assigned ✔ **Methods: Randomisation and blinding, page 10**
Implementation	16c	Who will generate the allocation sequence, who will enrol participants, and who will assign participants to interventions ✔ **Methods: Participants, page 8, Randomisation and blinding, page 10**
Blinding (masking)	17a	Who will be blinded after assignment to interventions (eg, trial participants, care providers, outcome assessors, data analysts), and how ✔ **Methods: Randomisation and blinding, Intervention, pages 10 - 12**

	17b	If blinded, circumstances under which unblinding is permissible, and procedure for revealing a participant's allocated intervention during the trial – **Not explicitly mentioned, we do not foresee any need to unblind participants for the duration of their participation in the trial, as early evidence and clinical experience has indicated that the intervention is safe.**

Methods: Data collection, management, and analysis

Data collection methods	18a	Plans for assessment and collection of outcome, baseline, and other trial data, including any related processes to promote data quality (eg, duplicate measurements, training of assessors) and a description of study instruments (eg, questionnaires, laboratory tests) along with their reliability and validity, if known. Reference to where data collection forms can be found, if not in the protocol ✔ **Methods: Outcomes, pages 12-13; Table 1 Secondary outcomes, page 14; Supplement 2; Data collection and management, pages 15-16; Statistical Analyses, pages 16-17**
	18b	Plans to promote participant retention and complete follow-up, including list of any outcome data to be collected for participants who discontinue or deviate from intervention protocols ✔ **Methods: Intervention, pages 11-12, Outcomes, page 13**
Data management	19	Plans for data entry, coding, security, and storage, including any related processes to promote data quality (eg, double data entry; range checks for data values). Reference to where details of data management procedures can be found, if not in the protocol ✔ **Methods: Data management, pages 15-16**
Statistical methods	20a	Statistical methods for analysing primary and secondary outcomes. Reference to where other details of the statistical analysis plan can be found, if not in the protocol ✔ **Methods: Statistical analyses, pages 16-17**
	20b	Methods for any additional analyses (eg, subgroup and adjusted analyses) ✔ **Methods: Statistical analyses, pages 16-17**
	20c	Definition of analysis population relating to protocol non-adherence (eg, as randomised analysis), and any statistical methods to handle missing data (eg, multiple imputation) ✔ **Methods: Statistical analyses, pages 16-17**

For peer review only - http://bmjopen.bmj.com/site/about/guidelines.xhtml

Methods: Monitoring

Data monitoring	21a	Composition of data monitoring committee (DMC); summary of its role and reporting structure; statement of whether it is independent from the sponsor and competing interests; and reference to where further details about its charter can be found, if not in the protocol. Alternatively, an explanation of why a DMC is not needed **~ There is a monitoring committee, but it is responsible for general trial conduct as well as data concerns. Sections: Data management, Quality assurance and oversight, pages 15-18**
	21b	Description of any interim analyses and stopping guidelines, including who will have access to these interim results and make the final decision to terminate the trial ✔ **Methods: Statistical analyses, page 17**
Harms	22	Plans for collecting, assessing, reporting, and managing solicited and spontaneously reported adverse events and other unintended effects of trial interventions or trial conduct ✔ **Methods: Outcomes, page 13; Table 1, page 14; Supplement 2**
Auditing	23	Frequency and procedures for auditing trial conduct, if any, and whether the process will be independent from investigators and the sponsor ✔ **Methods: Quality assurance and oversight, pages 17-18**

Ethics and dissemination

Research ethics approval	24	Plans for seeking research ethics committee/institutional review board (REC/IRB) approval ✔ **Ethics and Dissemination: Ethics, page 18**
Protocol amendments	25	Plans for communicating important protocol modifications (eg, changes to eligibility criteria, outcomes, analyses) to relevant parties (eg, investigators, REC/IRBs, trial participants, trial registries, journals, regulators) **– Not directly stated: by default in Switzerland all important protocol modifications have to be communicated to the ethics committees, who will pass information along regulators as appropriate. Trial participants receive an update letter, and there are representatives from each investigating centre on the steering committee.**
Consent or assent	26a	Who will obtain informed consent or assent from potential trial participants or authorised surrogates, and how (see Item 32) ✔ **Methods: Participants, page 8**
	26b	Additional consent provisions for collection and use of participant data and biological specimens in ancillary studies, if applicable ✔ **Methods: Outcomes, page 13**

For peer review only - http://bmjopen.bmj.com/site/about/guidelines.xhtml

Confidentiality	27	How personal information about potential and enrolled participants will be collected, shared, and maintained in order to protect confidentiality before, during, and after the trial ✔ **Methods: Data management, pages 15-16; Ethics and dissemination, pages 18-19**
Declaration of interests	28	Financial and other competing interests for principal investigators for the overall trial and each study site ✔ **Competing interests, page 21**
Access to data	29	Statement of who will have access to the final trial dataset, and disclosure of contractual agreements that limit such access for investigators ✔ **Contributor Statement, Dissemination, pages 18-19**
Ancillary and post-trial care	30	Provisions, if any, for ancillary and post-trial care, and for compensation to those who suffer harm from trial participation - ✔ **Methods: Intervention (ancillary care), pages 11-12. The need to compensate for harm is not foreseen, previous studies and clinical experience in other patient groups have not raised any safety concerns**
Dissemination policy	31a	Plans for investigators and sponsor to communicate trial results to participants, healthcare professionals, the public, and other relevant groups (eg, via pub lication, reporting in results databases, or other data sharing arrangements), including any publication restrictions ✔ **Ethics and Dissemination: Patient and public involvement, Dissemination, pages 18-19**
	31b	Authorship eligibility guidelines and any intended use of professional writers ✔ **Ethics and Dissemination: Dissemination, pages 18-19**
	31c	Plans, if any, for granting public access to the full protocol, participant-level dataset, and statistical code ✔ **Ethics and Dissemination: Dissemination, pages 18-19**
**Appendices**		
Informed consent materials	32	Model consent form and other related documentation given to participants and authorised surrogates **NA – Current versions are in German and French**
Biological spcimens	33	Plans for collection, laboratory evaluation, and storage of biological specimens for genetic or molecular analysis in the current trial and for future use in ancillary studies, if applicable ✔ **Methods: Outcomes, pages 12-13; Figure 1; Table 1, page 14; Supplement 2**

*It is strongly recommended that this checklist be read in conjunction with the SPIRIT 2013 Explanation & Elaboration for important clarification on the items. Amendments to the protocol should be tracked and dated. The SPIRIT checklist is copyrighted by the SPIRIT Group under the Creative Commons "Attribution-NonCommercial-NoDerivs 3.0 Unported" license.

For peer review only - http://bmjopen.bmj.com/site/about/guidelines.xhtml

Supplement 2. Secondary outcome parameters. Assessment periods are referenced to the date of spinal cord injury (SCI) diagnosis and include: screening and baseline (S&B) (days 5-39), 3-month follow-up (day 91 +/- 10), 6-month follow-up (day 182 +/-10), 12-month follow-up (day 365 +/-10). Additionally, some surveys are administered weekly during the TTNS treatment period (TTNS: weekly), and the bowel and bladder diaries are administered once every two weeks during the treatment period (TTNS: bi-weekly).

Outcome and Measurement Parameter (analysis metric)	Assessment Periods
**Occurrence of neurogenic detrusor overactivity (DO) jeopardizing the upper urinary tract over a 1-year time course assessed by: urodynamic investigation and clinical assessment**	
DO amplitude ≥40 cmH_2O, or initiation of DO treatment with antimuscarinics and/or intradetrusor onabotulinumtoxinA injections (time-to-event analysis)	S&B, 3, 6, 12 months
**Differential changes in lower urinary tract (LUT) function, assessed by urodynamic investigation**	
Maximum cystometric capacity (mL)	S&B, 3, 6, 12 months
Compliance (mL/cmH_2O)	S&B, 3, 6, 12 months
DO (present or absent), if present:	S&B, 3, 6, 12 months
Bladder volume at first DO (mL)	S&B, 3, 6, 12 months
Maximum DO amplitude (cmH_2O)	S&B, 3, 6, 12 months
Detrusor leak-point pressure (cmH_2O)	S&B, 3, 6, 12 months
Maximum detrusor pressure during storage phase (cmH_2O)	S&B, 3, 6, 12 months
Maximum detrusor pressure during voiding phase (cmH_2O)	S&B, 3, 6, 12 months
Detrusor pressure at maximum flow rate (cmH_2O)	S&B, 3, 6, 12 months
Maximum flow rate (mL/s)	S&B, 3, 6, 12 months
Voided volume (mL)	S&B, 3, 6, 12 months
Post void residual (mL)	S&B, 3, 6, 12 months
Pelvic floor electromyography (EMG) (normal/pathological)	S&B, 3, 6, 12 months
Videography (vesico-uretero-renal reflux, present/absent)	S&B, 3, 6, 12 months
Free flow curve pattern	S&B, 3, 6, 12 months
**Differential changes in lower spinal cord function, assessed by neuro-urological physical examination**	
Voluntary sphincter contraction (present/absent; if present, weak/strong)	S&B, 3, 6, 12 months
Resting anal tonus (weak/strong/spastic)	S&B, 3, 6, 12 months
Bulbocavernosus reflex(present/absent)	S&B, 3, 6, 12 months
Anal reflex (present/absent)	S&B, 3, 6, 12 months
Sensory testing S2-S5 (algesia, aesthesia: present/absent, even between sides, type of sensation)	S&B, 3, 6, 12 months
**Differential changes in LUT symptoms, assessed by bladder diary, International Prostate Symptom Score (IPSS), Urinary Symptom Profile (USP)**	
Number of voids per 24 hours	S&B, 3, 6, 12 months, TTNS: bi-weekly
Number of day-time voids	S&B, 3, 6, 12 months, TTNS: bi-weekly
Number of night-time voids	S&B, 3, 6, 12 months, TTNS: bi-weekly
Number of intermittent catheterizations	S&B, 3, 6, 12 months, TTNS: bi-weekly
Number of leakages per 24 hours	S&B, 3, 6, 12 months, TTNS: bi-weekly
Number of used pads per 24 hours	S&B, 3, 6, 12 months, TTNS: bi-weekly
Mean post void residual (mL)	S&B, 3, 6, 12 months, TTNS: bi-weekly
Mean voided volume (mL)	S&B, 3, 6, 12 months, TTNS: bi-weekly
Pain level (scale, 0-10)	S&B, 3, 6, 12 months, TTNS: bi-weekly
Level of urgency (scale, 0-4)	S&B, 3, 6, 12 months, TTNS: bi-weekly
IPSS Symptom Score (sum score, 0-35)	S&B, 3, 6, 12 months, TTNS: weekly
USP Stress urinary incontinence score (sum score, 0-9)	S&B, 3, 6, 12 months, TTNS: weekly
USP Overactive bladder score (sum score, 0-21)	S&B, 3, 6, 12 months, TTNS: weekly
USP Low stream score (sum score, 0-9)	S&B, 3, 6, 12 months, TTNS: weekly

For peer review only

**Differential changes in quality of life, assessed by IPSS, Qualiveen**	
IPSS Quality of life score (scale, 0-6)	S&B, 3, 6, 12 months, TTNS: weekly
Qualiveen total score (mean score, 0-4)	S&B, 3, 6, 12 months, TTNS: weekly
Qualiveen Limitations / Inconvenience subscale (mean score, 0-4)	S&B, 3, 6, 12 months, TTNS: weekly
Qualiveen Constraints / Restrictions subscale (mean score, 0-4)	S&B, 3, 6, 12 months, TTNS: weekly
Qualiveen Fears subscale (mean score, 0-4)	S&B, 3, 6, 12 months, TTNS: weekly
Qualiveen Feelings / Impact on Daily Life subscale (mean score, 0-4)	S&B, 3, 6, 12 months, TTNS: weekly
**Differential changes in sexual function, assessed by clinical assessment, FSFI (females), IIEF (males)**	
Psychogenic erection, M (present / reduced / absent / unknown / not applicable)	S&B, 3, 6, 12 months
Reflex erection, M (present / reduced / absent / unknown / not applicable)	S&B, 3, 6, 12 months
Ejaculation, M (present / reduced / absent / unknown / not applicable)	S&B, 3, 6, 12 months
Orgasmic function, M, F (present / reduced / absent / unknown / not applicable)	S&B, 3, 6, 12 months
Psychogenic genital arousal, F (present / reduced / absent / unknown / not applicable)	S&B, 3, 6, 12 months
Reflex genital arousal, F (present / reduced / absent / unknown / not applicable)	S&B, 3, 6, 12 months
Menstruation, F (present / reduced / absent / unknown / not applicable)	S&B, 3, 6, 12 months
FSFI, F total score (sum score, 0-36)	S&B, 3, 6, 12 months
FSFI, F Desire domain (sum score, 0-6)	S&B, 3, 6, 12 months
FSFI, F Arousal domain (sum score, 0-6)	S&B, 3, 6, 12 months
FSFI, F Lubrication domain (sum score, 0-6)	S&B, 3, 6, 12 months
FSFI, F Orgasm domain (sum score, 0-6)	S&B, 3, 6, 12 months
FSFI, F Satisfaction domain (sum score, 0-6)	S&B, 3, 6, 12 months
FSFI, F Pain domain (sum score, 0-6)	S&B, 3, 6, 12 months
IIEF, M total score (sum score, 5-75)	S&B, 3, 6, 12 months
IIEF, M Erectile Function domain (sum score, 1-30)	S&B, 3, 6, 12 months
IIEF, M Orgasmic Function domain (sum score, 0-10)	S&B, 3, 6, 12 months
IIEF, M Sexual Desire domain (sum score, 2-10)	S&B, 3, 6, 12 months
IIEF, M Intercourse Satisfaction domain (sum score, 0-15)	S&B, 3, 6, 12 months
IIEF, M Overall Satisfaction domain (sum score, 2-10)	S&B, 3, 6, 12 months
**Differential changes in bowel symptoms, assessed by NBD, bowel diary**	
NBD Score (version from ISCoS Bowel Function Dataset) (sum score, 0-47)	S&B, 3, 6, 12 months, TTNS: weekly
Number of defecations per 24h	S&B, 3, 6, 12 months, TTNS: bi-weekly
Number of urgency episodes per 24h	S&B, 3, 6, 12 months, TTNS: bi-weekly
Quantity of stool per defecation (Small/Medium/Large)	S&B, 3, 6, 12 months, TTNS: bi-weekly
Feeling of incomplete evacuation per 24h	S&B, 3, 6, 12 months, TTNS: bi-weekly
Number of accidental bowel leakages per 24h	S&B, 3, 6, 12 months, TTNS: bi-weekly
Quantity per bowel leakage (Small/Medium/Large)	S&B, 3, 6, 12 months, TTNS: bi-weekly
Stool consistency score (Bristol Stool Scale)	S&B, 3, 6, 12 months, TTNS: bi-weekly
Pain related to defecation (score)	S&B, 3, 6, 12 months, TTNS: bi-weekly
Medications for bowel	S&B, 3, 6, 12 months, TTNS: bi-weekly
**Differential changes in bowel function and pelvic floor function, assessed by rectal sensitivity examination, anorectal manometry, and barostat assessment**	
Initial sensation (mL)	S&B, 3, 6, 12 months
Urgency to defecate (mL)	S&B, 3, 6, 12 months
Maximum tolerated volume (mL)	S&B, 3, 6, 12 months
Basal internal anal sphincter pressure (mmHg)	S&B, 3, 6, 12 months
Squeeze external anal sphincter pressure (mmHg)	S&B, 3, 6, 12 months
Relaxation internal anal sphincter pressure during defecation (%, mmHg)	S&B, 3, 6, 12 months
Intraabdominal pressure during defecation (mmHg)	S&B, 3, 6, 12 months

For peer review only

Defecatory disorder (Rao Classification I-IV)	S&B, 3, 6, 12 months
Rectal capacity (mL)	S&B, 3, 6, 12 months
Rectal compliance (mL/mmHg)	S&B, 3, 6, 12 months
**Differential changes in motor and sensory function, assessed by the International Standards for Neurological Classification of Spinal Cord Injury (ISNCSCI) protocol**	
American Spinal Injury Association (ASIA) Impairment scale (AIS) Grade (A,B,C,D,E)	S&B, 3, 6, 12 months
ISNCSCI upper extremity motor score (UEMS) (score, 0-50)	S&B, 3, 6, 12 months
ISNCSCI lower extremity motor score (LEMS) (score, 0-50)	S&B, 3, 6, 12 months
ISNCSCI light touch (LT) score (score, 0-112)	S&B, 3, 6, 12 months
ISNCSCI pin prick (PP) score (score, 0-112)	S&B, 3, 6, 12 months
**Differential changes in functional independence assessed by the Spinal Independence Measure (SCIM) III**	
SCIM III Score (sum score, 0-100)	S&B, 3, 6, 12 months
**Differential changes in spasticity, assessed by Spinal Cord Injury Spasticity Evaluation Tool (SCI-SET), Modified Ashworth Scale (MAS)**	
SCI-SET Score (sum score, -105 to +105; mean score: -3 to +3, number of positive responses, number of negative responses)	S&B, 3, 6, 12 months, TTNS: weekly
Muscle tone of knee flexors and extensors (scale, 0-4)	S&B, 3, 6, 12 months
Muscle tone of elbow flexors and extensors (scale, 0-4)	S&B, 3, 6, 12 months
**Differential changes in brain and spinal cord function as well as cortico-spinal integrity, assessed using neurophysiological and neuroimaging methods: nerve conduction studies (NCS), EMG, electroencephalography (EEG), transcranial magnetic stimulation (TMS)**	
Latencies and amplitudes of F-waves to electrical stimulation	S&B, 3, 6, 12 months
Compound muscle action potentials (cMAP)	S&B, 3, 6, 12 months
Current perception thresholds (CPTs)	S&B, 3, 6, 12 months
Sensory evoked potentials (SEPs): latencies and amplitudes in response to electrical nerve stimulation	S&B, 3, 6, 12 months
Long latency sensory evoked potentials (LL-SEPs): latencies and amplitudes, in response to electrical nerve stimulation	S&B, 3, 6, 12 months
Sympathetic skin response (SSR): latencies and amplitudes	S&B, 3, 6, 12 months
Frequency and coherence measures of surface EMG and EEG	S&B, 3, 6, 12 months
Lower urinary tract sensory evoked potentials (LUTSEPs): latencies and amplitudes in response to electrical LUT stimulation	S&B, 3, 6, 12 months
Changes in white and grey matter area in the lumbosacral enlargement (LSE) and their relation to clinical outcomes	S&B, 3, 6, 12 months
Changes in white and grey matter area in upper cervical spinal cord (at C2/C3) and their relation to clinical outcomes	S&B, 3, 6, 12 months
Changes in white and grey matter volume of the conus medullaris (CM) and their relation to clinical outcomes	S&B, 3, 6, 12 months
Changes in grey and white matter volume in the brain and their relation to clinical outcomes	S&B, 3, 6, 12 months
Changes in fractional anisotropy (FA) in the brain and spinal cord and their relation to clinical outcomes	S&B, 3, 6, 12 months
Changes in diffusivity in the brain and spinal cord and their relation to clinical outcomes	S&B, 3, 6, 12 months
Changes in brain and spinal cord tissue microstructure and their relation to clinical outcomes	S&B, 3, 6, 12 months
Changes in brain function and connectivity during rest and electrical stimulation of the tibial nerve and their relation to clinical outcomes	S&B, 3, 6, 12 months
**Differential changes in biochemical and molecular biomarkers in urine, blood, stool, and bladder tissue, assessed by histomorphology, enzyme-linked immunosorbent assay (ELISA), western blot, reverse transcription polymerase chain reaction (RT-PCR), microbiome analysis**	
Biomarkers reflecting oxidative stress (presence/absence and quantities of peptides, proteins, antibodies, and small molecules)	S&B, 3, 6, 12 months
Expression profile of messenger RNA (mRNA) and microRNA (miRNA) in urine and blood as well as their relation to clinical outcomes	S&B, 3, 6, 12 months
Changes in inflammatory markers in bladder tissue, blood, and urine, as well as their relation to clinical outcomes	S&B, 3, 6, 12 months
Changes in levels of neurotransmitters (neurotrophins) in blood, and urine, as well as their relation to clinical outcomes	S&B, 3, 6, 12 months
Changes in the composition of urinary and stool microbiomes (analysed by sequencing and microbial culturing) and their relation to clinical outcomes	S&B, 3, 6, 12 months
Changes in the density / quality of afferent / efferent innervation of the bladder tissue and their relation to clinical outcomes	S&B, 6 months

For peer review only

**Safety of TTNS, assessed by clinical record check, patient interview, health diary (confirmed by clinician)**	
Urinary tract infection (UTI) (number of occurrences and intensity/severity)	Any time
Infection other than UTI (number of occurrences and intensity/severity)	Any time
Skin irritation at the electrode site (number of occurrences and intensity/severity)	Any time
Irritation-related symptoms (e.g. dysuria, hematuria) with a temporal link to a LUT intervention (i.e. immediately following the intervention) (number of occurrences and intensity/severity)	Any time
Neuro-urological deterioration (number of occurrences and intensity/severity)	Any time
Pressure ulcer (location, number of occurrences and intensity/severity)	Any time
Neurological deterioration (number of occurrences and intensity/severity)*	Any time
Increase in pain (number of occurrences and intensity/severity)*	Any time
Increase in spasticity (number of occurrences and intensity/severity)*	Any time
Deep vein thrombosis / pulmonary embolism (number of occurrences and intensity/severity)	Any time
Autonomic dysreflexia (number of occurrences (suspected and verified) and intensity/severity)	Any time
Transfer/admittance to an acute care facility (number of occurrences and intensity/severity)*	Any time

*only events that qualify as adverse events and serious adverse events are recorded
cMAP compound muscle action potentials; CPTs, current perception thresholds; DO, detrusor overactivity; ELISA, enzyme-linked immunosorbent assay; EEG, electroencephalography; EMG, electromyography; F, female; FSFI, Female Sexual Function Index; IIEF, International Index of Erectile Dysfunction; IPSS, International Prostate Symptom Score; ISNCSCI, the International Standards for Neurological Classification of Spinal Cord Injury; LL-SEP, long latency sensory evoked potential; LUT, lower urinary tract; LUTSEP lower urinary tract sensory evoked potential; M, male; MAS, Modified Ashworth Scale; miRNA, microRNA; mRNA, messenger RNA; NBD, neurogenic bowel dysfunction; NCS, nerve conduction studies; RT-PCR, Reverse transcription polymerase chain reaction; S&B, screening and baseline; SCI, spinal cord injury; SCI-SET, Spinal Cord Injury Spasticity Evaluation Tool; SCIM, Spinal Cord Independence Measure; SEP, sensory evoked potentials; SSR, Sympathetic skin response; TMS, transcranial magnetic stimulation; TTNS, transcutaneous tibial nerve stimulation; USP Urinary Symptom Profile; UTI, urinary tract infection

For peer review only

BMJ Open

TASCI: Transcutaneous tibial nerve stimulation in patients with Acute Spinal Cord Injury to prevent neurogenic detrusor overactivity: Protocol for a nationwide, randomised, sham-controlled, double-blind clinical trial

Journal:	*BMJ Open*
Manuscript ID	bmjopen-2020-039164.R1
Article Type:	Protocol
Date Submitted by the Author:	13-May-2020

Complete List of Authors:	Birkhäuser, Veronika; Balgrist University Hospital, University of Zürich, Department of Neuro-Urology<br>Liechti, Martina; Balgrist University Hospital, University of Zürich, Department of Neuro-Urology<br>Anderson, Collene; Swiss Paraplegic Research; University of Lucerne, Department of Health Sciences and Medicine<br>Bachmann, Lucas; Medignition Inc., Research Consultants<br>Baumann, Sarah; Balgrist University Hospital, University of Zürich, Department of Neuro-Urology<br>Baumberger, Michael; Swiss Paraplegic Centre, Spinal Cord and Rehabilitation Medicine<br>Birder, Lori; University of Pittsburgh School of Medicine, Neuro-Urology<br>Botter, Sander; Balgrist Campus AG, Swiss Center for Musculoskeletal Biobanking<br>Büeler, Silvan; Balgrist University Hospital, University of Zürich, Department of Neuro-Urology<br>Cruz, Célia; Universidade do Porto, Instituto de Investigação e Inovação em Saúde, Translational Neuro-urology Group; Universidade do Porto, Faculdade de Medicina, Departemento de Biomedicina, Unidade de Biologia Experimental<br>David, Gergely; Balgrist University Hospital, University of Zürich, Department of Neuro-Urology; Balgrist University Hospital, University of Zürich, Spinal Cord Injury Center<br>Freund, Patrick; Balgrist University Hospital, University of Zürich, Spinal Cord Injury Center<br>Friedl, Susanne; Balgrist University Hospital, University of Zürich, Spinal Cord Injury Center<br>Gross, Oliver; Balgrist University Hospital, University of Zürich, Department of Neuro-Urology<br>Hund-Georgiadis , Margret; REHAB Basel, Clinic of neurorehabilitation and paraplegiology<br>Husmann, Knut; Balgrist Campus AG, Swiss Center for Musculoskeletal Biobanking<br>Jordan, Xavier; Clinique romande de réadaptation, Spinal Cord Injury Department<br>Koschorke, Miriam; Balgrist University Hospital, University of Zürich, Department of Neuro-Urology<br>Leitner, Lorenz; Balgrist University Hospital, University of Zürich,

Department of Neuro-Urology
Luca, Eugenia; Clinique romande de réadaptation, Spinal Cord Injury Department
Mehnert, Ulrich; Balgrist University Hospital, University of Zürich, Department of Neuro-Urology
Möhr, Sandra; REHAB Basel, Clinic of neurorehabilitation and paraplegiology
Mohammadzada, Freschta; Balgrist University Hospital, University of Zürich, Spinal Cord Injury Center
Monastyrskaya, Katia; University of Bern, Urology Research Laboratory, DBMR
Pfender, Nikolai; Balgrist University Hospital, University of Zürich, Spinal Cord Injury Center
Pohl, Daniel; University Hospital Zürich, Department of Gastroenterology and Hepatology
Sadri, Helen; Balgrist University Hospital, University of Zürich, Department of Neuro-Urology
Sartori, Andrea; Balgrist University Hospital, University of Zürich, Department of Neuro-Urology; University of Zürich, Institute for Regenerative Medicine
Schubert, Martin; Balgrist University Hospital, University of Zürich, Spinal Cord Injury Center
Sprengel, Kai; University Hospital Zürich, Department of Trauma
Stalder, Stephanie; Balgrist University Hospital, University of Zürich, Department of Neuro-Urology
Stoyanov, Jivko; Swiss Paraplegic Research; University of Lucerne, Department of Health Sciences and Medicine
Stress, Cornelia; Balgrist University Hospital, University of Zürich, Department of Neuro-Urology
Tatu, Aurora; University Hospital Zürich, Department of Gastroenterology and Hepatology
Tawadros, Cécile; Clinique romande de réadaptation, Spinal Cord Injury Department
van der Lely, Stéphanie; Balgrist University Hospital, University of Zürich, Department of Neuro-Urology
Wöllner, Jens; Swiss Paraplegic Centre, Neuro-Urology
Zubler, Veronika; Balgrist University Hospital, Department of Radiology
Curt, Armin; Balgrist University Hospital, University of Zürich, Spinal Cord Injury Center
Pannek, Jürgen; Swiss Paraplegic Centre, Neuro-Urology; Inselspital University Hospital Bern, Department of Urology
Brinkhof, Martin; Swiss Paraplegic Research; University of Lucerne, Department of Health Sciences and Medicine
Kessler, Thomas; Balgrist University Hospital, University of Zürich, Department of Neuro-Urology

**Primary Subject Heading**:	Urology
Secondary Subject Heading:	Neurology
Keywords:	Neuro-urology < UROLOGY, Urinary incontinences < UROLOGY, Neurological injury < NEUROLOGY, REHABILITATION MEDICINE, Bladder disorders < UROLOGY

SCHOLARONE™
Manuscripts

I, the Submitting Author has the right to grant and does grant on behalf of all authors of the Work (as defined in the below author licence), an exclusive licence and/or a non-exclusive licence for contributions from authors who are: i) UK Crown employees; ii) where BMJ has agreed a CC-BY licence shall apply, and/or iii) in accordance with the terms applicable for US Federal Government officers or employees acting as part of their official duties; on a worldwide, perpetual, irrevocable, royalty-free basis to BMJ Publishing Group Ltd ("BMJ") its licensees and where the relevant Journal is co-owned by BMJ to the co-owners of the Journal, to publish the Work in this journal and any other BMJ products and to exploit all rights, as set out in our licence.

The Submitting Author accepts and understands that any supply made under these terms is made by BMJ to the Submitting Author unless you are acting as an employee on behalf of your employer or a postgraduate student of an affiliated institution which is paying any applicable article publishing charge ("APC") for Open Access articles. Where the Submitting Author wishes to make the Work available on an Open Access basis (and intends to pay the relevant APC), the terms of reuse of such Open Access shall be governed by a Creative Commons licence – details of these licences and which Creative Commons licence will apply to this Work are set out in our licence referred to above.

Other than as permitted in any relevant BMJ Author's Self Archiving Policies, I confirm this Work has not been accepted for publication elsewhere, is not being considered for publication elsewhere and does not duplicate material already published. I confirm all authors consent to publication of this Work and authorise the granting of this licence.

For peer review only

Target Journal: BMJ Open

TASCI: Transcutaneous tibial nerve stimulation in patients with Acute Spinal Cord Injury to prevent neurogenic detrusor overactivity: Protocol for a nationwide, randomised, sham-controlled, double-blind clinical trial

Veronika Birkhäuser[1,a], Martina D. Liechti[1,a], Collene E. Anderson[1,2,3,a], Lucas M. Bachmann[4], Sarah Baumann[1], Michael Baumberger[5], Lori A. Birder[6], Sander M. Botter[7], Silvan Büeler[1], Célia D. Cruz[8,9], Gergely David[1,10], Patrick Freund[10], Susanne Friedl[10], Oliver Gross[1], Margret Hund-Georgiadis[11], Knut Husmann[7], Xavier Jordan[12], Miriam Koschorke[1], Lorenz Leitner[1], Eugenia Luca[12], Ulrich Mehnert[1], Sandra Möhr[11], Freschta Mohammadzada[10], Katia Monastyrskaya[13], Nikolai Pfender[10], Daniel Pohl[14], Helen Sadri[1], Andrea M. Sartori[1,15], Martin Schubert[10], Kai Sprengel[16], Stephanie A. Stalder[1], Jivko Stoyanov[2,3], Cornelia Stress[1], Aurora Tatu[14], Cécile Tawadros[12], Stéphanie van der Lely[1], Jens Wöllner[17], Veronika Zubler[18], Armin Curt[10], Jürgen Pannek[17,19], Martin W.G. Brinkhof[2,3], Thomas M. Kessler[1,#]

Author affiliations

[1] Department of Neuro-Urology, Balgrist University Hospital, University of Zürich, Zürich, Switzerland

[2] Swiss Paraplegic Research, Nottwil, Switzerland

[3] Department of Health Sciences and Medicine, University of Lucerne, Lucerne, Switzerland

[4] Medignition Inc., Research Consultants, Zürich, Switzerland

[5] Spinal Cord and Rehabilitation Medicine, Swiss Paraplegic Centre, Nottwil, Switzerland

[6] Neuro-Urology, University of Pittsburgh School of Medicine, Pittsburgh, PA, USA

[7] Swiss Center for Musculoskeletal Biobanking, Balgrist Campus AG, Zürich, Switzerland

[8] Instituto de Investigação e Inovação em Saúde, Translational Neuro-urology Group, Universidade do Porto, Porto, Portugal

For peer review only - http://bmjopen.bmj.com/site/about/guidelines.xhtml

[9] Faculdade de Medicina, Departemento de Biomedicina, Unidade de Biologia Experimental, Universidade do Porto, Porto, Portugal

[10] Spinal Cord Injury Center, Balgrist University Hospital, University of Zürich, Zürich, Switzerland

[11] REHAB Basel, Clinic of neurorehabilitation and paraplegiology, Basel, Switzerland

[12] Spinal Cord Injury Department, Clinique romande de réadaptation, Sion, Switzerland

[13] Urology Research Laboratory, DBMR, University of Bern, Bern, Switzerland

[14] Department of Gastroenterology and Hepatology, University Hospital Zürich, Zürich, Switzerland

[15] Institute for Regenerative Medicine, University of Zürich, Zürich, Switzerland

[16] Department of Trauma, University Hospital Zürich, Zürich, Switzerland

[17] Neuro-Urology, Swiss Paraplegic Centre, Nottwil, Switzerland

[18] Department of Radiology, Balgrist University Hospital, Zürich, Switzerland.

[19] Department of Urology, Inselspital, Bern University Hospital, Bern, Switzerland

# Corresponding author: Prof. Thomas M. Kessler, MD
Department of Neuro-Urology
Balgrist University Hospital
University of Zürich
Forchstrasse 340
8008 Zürich
Switzerland
Email: tkessler@gmx.ch

[a] VB, MDL, and CEA contributed equally

For peer review only

Word count: 3711

Key Words (MeSH): Urinary Bladder, Neurogenic; Urinary Bladder, Overactive; Transcutaneous Electric Nerve Stimulation; Spinal Cord Injuries; Clinical Trial Protocol

For peer review only - http://bmjopen.bmj.com/site/about/guidelines.xhtml

ABSTRACT

Introduction: Neurogenic lower urinary tract dysfunction (NLUTD), including neurogenic detrusor overactivity (NDO) and detrusor-sphincter dyssynergia, is one of the most frequent and devastating sequelae of spinal cord injury (SCI), as it can lead to urinary incontinence and secondary damage such as renal failure. Transcutaneous tibial nerve stimulation (TTNS) is a promising, non-invasive neuromodulatory intervention that may prevent the emergence of the C-fibre evoked bladder reflexes that are thought to cause NDO. This paper presents the protocol for TASCI (TTNS in Acute SCI), which will evaluate the efficacy of TTNS in preventing NDO. Furthermore, TASCI will provide insight into the mechanisms underlying TTNS, and the course of NLUTD development after SCI.

Methods and analysis: TASCI is a nationwide, randomised, sham-controlled, double-blind clinical trial, conducted at all four SCI centres in Switzerland. The longitudinal design includes a baseline assessment period 5-39 days after acute SCI and follow-up assessments occurring three, six, and twelve months after SCI. A planned 114 participants will be randomised into verum or sham TTNS groups (1:1 ratio), stratified on study centre and lower extremity motor score. TTNS is performed for 30-minutes per day, five days per week, for 6-9 weeks starting within 40 days after SCI. The primary outcome is the occurrence of NDO jeopardizing the upper urinary tract at one year after SCI, assessed by urodynamic investigation. Secondary outcome measures assess bladder and bowel function and symptoms, sexual function, neurological structure and function, functional independence, quality of life, as well as changes in biomarkers in the urine, blood, stool, and bladder tissue. Safety of TTNS is the tertiary outcome.

Ethics and dissemination: TASCI is approved by the Swiss Ethics Committee for Northwest/Central Switzerland, the Swiss Ethics Committee Vaud and the Swiss

For peer review only - http://bmjopen.bmj.com/site/about/guidelines.xhtml

Ethics Committee Zürich (#2019-00074). Findings will be disseminated through peer-reviewed publications.

Trial registration number: NCT03965299, ClinicalTrials.gov.

Article Summary: Strengths and limitations of this study

- This study is a nationwide, randomised, sham-controlled, double-blind clinical trial conducted at all four spinal cord injury (SCI) centres in Switzerland.
- Transcutaneous tibial nerve stimulation (TTNS) is a well-tolerated, low-risk, non-invasive intervention.
- The inpatient setting facilitates study procedures and monitoring that maximize intervention adherence.
- Secondary outcomes provide valuable information regarding the impact of TTNS on additional factors that are relevant to patients as well as clinicians, and also facilitate targeted analyses aimed at better understanding the mechanism underlying TTNS therapy, and the development of neurogenic lower urinary tract dysfunction after SCI.
- In comparison to beginning immediately after SCI, the intervention start at 29-40 days after SCI (in order to complete all baseline assessments) may modestly reduce the potential for neuroplasticity and the maximum achievable intervention effect.

For peer review only

INTRODUCTION

Most patients with spinal cord injury (SCI) progressively develop neurogenic lower urinary tract dysfunction (NLUTD),[1] which is one of the most devastating sequelae of SCI. Recovery of lower urinary tract (LUT) function is often identified as a top patient concern after SCI, and sometimes is even prioritized above walking.[2-4] The consequences of NLUTD, including urinary incontinence, dependence on catheters, and recurrent urinary tract infections, negatively impact quality of life (QoL).[5-7] On a neurological level, intact pathways between the brain and the LUT are required for the complex multilevel neural network governing the LUT to function properly.[8] SCI is thought to trigger neuroplastic changes in C-fibre-mediated spinal reflex pathways in the spinal cord, which lead to the emergence of aberrant micturition reflexes.[9 10] Thus, within a few weeks after acute SCI, most patients start experiencing involuntary bladder contractions, neurogenic detrusor overactivity (NDO), which can cause urinary incontinence.[8] Furthermore, NDO, in combination with detrusor sphincter dyssynergia (DSD) and/or low bladder compliance, can produce high intravesical pressure, which is a relevant risk factor for renal failure.[11 12] NDO also predisposes patients to vesico-uretero-renal reflux, which can endanger the upper urinary tract (UUT).[8 13] Accordingly, the current urological management approach is centred around protecting the UUT and treating long-term complications of NLUTD.[13 14] Antimuscarinic medication and intradetrusor onabotulinumtoxinA injections are the first- and second-line treatments for NDO, but both treatments are often discontinued due to limited effectiveness and bothersome side effects.[15-17] If conventional conservative and minimally invasive therapies fail, the only remaining options are more invasive treatments which are associated with substantial morbidity, such as bladder augmentation and urinary diversion.[18 19] While neuro-urological

For peer review only - http://bmjopen.bmj.com/site/about/guidelines.xhtml

treatment of patients with SCI has greatly improved during the last decades, it is only applied when NLUTD is already established with little chance of recovery.

Here we present the protocol for a randomised controlled trial (RCT) that aims at producing evidence for the use of non-invasive electrical stimulation (neuromodulation) to prevent the development of NDO. Early neuromodulatory therapy has the potential to impede or alter neuroplasticity, preventing the sensitisation of 'silent' C fibres and the formation of spinal reflex pathways thought to be responsible for NDO. Indeed, in a pilot study, bilateral sacral neuromodulation during the early phase of SCI prevented the development of NDO and also improved erectile and bowel function.[20] Furthermore, in animal models, early neuromodulatory interventions after SCI can modulate the micturition reflex[21] and inhibit detrusor overactivity.[22] In humans, non-invasive neuromodulatory techniques such as tibial nerve stimulation[23] and transcutaneous electrical nerve stimulation (TENS)[24] have shown promise in a treatment context[25-28] and warrant well-designed RCTs. Transcutaneous tibial nerve stimulation (TTNS) has generated interest, as a neuromodulatory treatment that is completely non-invasive (current is applied through surface electrodes) with no known safety risks, easy to apply, and well-accepted by patients. A randomised pilot trial in patients with acute SCI concluded that TTNS was feasible and safe in an inpatient rehabilitation setting.[29] Furthermore, at the end of the study, the participants in the intervention group had lower detrusor pressures and fewer developed DSD compared to the sham group,[30] suggesting that TTNS could alter the course of NLUTD. We hypothesize that TTNS applied in the acute phase after SCI will prevent the emergence of C-fibre evoked bladder reflexes, thereby the development of NDO, and avert UUT damage and urinary incontinence. TASCI (TTNS in Acute SCI) aims to provide evidence regarding the clinical efficacy of TTNS as a preventative measure for NDO, along with additional insight into the mechanism

For peer review only - http://bmjopen.bmj.com/site/about/guidelines.xhtml

underlying TTNS and the course of NLUTD development during the first year after SCI. We operationalized this RCT as a population-based study in the context of specialized rehabilitation for SCI in Switzerland.

METHODS AND ANALYSES

This protocol follows the recommendations of the SPIRIT (Standard Protocol Items: Recommendations for Interventional Trials) Statement (Supplement 1).[31]

Study design and setting

TASCI is a nationwide, randomised, sham-controlled, double-blind clinical trial investigating the effects of TTNS in patients with acute SCI.[32] The intervention will take place during the inpatient rehabilitation stay in one of the four SCI centres in Switzerland (Basel: REHAB Basel, Nottwil: Swiss Paraplegic Center, Sion: Clinique romande de réadaptation, and Zürich: Balgrist University Hospital). Furthermore, TASCI is nested in the inception cohort of the prospective Swiss Spinal Cord Injury (SwiSCI) cohort study.[33]

The TASCI schedule (Figure 1) includes a screening and baseline period from days 5-39 after SCI diagnosis, followed by a 6-9-week intervention period that ends when all 3-month follow-up assessments have been completed. Three follow-up visits are planned: the 3-month visit (day 91 +/- 10), 6-month visit (day 182 +/- 10), and 12-month visit (day 365 +/- 10). The one-year post-SCI measurement time point was chosen for the primary outcome variable because it represents the time when the patient's situation is stable enough to start considering invasive treatment options.[14] The additional time points were selected to maximize the chances of detecting treatment effects, as well as to coincide with routine follow-up assessments of patients with SCI.

For peer review only - http://bmjopen.bmj.com/site/about/guidelines.xhtml

Participants

All SCI patients admitted to rehabilitation in one of the participating centres are screened for eligibility using information from their routine clinical examinations. For a full listing of inclusion and exclusion criteria refer to Box 1. Eligible patients are then approached by an investigator or supervising clinician who obtains informed consent. For an English translation of the study information leaflet and declaration of consent see Supplement 2. Patients who consent undergo additional baseline assessments and are randomised after their eligibility for the trial has been confirmed.

For peer review only

For peer review only - http://bmjopen.bmj.com/site/about/guidelines.xhtml

Box 1. TASCI eligibility criteria

Inclusion Criteria

- Age ≥ 18 years
- Patients with acute SCI (traumatic SCI and sudden onset (<7 days) non-traumatic SCI), within 40 days after injury
- Patients with acute SCI at cervical or thoracic level
- Willingness to take part and follow the requirements of the protocol during TASCI (up to 1 year after SCI):
    - No percutaneous tibial nerve stimulation (PTNS)
    - No functional electrical stimulation (FES) apart from upper limb FES
    - No electrical muscle stimulation (EMS)
- Informed consent

Exclusion Criteria

- Contraindications to the investigational product
- Treatment with antimuscarinics or mirabegron
- DO with contractions greater than 40 cmH_2O at a bladder filling volume of less than 500 mL at baseline visit
- Known or suspected non-adherence, drug or alcohol abuse
- Inability to follow the procedures of the study, e.g. due to language problems, psychological disorders, dementia, etc. of the participant
- Participation in another study with investigational drug or product within the 30 days preceding and during the present study
- Neuromodulation treatment for urological or bowel indication in the last six months or ongoing
- Botulinum toxin injections in the detrusor and/or urethral sphincter in the last six months
- Bilaterally absent tibial nerve compound muscle action potential (cMAP, amplitude < 1mV)
- Women who are pregnant or breast feeding
- Intention to become pregnant during the course of the study
- Individuals especially in need of protection (according to Research with Human Subjects published by the Swiss Academy of Medical Sciences [www.samw.ch/en/News/News.html])
- Enrolment of the investigator, their family members, employees and other dependent persons
- Pre-existing or concomitant medical condition apart from SCI that might pose a safety issue or would interfere with interpretation of study results or study conduct (e.g. Parkinson's disease, neurodegenerative disorders including multiple sclerosis and amyotrophic lateral sclerosis, urological malignancies)

Urinary tract infection (UTI) is a transitory exclusion criterion for all functional assessments, including urodynamics. Before undergoing functional assessments, participants are screened for UTIs and haematuria. If a UTI is identified, treatment is initiated, and the functional assessments are rescheduled once the participant has been symptom-free for at least three days. DO, detrusor overactivity; SCI, spinal cord injury; TASCI, transcutaneous tibial nerve stimulation in acute spinal cord injury

For peer review only

For peer review only - http://bmjopen.bmj.com/site/about/guidelines.xhtml

Randomisation and blinding

Participants are randomly allocated with a 1:1 ratio into TTNS verum or TTNS sham stimulation groups, using computer-generated permuted block randomisation lists with varying block sizes. The randomisation lists are stratified according to study centre and the primary prognostic indicator, lower extremity motor score (LEMS) from the International Standards for Neurological Classification of SCI (ISNCSCI) assessment.[34] Prognostic models have shown that LEMS measured within 40 days of SCI is a reliable predictor of urinary continence and complete bladder emptying one year after SCI.[35] Based on this model, LEMS cut-offs of <12, 12-37, and >37 were chosen, which correspond to a population-average estimated probability of <20%, 20%-80% and >80% for urinary continence and complete bladder emptying at 1 year after SCI.[35] The allocation sequence and assignment of participants is centrally coordinated, and performed via the database software (Research Electronic Data Capture, REDCap[36 37]). To safeguard the allocation concealment, the randomisation table will be password secured and only accessible to investigators responsible for randomisation and application of the TTNS intervention.

To maintain blinding of the participants, care providers, and outcome assessors, throughout the duration of the participants' involvement in the study, the randomisation and TTNS intervention will be performed by trained operators, who are not involved in the clinical management. Additionally, the verum and sham TTNS setups are identical in terms of appearance, application procedure, and intervention duration, and all participants receive the same information.

Intervention

A commercially available TENS device (ELPHA II 3000, CE 0543 Certification, FH Service, Odense, Denmark) is used for both the TTNS verum and sham

For peer review only - http://bmjopen.bmj.com/site/about/guidelines.xhtml

intervention. The current frequency (20 Hz) and pulse width (200 μs) are pre-programmed (Figure 2). The current amplitude, which can be set between 0 and 100 mA, is manually adjusted for each participant during the initial set-up period. Each TTNS session starts with a preparatory phase where the participant is asked to lie in a comfortable supine position and a pillow or blanket is placed on the lower part of the leg to restrict the participant's view of their foot. Four electrodes are placed according to a pre-specified order on the foot and lower leg. The first electrode is placed 4-5cm above and 4-5cm posterior to the medial malleolus. The next two electrodes are placed around the 5th metatarsophalangeal joint, one dorsally over the bone, and one on the plantar fat pad. A final electrode is placed distal to, and in line with, the medial malleolus in the longitudinal arch of the foot (Figure 2). During an initial test period of several minutes, sensory and motor thresholds (if applicable) are assessed, current amplitudes are adjusted and the operator is carefully observing foot movement, i.e. flexion or fanning of the big toe or any other motor response. The operator may reposition electrodes to optimise the electrical stimulation setup. After the correct positioning of the electrodes has been verified by observation of the appropriate motor response,[23] the stimulation amplitude is reduced to a sub-motor level, defined as 0.5-1.0 mA below the motor threshold. In the sham setup, a cable with an interposed 1000 Ω resistance allows a current amplitude to be displayed and changed on the device without providing electrical stimulation to the participant.

The TTNS intervention is performed for 30 minutes per day, five days per week, starting within 40 days after SCI and ending after all 3-month follow-up assessments have been completed (total intervention period: 6-9 weeks). The majority of the participants are expected to be inpatients for the duration of the intervention, a setting that maximizes participant adherence. The application of the TTNS intervention by designated and trained personnel ensures that the treatment is

For peer review only - http://bmjopen.bmj.com/site/about/guidelines.xhtml

applied correctly while blinding is maintained. In addition, the remaining follow-up visits are part of standardized SCI care in all four participating centres, limiting the potential loss to follow-up, and ensuring that the participants receive necessary ancillary care.

Participants may be withdrawn when the study protocol is not followed, a health problem occurs with a suspected link to the stimulation, if a severe health condition/illness makes an intensive outpatient treatment or new hospitalization necessary, consent is withdrawn, or if a pregnancy is detected. Any concomitant treatment and medication intake will be carefully documented, so that the information can be utilized in secondary analyses.

Outcomes

The primary outcome is the occurrence of NDO jeopardizing the upper urinary tract up to 12 months after SCI, i.e. at 1 year after SCI or any earlier time point if NDO treatment is needed. This outcome is a composite measure including: NDO with an amplitude ≥ 40 cmH_2O identified during urodynamic investigation; or else the initiation of NDO treatment with antimuscarinics and/or intradetrusor onabotulinumtoxinA injections. Urodynamics will be performed before starting TTNS (within 40 days after SCI) at follow-up visits three-months, six-months and one year after SCI, or at any earlier time point if there is reason to believe that the participant has developed NDO, before starting NDO treatment.

Secondary outcomes were selected to further evaluate the effects of the TTNS intervention, and include clinical outcomes such as differential changes in LUT and bowel function and symptoms, sexual function, neurological function, as well as functional independence and QoL. Moreover, the evaluation of differential changes in brain and spinal cord function as well as cortico-spinal integrity, and biomarkers in

For peer review only - http://bmjopen.bmj.com/site/about/guidelines.xhtml

the urine, blood, stool, and bladder tissue will also provide insight into the mechanism of action underlying the TTNS intervention. All secondary outcomes may also be used to better understand the pathogenesis of NLUTD after SCI. Information collected will provide a detailed description of the disease course, and when possible, inform prognostic modelling and address causal questions. Finally, adverse events will be recorded for the duration of the participant's enrolment in the study to evaluate the safety of TTNS. Table 1 shows an overview of the outcome measurement assessments and assessment periods, and outcome parameters are listed in detail in Supplement 3.

Generally, the outcome assessments are administered at screening and baseline as well as during each of the three follow-up visits (Figure 1). One notable exception is the bladder biopsy, which will take place at baseline and the 6-month follow-up visit, and only with additional consent. Moreover, during the intervention period, participants will complete some of the questionnaires once per week, and the bladder and bowel diaries once every two weeks. Withdrawal from the study can occur at any time if treatment for NDO is indicated. If withdrawal occurs, all study completion assessments should be performed whenever possible. Furthermore, the trial is nested in the SwiSCI cohort study, so observation of the participants will continue through the cohort study after the end of TASCI. SwiSCI can provide long-term, open-label outcome data regarding morbidity, activities and participation (e.g. labour market participation), QoL and life expectancy.

For peer review only - http://bmjopen.bmj.com/site/about/guidelines.xhtml

Table 1. Secondary outcomes and outcome measures. Assessment periods are referenced to the date of spinal cord injury (SCI) diagnosis and include: screening and baseline (S&B) (days 5-39), 3-month follow-up (day 91 +/- 10), 6-month follow-up (day 182 +/-10), 12-month follow-up (day 365 +/-10). Additionally, some surveys are administered weekly during the TTNS treatment period (TTNS: weekly), and the bowel and bladder diaries are administered once every two weeks during the treatment period (TTNS: bi-weekly).

Outcome & Outcome Measure(s)	Assessment Periods
**Occurrence of neurogenic DO jeopardizing the upper urinary tract over a 1-year time course (time-to-event analysis)**	
Urodynamic investigation	S&B, 3, 6, 12 m
Clinical assessment	S&B, 3, 6, 12 m
**Differential changes in lower urinary tract function**	
Urodynamic investigation	S&B, 3, 6, 12 m
**Differential changes in lower urinary tract symptoms**	
Bladder diary	S&B, 3, 6, 12 m; TTNS: bi-weekly
International Prostate Symptom Score (IPSS)	S&B, 3, 6, 12 m; TTNS: weekly
Urinary Symptom Profile (USP)	S&B, 3, 6, 12 m; TTNS: weekly
**Differential changes in neurological structure and function**	
Neuro-urological physical examination	S&B, 3, 6, 12 m
International Standards for Neurological Classification of Spinal Cord Injury (ISNCSCI) assessment	S&B, 3, 6, 12 m
Nerve conduction studies (NCS)	S&B, 3, 6, 12 m
Electromyography (EMG)	S&B, 3, 6, 12 m
Electroencephalography (EEG)	S&B, 3, 6, 12 m
Transcranial magnetic stimulation (TMS)	S&B, 3, 6, 12 m
Magnetic resonance imaging (MRI)	S&B, 3, 6, 12 m
**Differential changes in bowel and pelvic floor function**	
Rectal sensitivity examination	S&B, 3, 6, 12 m
Anorectal manometry	S&B, 3, 6, 12 m
Barostat assessment	S&B, 3, 6, 12 m
**Differential changes in bowel symptoms**	
Bowel diary	S&B, 3, 6, 12 m; TTNS: bi-weekly
Neurogenic Bowel Dysfunction (NBD) Score	S&B, 3, 6, 12 m; TTNS: weekly
**Differential changes in sexual function**	
Female Sexual Function Index (FSFI)	S&B, 3, 6, 12 m
International Index of Erectile Function (IIEF)	S&B, 3, 6, 12 m
Clinical assessment	S&B, 3, 6, 12 m
**Differential changes in spasticity**	
Modified Ashworth Scale (MAS)	S&B, 3, 6, 12 m
Spinal Cord Injury Spasticity Evaluation Tool (SCI-SET)	S&B, 3, 6, 12 m; TTNS: weekly
**Differential changes in functional independence**	
Spinal Cord Independence Measure (SCIM) III	S&B, 3, 6, 12 m
**Differential changes in quality of life**	
International Prostate Symptom Score (IPSS)	S&B, 3, 6, 12 m; TTNS: weekly
Qualiveen	S&B, 3, 6, 12 m; TTNS: weekly
**Differential changes in biochemical and molecular biomarkers in the urine, blood, and stool, as well as in the bladder tissue**	
Histomorphology	S&B, 6 m
Enzyme-linked immunosorbent assay (ELISA)	S&B, 3, 6, 12 m
Western immunoblotting	S&B, 3, 6, 12 m
Reverse transcription polymerase chain reaction (RT-PCR)	S&B, 3, 6, 12 m
Microbiome analysis	S&B, 3, 6, 12 m
**Safety of TTNS**	
Clinical record check	Any time
Medical history	Any time
Health diary, confirmed by interview	Any time

DO, detrusor overactivity; m, months; SCI, spinal cord injury; S&B, screening and baseline; TTNS, transcutaneous tibial nerve stimulation

For peer review only - http://bmjopen.bmj.com/site/about/guidelines.xhtml

Sample size

Presuming a proportion of spontaneous success among the sham group of 0.15,[23] 114 participants (57 verum, 57 sham) need to be included to detect a difference of 0.25 in the proportion of participants who develop NDO, and thereby to correctly reject the null hypothesis with a probability (power) of 0.8. The type I error probability associated with the test of this null hypothesis is 0.05. Since the primary outcome variable is binary, this sample size analysis included a continuity correction to avoid type I error inflation.

Using prospective data collected by the SwiSCI inception cohort for the years 2014-2016 and applying a modified version of the TASCI inclusion/exclusion criteria, a total of 193 eligible patients are predicted during the 3-year recruitment period of TASCI. Anticipating a recruitment rate of 66%, this implies that the requisite 114 participants can be included within 3 years. Expected participant numbers per centre during the study period are 20 for Basel, 42 for Nottwil, 13 for Sion, and 39 for Zürich. Moreover, this data implies that participants who withdraw or drop out can be replaced.

Data collection and management

All investigators are responsible for complete and correct data entry. Data will be recorded in electronic case report forms (eCRFs) using an internet-based, secure database – REDCap,[36 37] which fulfils the Good Clinical Practice (GCP) requirements and is hosted at Balgrist University Hospital. Participants will be assigned a unique 10-digit identification number, and personal data (e.g. name) are not entered into the main study database. Confidentiality will be ensured by restricting the access to the trial database to only a minimal number of people, and by pseudonymisation of the data before analyses. The database includes built-in quality control mechanisms,

For peer review only - http://bmjopen.bmj.com/site/about/guidelines.xhtml

such as range-restricted data entry, and identification of missing data. A monitoring system clearly indicates that an expected assessment is late or missing. Additionally, a manual check is performed of each dataset after it is closed and marked as complete, first by a research assistant at the study site, then by a study coordinator.

Statistical analyses

The primary analysis will be an intention-to-treat analysis. The general statistical methodology for primary and secondary outcomes is comprised of basic descriptive statistics and multivariable regression analyses. Continuous variables will be tested for normality, and displayed as either means and standard deviations or medians and ranges; categorical variables will be displayed as frequencies and proportions. Multivariable regression techniques will be employed to control for possible confounding due to unequal distribution of baseline parameters across the trial arms, as well as systematic variation in outcomes across RCT strata, i.e. centre and LEMS. Longitudinal analysis techniques (e.g. mixed-effects models, structural equation models for growth, and time-to-event models) will be used to account for repeated measures, facilitating time-updated covariate adjustment and appropriately modelling trends with respect to time.

The primary outcome “the occurrence of NDO jeopardizing the upper urinary tract one year after SCI” is a binary response variable, which will be described using proportions with 95% binomial confidence intervals (CIs) and analysed using logistic regression, thus expressing treatment effect sizes as odds ratios (with 95% CIs). Sensitivity analyses will include a time-to-event analysis to evaluate the impact of TTNS on the risk of early drop-out or pre-term endpoints (< 1year). Protocol non-adherence, early withdrawals, and loss to follow-up will be examined and used to inform per-protocol sensitivity analyses. Sub-group analyses stratified on LEMS or

For peer review only - http://bmjopen.bmj.com/site/about/guidelines.xhtml

For peer review only

study centre may be performed if an intervention-subgroup interaction is found, with the understanding that these analyses will likely be underpowered. In cases where outcome data is used in an observational context, usually to examine prognosis or NLUTD aetiology, causal claims will be supported by an underlying theoretical framework.

Multiple imputation will be used when appropriate to account for missing data in the predictor variables.[38] Pattern mixture models may be applied to address missing outcome data for different scenarios of the missing data process.[39] Sensitivity analysis will be used to evaluate the robustness of the chosen imputation strategy. Any sensitivity analyses added after the breaking of the blind (post-hoc sensitivity analyses) will be clearly identified as such in the trial report.

No interim analyses are planned. In the event that severe clinical or neurological deterioration is detected in more than one subject, or on the recommendation of the study monitoring board following a serious adverse event, the study will be suspended until a comprehensive safety review has been completed. If the trial is suspended or halted, an interim analysis will be performed.

Quality assurance and safety oversight

Monitoring will be carried out by the Unit for Clinical and Applied Research (UCAR) at Balgrist University Hospital. Monitoring visits will occur at each study site before the start of the study, after three-month follow-up assessments have been completed for the first participant, and then annually thereafter, with a final closeout visit at study end. The site qualification and initiation visit(s) will assure that the study site has the appropriate facilities and personnel, and that the personnel are able to conduct the study according to protocol. Routine visits will focus on source document verification, in particular documents related to eligibility, consent, key outcome

For peer review only - http://bmjopen.bmj.com/site/about/guidelines.xhtml

parameters, and ensure the documentation and reporting of adverse events occurred in accordance with all legal, ethical and protocol-specific requirements.

Patient and public involvement

Feedback on the intervention procedure was obtained from patients and healthy volunteers during the piloting phase and used to refine the study protocol. All participants will receive a study update newsletter annually.

ETHICS AND DISSEMINATION

Ethics

TASCI will be conducted in accordance with the protocol and the principles contained in the current version of the Declaration of Helsinki, the GCP guidelines issued by the ICH, the European Regulation on medical devices 2017/745 and the ISO Norm 14155 and ISO 14971, the Swiss Law and Swiss regulatory authority's requirements. The study has been approved by the Swiss Ethics Committee for Northwest/Central Switzerland, the Swiss Ethics Committee Vaud and the Swiss Ethics Committee Zürich (2019-00074) and has been registered at ClinicalTrials.gov (NCT03965299). The ethics committees and regulatory authorities will receive annual safety and interim reports and be informed about study end. All investigators will respect the participant's right to confidentiality, and adhere to the Swiss Federal Act on Data Protection. There are no conflicts of interest.

Dissemination

The study results will be published in open-access peer-reviewed journals, and authorship will be defined according to the recommendations of the International Committee of Medical Journal Editors (ICMJE). All persons wishing to publish using

For peer review only - http://bmjopen.bmj.com/site/about/guidelines.xhtml

TASCI data must submit a project plan to the TASCI steering committee for approval before the data are released for analysis. Datasets and statistical coding will be deposited in a public repository, as long as participant privacy is not compromised. Results will be presented at conferences of relevant societies such as the American Urological Association (AUA), Deutschsprachige Medizinische Gesellschaft für Paraplegie (DMGP), European Association of Urology (EAU), International Continence Society (ICS), International Neuro-Urology Society (INUS), International Spinal Cord Society (ISCoS). Furthermore, these societies will be used as platforms to advocate the implementation of the study findings in daily clinical practice and incorporation into clinical guidelines. Dissemination among persons with SCI will be achieved through the newsletters Paraplegie and Paracontact as well as online media.

DISCUSSION

TASCI is the first RCT to investigate the efficacy of TTNS in preventing the development of NDO after acute SCI. It represents a transition in the approach to NLUTD management from treatment of manifested problems to prevention of the disorder itself. The structured follow-up of participants could further inform clinical practice, for example, providing much-needed evidence about the timing of key examinations. Moreover, the inclusion of 'research oriented' secondary outcomes will provide data that may shed some light on the mechanism underlying neuromodulatory therapy, which up until now, is essentially a black box. Additionally, the secondary outcome data could deepen the understanding of the natural trajectory of NLUTD development in the first year after SCI. A final potential benefit of this trial is that it utilizes a multidisciplinary approach to maximise the chances of identifying and understanding the potential preventative effects of neuromodulatory therapy. The

For peer review only - http://bmjopen.bmj.com/site/about/guidelines.xhtml

requisite collaboration among experts in neuro-urology, neuroscience, SCI, rehabilitation medicine, basic and translational research, and biostatistics improves the overall quality of the research and promotes future collaborative endeavours.

The successful development of a therapy that prevents NDO after SCI would represent a huge achievement, especially since there is no known cure, only management of consequences. An effective preventative measure could eliminate lifelong issues associated with SCI, lowering barriers to work force participation and improving QoL, at the same time reducing lifetime healthcare costs. Thus, TASCI represents an important and timely RCT with the potential to revolutionise clinical practice, as well as to provide conceptual insight regarding the mechanism of neuromodulation, and the developmental course of NLUTD following SCI.

Trial status

This publication is based on version 2 of the TASCI protocol dated October 29th, 2019. The official start of recruitment was on June 19th, 2019. The estimated end date of the trial is June 30th, 2024 and recruitment of patients is ongoing. As of the time of submission, temporary interruptions to recruitment due to the COVID-19 pandemic are a possibility and in case of extended disruption, a schedule adjustment may be required.

Acknowledgements: We thank all members of the TASCI Study Group for their support. We thank all the study participants for their involvement in this trial.

Author Contributions: TMK is the chief investigator and responsible for overseeing the trial. AC, JP, MWGB, and TMK are study PIs, were responsible for the study conception and obtained funding. XJ, SM, JP, and TMK are site PIs. The TASCI

For peer review only - http://bmjopen.bmj.com/site/about/guidelines.xhtml

steering committee consists of the study PIs and the site PIs. MDL and SvdL are the trial coordinators. All authors, VB, MDL, CEA, LMB, SBa, MB, LAB, SMB, SBü, CDC, GD, PF, SF, OG, MHG, KH, XJ, MK, LL, EL, UM, SM, FM, KM, NP, DP, HS, AMS, MS, KS, SAS, JS, CS, AT, CT, SvdL, JW, VZ, AC, JP, MWGB, TMK, contributed to the development of the methodology and study protocol and will contribute to the acquisition, analysis, and/or interpretation of the study data. VB, MDL, CEA, UM, FM, SAS, MS, CS, SvdL, and TMK developed and tested the TTNS intervention protocol. MDL, SBü, GD, PF, UM, FM, MS, SAS, SvdL, AC, and TMK developed the neurophysiology and MRI procedures. Clinical expertise was provided by VB, SBa, MB, PF, SF, OG, MHG, XJ, MK, LL, EL, UM, SM, NP, DP, HS, MS, KS, AT, CT, JW, VZ, AC, JP, and TMK. Translation research expertise was provided by LAB, SMB, CDC, KH, KM, AMS, and JS. CEA, LMB, and MWGB provided statistical expertise. VB, MDL, CEA, MWGB, and TMK drafted the manuscript. All authors reviewed and revised the manuscript before submission and approved its content.

Funding: This work is financially supported by the Swiss National Science Foundation (SNSF), grant number: SNSF, 33IC30_179644. Start-up funding was provided by the Swiss Paraplegic Foundation. Neither funding body had (or will have) a role in the study design; collection, management, analysis, and interpretation of data; writing of the report; or the decision to submit the report for publication.

Competing interests: None declared.

Data Access Statement: Data generated by this research will be anonymised and uploaded to a public repository after publication wherever legally and ethically

For peer review only - http://bmjopen.bmj.com/site/about/guidelines.xhtml

possible. When this is not possible, data will be made available upon reasonable request.

Patient consent for publication: Not required.

Ethics approval: The study has been approved by the Swiss Ethics Committee for Northwest/Central Switzerland, the Swiss Ethics Committee Vaud and the Swiss Ethics Committee Zürich (2019-00074) and has been registered at ClinicalTrials.gov (NCT03965299).

REFERENCES

1. Weld KJ, Dmochowski RR. Association of level of injury and bladder behavior in patients with post-traumatic spinal cord injury. *Urology* 2000;55(4):490-4. [published Online First: 2000/03/29]
2. Simpson LA, Eng JJ, Hsieh JT, et al. The health and life priorities of individuals with spinal cord injury: a systematic review. *Journal of neurotrauma* 2012;29(8):1548-55. doi: 10.1089/neu.2011.2226 [published Online First: 2012/02/11]
3. Lo C, Tran Y, Anderson K, et al. Functional Priorities in Persons with Spinal Cord Injury: Using Discrete Choice Experiments To Determine Preferences. *Journal of neurotrauma* 2016;33(21):1958-68. doi: 10.1089/neu.2016.4423 [published Online First: 2016/11/02]
4. Brinkhof MW, Al-Khodairy A, Eriks-Hoogland I, et al. Health conditions in people with spinal cord injury: Contemporary evidence from a population-based community survey in Switzerland. *Journal of rehabilitation medicine* 2016;48(2):197-209. doi: 10.2340/16501977-2039 [published Online First: 2016/03/05]
5. Liu CW, Attar KH, Gall A, et al. The relationship between bladder management and health-related quality of life in patients with spinal cord injury in the UK. *Spinal cord* 2010;48(4):319-24. doi: 10.1038/sc.2009.132 [published Online First: 2009/10/21]
6. Crescenze IM, Myers JB, Lenherr SM, et al. Predictors of low urinary quality of life in spinal cord injury patients on clean intermittent catheterization. *Neurourology and urodynamics* 2019 doi: 10.1002/nau.23983 [published Online First: 2019/03/27]
7. Myers JB, Lenherr SM, Stoffel JT, et al. Patient Reported Bladder Related Symptoms and Quality of Life after Spinal Cord Injury with Different Bladder Management Strategies. *The Journal of urology* 2019:101097JU0000000000000270. doi: 10.1097/ju.0000000000000270 [published Online First: 2019/04/09]
8. Panicker JN, Fowler CJ, Kessler TM. Lower urinary tract dysfunction in the neurological patient: clinical assessment and management. *The Lancet Neurology* 2015;14(7):720-32. doi: 10.1016/s1474-4422(15)00070-8 [published Online First: 2015/06/13]
9. de Groat WC, Yoshimura N. Mechanisms underlying the recovery of lower urinary tract function following spinal cord injury. *Progress in brain research* 2006;152:59-84. doi: 10.1016/s0079-6123(05)52005-3 [published Online First: 2005/10/04]

For peer review only - http://bmjopen.bmj.com/site/about/guidelines.xhtml

10. Blok BF, Holstege G. The pontine micturition center in rat receives direct lumbosacral input. An ultrastructural study. *Neuroscience letters* 2000;282(1-2):29-32. doi: 10.1016/s0304-3940(00)00833-8 [published Online First: 2000/03/14]
11. Shingleton WB, Bodner DR. The development of urologic complications in relationship to bladder pressure in spinal cord injured patients. *The Journal of the American Paraplegia Society* 1993;16(1):14-7. doi: 10.1080/01952307.1993.11735878 [published Online First: 1993/01/01]
12. Lawrenson R, Wyndaele JJ, Vlachonikolis I, et al. Renal failure in patients with neurogenic lower urinary tract dysfunction. *Neuroepidemiology* 2001;20(2):138-43. doi: 10.1159/000054774 [published Online First: 2001/05/19]
13. Groen J, Pannek J, Castro Diaz D, et al. Summary of European Association of Urology (EAU) Guidelines on Neuro-Urology. *European urology* 2016;69(2):324-33. doi: 10.1016/j.eururo.2015.07.071 [published Online First: 2015/08/26]
14. Welk B, Schneider MP, Thavaseelan J, et al. Early urological care of patients with spinal cord injury. *World journal of urology* 2018;36(10):1537-44. doi: 10.1007/s00345-018-2367-7 [published Online First: 2018/06/28]
15. Wollner J, Kessler TM. Botulinum toxin injections into the detrusor. *BJU international* 2011;108(9):1528-37. doi: 10.1111/j.1464-410X.2011.10675.x [published Online First: 2011/10/26]
16. Mangera A, Apostolidis A, Andersson KE, et al. An updated systematic review and statistical comparison of standardised mean outcomes for the use of botulinum toxin in the management of lower urinary tract disorders. *European urology* 2014;65(5):981-90. doi: 10.1016/j.eururo.2013.10.033 [published Online First: 2013/11/19]
17. Leitner L, Guggenbuhl-Roy S, Knupfer SC, et al. More Than 15 Years of Experience with Intradetrusor OnabotulinumtoxinA Injections for Treating Refractory Neurogenic Detrusor Overactivity: Lessons to Be Learned. *European urology* 2016;70(3):522-8. doi: 10.1016/j.eururo.2016.03.052 [published Online First: 2016/04/24]
18. Hoen L, Ecclestone H, Blok BFM, et al. Long-term effectiveness and complication rates of bladder augmentation in patients with neurogenic bladder dysfunction: A systematic review. *Neurourology and urodynamics* 2017;36(7):1685-702. doi: 10.1002/nau.23205 [published Online First: 2017/02/09]
19. Phe V, Boissier R, Blok BFM, et al. Continent catheterizable tubes/stomas in adult neuro-urological patients: A systematic review. *Neurourology and urodynamics* 2017;36(7):1711-22. doi: 10.1002/nau.23213 [published Online First: 2017/02/01]
20. Sievert KD, Amend B, Gakis G, et al. Early sacral neuromodulation prevents urinary incontinence after complete spinal cord injury. *Annals of neurology* 2010;67(1):74-84. doi: 10.1002/ana.21814 [published Online First: 2010/02/27]
21. Shi P, Zhao X, Wang J, et al. Effects of acute sacral neuromodulation on bladder reflex in complete spinal cord injury rats. *Neuromodulation : journal of the International Neuromodulation Society* 2013;16(6):583-9; discussion 89. doi: 10.1111/j.1525-1403.2012.00528.x [published Online First: 2012/11/07]
22. Li P, Liao L, Chen G, et al. Early low-frequency stimulation of the pudendal nerve can inhibit detrusor overactivity and delay progress of bladder fibrosis in dogs with spinal cord injuries. *Spinal cord* 2013;51(9):668-72. doi: 10.1038/sc.2013.60 [published Online First: 2013/06/26]
23. Schneider MP, Gross T, Bachmann LM, et al. Tibial nerve stimulation for treating neurogenic lower urinary tract dysfunction: a systematic review. *European urology* 2015;68(5):859-67. doi: 10.1016/j.eururo.2015.07.001 [published Online First: 2015/07/22]
24. Gross T, Schneider MP, Bachmann LM, et al. Transcutaneous electrical nerve stimulation for treating neurogenic lower urinary tract dysfunction: a systematic review. *European urology* 2016;69(6):1102-11. doi: 10.1016/j.eururo.2016.01.010 [published Online First: 2016/02/03]
25. Finazzi-Agro E, Petta F, Sciobica F, et al. Percutaneous tibial nerve stimulation effects on detrusor overactivity incontinence are not due to a placebo effect: a randomized,

For peer review only - http://bmjopen.bmj.com/site/about/guidelines.xhtml

double-blind, placebo controlled trial. *The Journal of urology* 2010;184(5):2001-6. doi: 10.1016/j.juro.2010.06.113 [published Online First: 2010/09/21]
26. Gad PN, Kreydin E, Zhong H, et al. Non-invasive Neuromodulation of Spinal Cord Restores Lower Urinary Tract Function After Paralysis. *Frontiers in neuroscience* 2018;12:432. doi: 10.3389/fnins.2018.00432 [published Online First: 2018/07/17]
27. Peters KM, Carrico DJ, Perez-Marrero RA, et al. Randomized trial of percutaneous tibial nerve stimulation versus Sham efficacy in the treatment of overactive bladder syndrome: results from the SUmiT trial. *The Journal of urology* 2010;183(4):1438-43. doi: 10.1016/j.juro.2009.12.036 [published Online First: 2010/02/23]
28. Finazzi-Agro E, Rocchi C, Pachatz C, et al. Percutaneous tibial nerve stimulation produces effects on brain activity: study on the modifications of the long latency somatosensory evoked potentials. *Neurourology and urodynamics* 2009;28(4):320-4. doi: 10.1002/nau.20651 [published Online First: 2008/12/19]
29. Stampas A, Korupolu R, Zhu L, et al. Safety, Feasibility, and Efficacy of Transcutaneous Tibial Nerve Stimulation in Acute Spinal Cord Injury Neurogenic Bladder: A Randomized Control Pilot Trial. *Neuromodulation : journal of the International Neuromodulation Society* 2019;22(6):716-22. doi: 10.1111/ner.12855 [published Online First: 2018/10/05]
30. Stampas A, Gustafson K, Korupolu R, et al. Bladder Neuromodulation in Acute Spinal Cord Injury via Transcutaneous Tibial Nerve Stimulation: Cystometrogram and Autonomic Nervous System Evidence From a Randomized Control Pilot Trial. *Frontiers in neuroscience* 2019;13:119-19. doi: 10.3389/fnins.2019.00119
31. Chan AW, Tetzlaff JM, Altman DG, et al. SPIRIT 2013: new guidance for content of clinical trial protocols. *Lancet (London, England)* 2013;381(9861):91-2. doi: 10.1016/s0140-6736(12)62160-6 [published Online First: 2013/01/12]
32. Liechti MD, van der Lely S, Stalder SA, et al. Update from TASCI, a Nationwide, Randomized, Sham-controlled, Double-blind Clinical Trial on Transcutaneous Tibial Nerve Stimulation in Patients with Acute Spinal Cord Injury to Prevent Neurogenic Detrusor Overactivity. *European urology focus* 2019 doi: 10.1016/j.euf.2019.09.019 [published Online First: 2019/10/12]
33. Post MW, Brinkhof MW, von Elm E, et al. Design of the Swiss Spinal Cord Injury Cohort Study. *American journal of physical medicine & rehabilitation / Association of Academic Physiatrists* 2011;90(11 Suppl 2):S5-16. doi: 10.1097/PHM.0b013e318230fd41 [published Online First: 2011/11/10]
34. Association ASI. International standards for neurological classification of spinal cord injury (ISNCSCI). Richmond, VA, USA: American Spinal Injury Association, 2019.
35. Pavese C, Schneider MP, Schubert M, et al. Prediction of Bladder Outcomes after Traumatic Spinal Cord Injury: A Longitudinal Cohort Study. *PLoS medicine* 2016;13(6):e1002041. doi: 10.1371/journal.pmed.1002041 [published Online First: 2016/06/22]
36. Harris PA, Taylor R, Minor BL, et al. The REDCap consortium: Building an international community of software platform partners. *Journal of biomedical informatics* 2019;95:103208. doi: 10.1016/j.jbi.2019.103208 [published Online First: 2019/05/13]
37. Harris PA, Taylor R, Thielke R, et al. Research electronic data capture (REDCap)--a metadata-driven methodology and workflow process for providing translational research informatics support. *Journal of biomedical informatics* 2009;42(2):377-81. doi: 10.1016/j.jbi.2008.08.010 [published Online First: 2008/10/22]
38. Sterne JA, White IR, Carlin JB, et al. Multiple imputation for missing data in epidemiological and clinical research: potential and pitfalls. *BMJ (Clinical research ed)* 2009;338:b2393. doi: 10.1136/bmj.b2393 [published Online First: 2009/07/01]
39. Carpenter J, Kenward, M.G. Multiple Imputatation and its Application: Wiley, Statistics in Practice 2013.

For peer review only - http://bmjopen.bmj.com/site/about/guidelines.xhtml

FIGURE LEGENDS

Figure 1: Trial flowchart. Safety data will be recorded on a continuous basis the entire time the participant is enrolled in the study.

* Additional clinical assessments target urological and bowel function and symptoms, neurological structure and function, sexual function, spasticity, functional independence, quality of life, and biochemical and molecular biomarkers in the urine, blood, stool, and in bladder tissue (additional consent required for bladder biopsy).

** Histomorphology is based on bladder biopsy which requires additional consent.

DO, detrusor overactivity; SCI, spinal cord injury; TTNS, transcutaneous tibial nerve stimulation.

Figure 2. TTNS Intervention. Pictures of the TTNS device, cables and electrode placements. A) The TTNS device has two channels that can be adjusted independently. Electrodes are placed in a specified, standardised order (1-4). B) Lateral view of the foot, showing the electrode placement on the dorsal bone and plantar fat pad of the 5th metatarsophalangeal joint. C) Medial view of the foot, electrodes are placed 4-5 cm proximal and posterior to the medial malleolus, as well as in the longitudinal arch of the foot, distal to, and in line with, the medial malleolus. TTNS, transcutaneous tibial nerve stimulation.

For peer review only

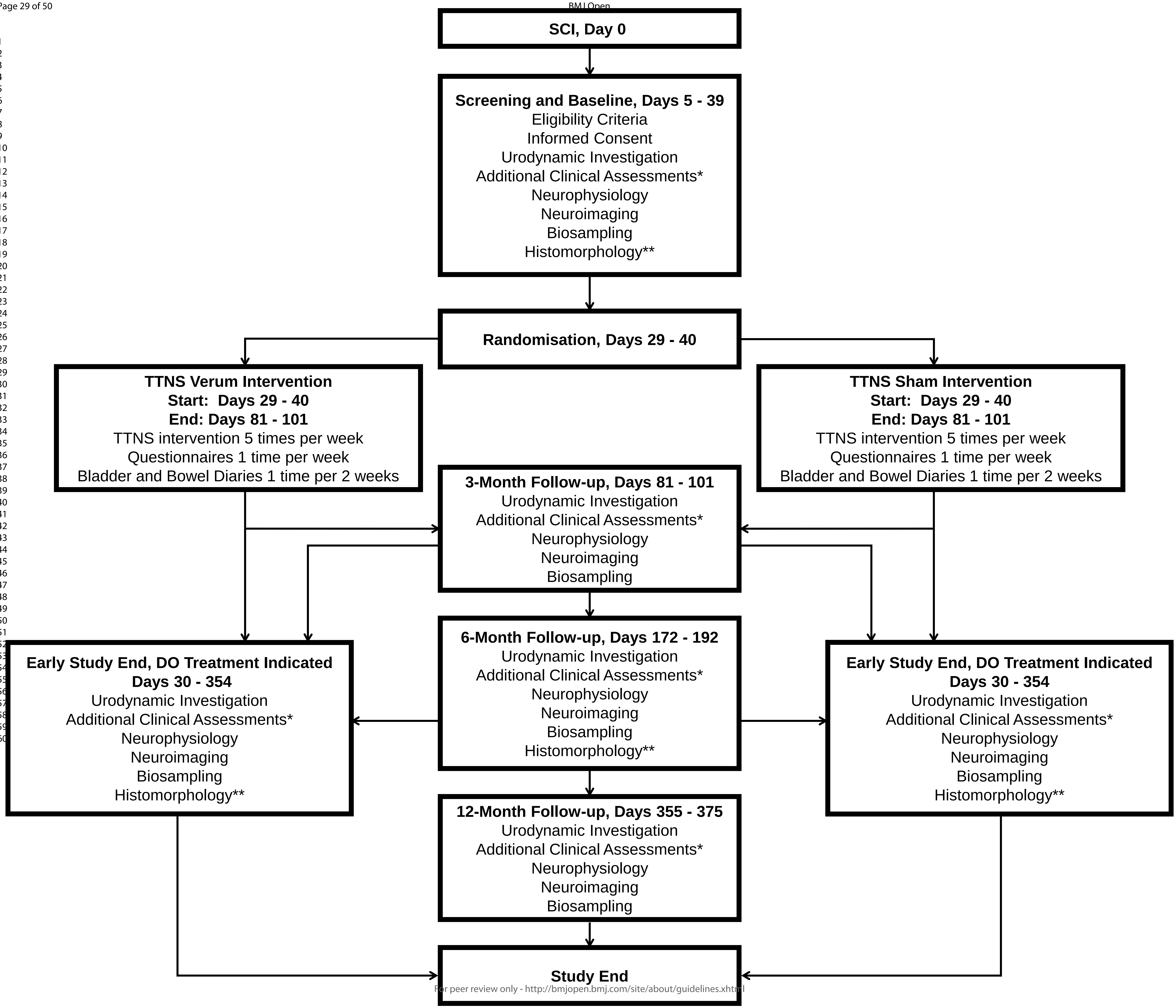
SCI, Day 0
Screening and Baseline, Days 5 - 39
Eligibility Criteria
Informed Consent
Urodynamic Investigation
Additional Clinical Assessments*
Neurophysiology
Neuroimaging
Biosampling
Histomorphology**
Randomisation, Days 29 - 40
TTNS Verum Intervention
Start: Days 29 - 40
End: Days 81 - 101
TTNS intervention 5 times per week
Questionnaires 1 time per week
Bladder and Bowel Diaries 1 time per 2 weeks
TTNS Sham Intervention
Start: Days 29 - 40
End: Days 81 - 101
TTNS intervention 5 times per week
Questionnaires 1 time per week
Bladder and Bowel Diaries 1 time per 2 weeks
3-Month Follow-up, Days 81 - 101
Urodynamic Investigation
Additional Clinical Assessments*
Neurophysiology
Neuroimaging
Biosampling
6-Month Follow-up, Days 172 - 192
Urodynamic Investigation
Additional Clinical Assessments*
Neurophysiology
Neuroimaging
Biosampling
Histomorphology**
Early Study End, DO Treatment Indicated
Days 30 - 354
Urodynamic Investigation
Additional Clinical Assessments*
Neurophysiology
Neuroimaging
Biosampling
Histomorphology**
Early Study End, DO Treatment Indicated
Days 30 - 354
Urodynamic Investigation
Additional Clinical Assessments*
Neurophysiology
Neuroimaging
Biosampling
Histomorphology**
12-Month Follow-up, Days 355 - 375
Urodynamic Investigation
Additional Clinical Assessments*
Neurophysiology
Neuroimaging
Biosampling
Study End

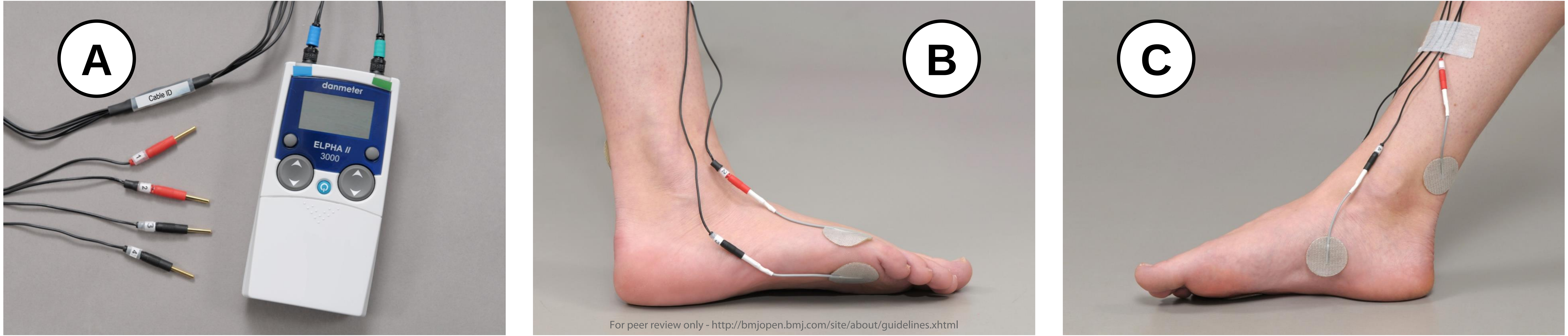

For peer review only - http://bmjopen.bmj.com/site/about/guidelines.xhtml

STANDARD PROTOCOL ITEMS: RECOMMENDATIONS FOR INTERVENTIONAL TRIALS

Supplement 1 – SPIRIT checklist for the TASCI (Transcutaneous Tibial Nerve Stimulation in Acute Spinal Cord Injury) Trial Protocol

SPIRIT 2013 Checklist: Recommended items to address in a clinical trial protocol and related documents*

Section/item	Item No	Description
**Administrative information**		
Title	1	Descriptive title identifying the study design, population, interventions, and, if applicable, trial acronym ✔ **Title, page 1**
Trial registration	2a	Trial identifier and registry name. If not yet registered, name of intended registry ✔ **Abstract, page 4**
	2b	All items from the World Health Organization Trial Registration Data Set – ✔ **Throughout the text, pages 1-21, and publicly accessible information on clinicaltrials.gov, NCT03965299**
Protocol version	3	Date and version identifier ✔ **Trial Status, page 20**
Funding	4	Sources and types of financial, material, and other support - ✔ **Funding Statement, page 21**
Roles and responsibilities	5a	Names, affiliations, and roles of protocol contributors ✔ **Title Page, pages 1-2; Contributor Statement, pages 20-21**
	5b	Name and contact information for the trial sponsor ✔ **Title Page (Corresponding Author), page 2**
	5c	Role of study sponsor and funders, if any, in study design; collection, management, analysis, and interpretation of data; writing of the report; and the decision to submit the report for publication, including whether they will have ultimate authority over any of these activities ✔ **Contributor Statement, Funding Statement , pages 20, 21**
	5d	Composition, roles, and responsibilities of the coordinating centre, steering committee, endpoint adjudication committee, data management team, and other individuals or groups overseeing the trial, if applicable (see Item 21a for data monitoring committee) ✔ **Dissemination, pages 18-19; Contributor Statement, pages 20-21**

For peer review only

Introduction

Background and rationale	6a	Description of research question and justification for undertaking the trial, including summary of relevant studies (published and unpublished) examining benefits and harms for each intervention ✔ **Introduction, pages 5-7**
	6b	Explanation for choice of comparators – ✔ **Introduction, page 6; Methods: Randomisation and blinding, Intervention, page 10**
Objectives	7	Specific objectives or hypotheses ✔ **Introduction, page 6-7**
Trial design	8	Description of trial design including type of trial (eg, parallel group, crossover, factorial, single group), allocation ratio, and framework (eg, superiority, equivalence, noninferiority, exploratory) ✔ **Methods: Study design and setting, page 7; Randomisation and blinding, page 10**

Methods: Participants, interventions, and outcomes

Study setting	9	Description of study settings (eg, community clinic, academic hospital) and list of countries where data will be collected. Reference to where list of study sites can be obtained ✔ **Methods: Study design and setting, page 7**
Eligibility criteria	10	Inclusion and exclusion criteria for participants. If applicable, eligibility criteria for study centres and individuals who will perform the interventions (eg, surgeons, psychotherapists) ✔ **Methods: Participants, page 8; Box 1: Eligibility criteria, page 9**
Interventions	11a	Interventions for each group with sufficient detail to allow replication, including how and when they will be administered ✔ **Methods: Intervention, pages 10-12. Please note: some details have been left vague in order to prevent participants from unblinding themselves with a Google search.**
	11b	Criteria for discontinuing or modifying allocated interventions for a given trial participant (eg, drug dose change in response to harms, participant request, or improving/worsening disease) ✔ **Methods: Intervention, page 12**
	11c	Strategies to improve adherence to intervention protocols, and any procedures for monitoring adherence (eg, drug tablet return, laboratory tests) ✔ **Methods: Intervention, pages 11-12**
	11d	Relevant concomitant care and interventions that are permitted or prohibited during the trial ✔ **Methods: Intervention, pages 11-12, Box 1 Eligibility criteria, page 9**

For peer review only - http://bmjopen.bmj.com/site/about/guidelines.xhtml

Outcomes	12	Primary, secondary, and other outcomes, including the specific measurement variable (eg, systolic blood pressure), analysis metric (eg, change from baseline, final value, time to event), method of aggregation (eg, median, proportion), and time point for each outcome. Explanation of the clinical relevance of chosen efficacy and harm outcomes is strongly recommended ✔ **Methods: Outcomes, pages 12-13; Table 1. Secondary outcomes and outcome measures, page 14; Supplement 3. Secondary outcome parameters**
Participant timeline	13	Time schedule of enrolment, interventions (including any run-ins and washouts), assessments, and visits for participants. A schematic diagram is highly recommended (see Figure) ✔ **Methods: Study design and setting, page 7, Intervention, pages 11-12, Figure 1. Trial Flowchart**
Sample size	14	Estimated number of participants needed to achieve study objectives and how it was determined, including clinical and statistical assumptions supporting any sample size calculations ✔ **Methods: Sample size, page 15**
Recruitment	15	Strategies for achieving adequate participant enrolment to reach target sample size **– Not explicitly mentioned, all patients with a spinal cord injury (SCI) coming through all Swiss specialized SCI centres will be screened for eligibility.**

Methods: Assignment of interventions (for controlled trials)

Allocation:

Sequence generation	16a	Method of generating the allocation sequence (eg, computer-generated random numbers), and list of any factors for stratification. To reduce predictability of a random sequence, details of any planned restriction (eg, blocking) should be provided in a separate document that is unavailable to those who enrol participants or assign interventions ✔ **Methods: Randomisation and blinding, page 10**
Allocation concealment mechanism	16b	Mechanism of implementing the allocation sequence (eg, central telephone; sequentially numbered, opaque, sealed envelopes), describing any steps to conceal the sequence until interventions are assigned ✔ **Methods: Randomisation and blinding, page 10**
Implementation	16c	Who will generate the allocation sequence, who will enrol participants, and who will assign participants to interventions ✔ **Methods: Participants, page 8, Randomisation and blinding, page 10**
Blinding (masking)	17a	Who will be blinded after assignment to interventions (eg, trial participants, care providers, outcome assessors, data analysts), and how ✔ **Methods: Randomisation and blinding, Intervention, pages 10 - 12**

	17b	If blinded, circumstances under which unblinding is permissible, and procedure for revealing a participant's allocated intervention during the trial **– Not explicitly mentioned, we do not foresee any need to unblind participants for the duration of their participation in the trial, as early evidence and clinical experience has indicated that the intervention is safe.**

Methods: Data collection, management, and analysis

Data collection methods	18a	Plans for assessment and collection of outcome, baseline, and other trial data, including any related processes to promote data quality (eg, duplicate measurements, training of assessors) and a description of study instruments (eg, questionnaires, laboratory tests) along with their reliability and validity, if known. Reference to where data collection forms can be found, if not in the protocol ✔ **Methods: Outcomes, pages 12-13; Table 1 Secondary outcomes, page 14; Supplement 3; Data collection and management, pages 15-16; Statistical Analyses, pages 16-17**
	18b	Plans to promote participant retention and complete follow-up, including list of any outcome data to be collected for participants who discontinue or deviate from intervention protocols ✔ **Methods: Intervention, pages 11-12, Outcomes, page 13**
Data management	19	Plans for data entry, coding, security, and storage, including any related processes to promote data quality (eg, double data entry; range checks for data values). Reference to where details of data management procedures can be found, if not in the protocol ✔ **Methods: Data management, pages 15-16**
Statistical methods	20a	Statistical methods for analysing primary and secondary outcomes. Reference to where other details of the statistical analysis plan can be found, if not in the protocol ✔ **Methods: Statistical analyses, pages 16-17**
	20b	Methods for any additional analyses (eg, subgroup and adjusted analyses) ✔ **Methods: Statistical analyses, pages 16-17**
	20c	Definition of analysis population relating to protocol non-adherence (eg, as randomised analysis), and any statistical methods to handle missing data (eg, multiple imputation) ✔ **Methods: Statistical analyses, pages 16-17**

Methods: Monitoring

Data monitoring	21a	Composition of data monitoring committee (DMC); summary of its role and reporting structure; statement of whether it is independent from the sponsor and competing interests; and reference to where further details about its charter can be found, if not in the protocol. Alternatively, an explanation of why a DMC is not needed ~ **There is a monitoring committee, but it is responsible for general trial conduct as well as data concerns. Sections: Data management, Quality assurance and oversight, pages 15-18**
	21b	Description of any interim analyses and stopping guidelines, including who will have access to these interim results and make the final decision to terminate the trial ✔ **Methods: Statistical analyses, page 17**
Harms	22	Plans for collecting, assessing, reporting, and managing solicited and spontaneously reported adverse events and other unintended effects of trial interventions or trial conduct ✔ **Methods: Outcomes, page 13; Table 1, page 14; Supplement 3**
Auditing	23	Frequency and procedures for auditing trial conduct, if any, and whether the process will be independent from investigators and the sponsor ✔ **Methods: Quality assurance and oversight, pages 17-18**

Ethics and dissemination

Research ethics approval	24	Plans for seeking research ethics committee/institutional review board (REC/IRB) approval ✔ **Ethics and Dissemination: Ethics, page 18**
Protocol amendments	25	Plans for communicating important protocol modifications (eg, changes to eligibility criteria, outcomes, analyses) to relevant parties (eg, investigators, REC/IRBs, trial participants, trial registries, journals, regulators) – **Not directly stated: by default in Switzerland all important protocol modifications have to be communicated to the ethics committees, who will pass information along regulators as appropriate. Trial participants receive an update letter, and there are representatives from each investigating centre on the steering committee.**
Consent or assent	26a	Who will obtain informed consent or assent from potential trial participants or authorised surrogates, and how (see Item 32) ✔ **Methods: Participants, page 8**
	26b	Additional consent provisions for collection and use of participant data and biological specimens in ancillary studies, if applicable ✔ **Methods: Outcomes, page 13**

For peer review only - http://bmjopen.bmj.com/site/about/guidelines.xhtml

Confidentiality	27	How personal information about potential and enrolled participants will be collected, shared, and maintained in order to protect confidentiality before, during, and after the trial ✔ **Methods: Data management, pages 15-16; Ethics and dissemination, pages 18-19**
Declaration of interests	28	Financial and other competing interests for principal investigators for the overall trial and each study site ✔ **Competing interests, page 21**
Access to data	29	Statement of who will have access to the final trial dataset, and disclosure of contractual agreements that limit such access for investigators ✔ **Contributor Statement, Dissemination, pages 18-19**
Ancillary and post-trial care	30	Provisions, if any, for ancillary and post-trial care, and for compensation to those who suffer harm from trial participation **-** ✔ **Methods: Intervention (ancillary care), pages 11-12. The need to compensate for harm is not foreseen, previous studies and clinical experience in other patient groups have not raised any safety concerns**
Dissemination policy	31a	Plans for investigators and sponsor to communicate trial results to participants, healthcare professionals, the public, and other relevant groups (eg, via pub lication, reporting in results databases, or other data sharing arrangements), including any publication restrictions ✔ **Ethics and Dissemination: Patient and public involvement, Dissemination, pages 18-19**
	31b	Authorship eligibility guidelines and any intended use of professional writers ✔ **Ethics and Dissemination: Dissemination, pages 18-19**
	31c	Plans, if any, for granting public access to the full protocol, participant-level dataset, and statistical code ✔ **Ethics and Dissemination: Dissemination, pages 18-19**
**Appendices**		
Informed consent materials	32	Model consent form and other related documentation given to participants and authorised surrogates ✔ **Supplement 2**
Biological specimens	33	Plans for collection, laboratory evaluation, and storage of biological specimens for genetic or molecular analysis in the current trial and for future use in ancillary studies, if applicable ✔ **Methods: Outcomes, pages 12-13; Figure 1; Table 1, page 14; Supplement 2, Supplement 3**

*It is strongly recommended that this checklist be read in conjunction with the SPIRIT 2013 Explanation & Elaboration for important clarification on the items. Amendments to the protocol should be tracked and dated. The SPIRIT checklist is copyrighted by the SPIRIT Group under the Creative Commons "Attribution-NonCommercial-NoDerivs 3.0 Unported" license.

Study information and declaration of consent

Transcutaneous tibial nerve stimulation in patients with acute spinal cord injury to prevent neurogenic bladder dysfunction

Official study acronym: **TASCI**
(Transcutaneous **T**ibial Nerve Stimulation in **A**cute **S**pinal **C**ord **I**njury)

This study is organised by: Balgrist University Hospital

Dear Sir or Madam

We would like to ask you to participate in a nationwide clinical study. The study investigates the effect of early phase electrical stimulation of the tibial nerve (transcutaneous tibial nerve stimulation (TTNS)) on bladder dysfunction after acute spinal cord injury. Below you will find a detailed description of the study. Please read the study information carefully before you decide whether or not you will participate.

Summary

1	**Study aim**<br>We would like to ask you to participate in our clinical trial. The study investigates whether early phase electrical stimulation of the tibial nerve can prevent the development of harmful bladder dysfunction.
2	**Selection for participation**<br>You have recently suffered a spinal cord injury at the level of the cervical or thoracic vertebrae. As bladder condition often deteriorates over the course of the first few weeks to months, you may benefit from early phase TTNS treatment. That is why we are giving you this study information.
3	**General information about the study**<br>Spinal cord injuries usually lead to bladder dysfunction, which often worsens over time and is associated with a risk for kidney failure and reduced quality of life. In this Switzerland-wide study we investigate the effect of early phase TTNS on bladder dysfunction after acute spinal cord injury. A total of 114 patients at the four specialized spinal cord injury centers in Switzerland, in Basel, Nottwil, Sion and Zurich, will be included in the study. Patients are randomly assigned to two groups: 57 patients will be treated with TTNS (verum), 57 patients will receive a sham treatment (sham). Neither patients nor the doctors treating them know which group the patients are assigned to, it is a double-blind study. The treatment is administered five times a week for 30 minutes, during a period of 6-9 weeks. The stimulation device (transcutaneous electrical nerve stimulation (TENS)) used for the treatment (ELPHA II 3000, medical device, CE 0543 Certification, FH Service) is CE certified and approved in Switzerland. The stimulation parameters correspond to the standards currently used in routine clinical practice for the treatment of chronic bladder dysfunction. The duration of the study for each individual participant is 1 year, with preliminary and follow-up examinations taking place at 4 fixed time points. The total duration of the study is 4 years.

For peer review only - http://bmjopen.bmj.com/site/about/guidelines.xhtml

4	**Procedure**<br>For you, the study will last for a total of 1 year, and for much of that time you will receive inpatient care at our clinic. During your hospital stay, you will undergo some baseline examinations for the study which will take place within the framework of your routine clinical examinations. This includes examinations of bladder pressure and bowel function, neurophysiological assessments, as well as magnetic resonance imaging for all suitable study participants. Furthermore, you will fill in a set of questionnaires about your condition (that is, your bladder and bowel function) and keep a bladder diary and a bowel diary for three or seven days, respectively.<br>This is followed by the 6-9 week treatment phase with five treatment sessions per week. Treatment groups are randomly assigned and neither you nor your study doctor knows which group you have been assigned to. Three, 6 and 12 months after spinal cord injury, follow-up examinations are performed, and they include the same tests as the baseline examinations. At the end of the study, you will learn which group you were assigned to. The baseline and follow-up examinations include assessments of your physical, neurological and urological condition, as well as analyses of blood, urine, and stool, and possibly also bladder tissue samples (at the end of the baseline examinations and 6 months after spinal cord injury).
5	**Benefit**<br>TTNS may have a positive effect on your bladder and sexual function. Through your participation we are gaining important information about the effectiveness of TTNS in preventing bladder problems.
6	**Rights**<br>You can freely decide whether or not you want to participate in the study. Your decision does not affect your medical care and you do not have to justify your decision.
7	**Responsibilities**<br>If you participate, we ask you to adhere to certain requirements. No other electrical stimulation of nerves or muscles (with the exception of the upper extremities) for therapeutic purposes may be performed on you for the entire duration of the study (12 months). You should inform your study doctor or study team of all treatments and medications you are currently receiving. Please talk to us as early as possible before any treatment changes are made, whether it is in the form of a change in dosage or the addition of a new treatment.
8	**Risks**<br>Overall, TTNS has an excellent safety profile, that is, it has almost no side effects. In very rare cases, skin reactions can occur in the area of where the surface electrodes are placed.<br>When measuring bladder pressure (urodynamic investigation) and stimulating the lower urinary tract, the insertion of the measurement catheters into the bladder and anus can be uncomfortable and can be associated with a slight burning sensation. Very rarely, the insertion of the catheter can lead to inflammation of the bladder, or injury to the urethra that could cause the urethra to narrow (urethral strictures).<br>The bowel assessments (anorectal manometry) are clinically established and are regarded as safe examinations. The insertion of the catheter can be perceived as unpleasant. A painful sensation indicates a medical problem, which can then be directly examined and, if necessary, treated.<br><u>When tissue samples are taken from the bladder (only applicable if you specifically agree to participate in this part of the study)</u>, a bladder infection or bleeding from the bladder may occur, which, very rarely, requires an additional procedure (cystoscopy) to stop the

For peer review only - http://bmjopen.bmj.com/site/about/guidelines.xhtml

	bleeding (haemostasis). In very rare cases, the intervention might cause a perforation (small hole) in the bladder. If this occurs, a catheter has to remain in the bladder for an extended period of time to allow the bladder to heal.
9	**Other treatment options**<br>Your doctor will discuss your other available treatment options with you.
10	**Results**<br>You will be informed of study results that are important for your health. If you do not want to receive this information, please inform your study doctor.
11	**Confidentiality of data and samples**<br>We adhere to all legal regulations regarding data protection and all parties involved are subject to the obligation of professional confidentiality. Your personal and medical data and your biological material / samples (blood, urine, etc.) are protected, and used in an encrypted form. If applicable: the data and samples you provide will only be used for other research projects if you give your separate consent.
12	**Withdrawal**<br>You can withdraw from the study at any time if you no longer wish to participate. The data and samples collected up to the time of your withdrawal will still be evaluated.
13	**Compensation**<br>There is no compensation for participating in this study.
14	**Liability**<br>The University of Zürich has taken out an insurance policy with Zürich Versicherungs-Gesellschaft AG (police: 14.970.888) for the research department of the Spinal Cord Injury Centre at Balgrist University Hospital (Forchstrasse 340, 8008 Zürich) to cover liability in the event of a possible claim.
15	**Funding**<br>The Swiss National Science Foundation (SNSF) is the primary source of funding for this study.
16	**Contact information**<br>If any questions, uncertainties or emergencies arise during or after the study, you can always contact the following persons:

Study Doctor (Principal Investigator):	Deputy:
Prof.Thomas M. Kessler, MD<br>Neuro-Urology / Spinal Cord Injury Centre<br>Balgrist University Hospital<br>Forchstrasse 340<br>8008 Zürich<br>phone +41 (0)44 386 39 07 (weekdays during working hours)	Dr. Ulrich Mehnert, MD, PhD<br>Neuro-Urology / Spinal Cord Injury Centre<br>Balgrist University Hospital<br>Forchstrasse 340<br>8008 Zürich<br>phone +41 (0)44 386 39 07 (weekdays during working hours)
+41 (0)44 386 11 11 (in case of emergency, outside of regular working hours)	

For peer review only - http://bmjopen.bmj.com/site/about/guidelines.xhtml

Detailed information

1. Study aim

Spinal cord injuries usually lead to bladder dysfunction, which can endanger kidney function and reduce quality of life. This study investigates whether early phase electrical stimulation of the tibial nerve (transcutaneous tibial nerve stimulation, TTNS) can prevent the development of harmful bladder dysfunction. To better understand the mechanism of this treatment, comprehensive neuro-urological, imaging, and laboratory, including histological (tissue sample), assessments will be performed.

2. Selection for participation

All adults who are affected by an acute spinal cord injury (up to 40 days after injury) at the cervical or thoracic vertebral level can participate. However, persons with contraindications to the use of electrical stimulation are excluded from this study.

3. General information

Bladder dysfunction is one of the most important problems of patients with spinal cord injury, and if it is left untreated, it can significantly reduce quality of life and cause damage to the upper urinary tract that can even lead to kidney failure. Current treatments are often insufficient or associated with significant side effects. We urgently need new treatments that can prevent bladder dysfunction before permanent damage occurs. TTNS is already successfully being used to treat existing bladder dysfunction.

In a nationwide study in which all specialized spinal cord injury centres in Switzerland (Basel, Nottwil, Sion and Zürich) are participating, we are investigating the preventive effect of early phase TTNS on bladder dysfunction resulting from acute spinal cord injury. A total of 114 patients will be included in the study over a period of four years and randomly assigned to two groups: 57 patients will be treated with TTNS (verum), 57 patients will receive a sham treatment (sham). Neither the patients nor the doctors treating them know which group the patient is assigned to, it is a double-blind study. The treatment is administered for 30 minutes, 5 times a week, for 6-9 weeks using adhesive electrodes placed on the foot. The stimulation device (transcutaneous electrical nerve stimulation (TENS)) used for the treatment (ELPHA II 3000, CE 0543 Certification, FH Service) is CE certified and approved in Switzerland. The current settings correspond to the standards used in routine clinical practice for the treatment of neurogenic bladder dysfunction.

The electrical impulses are conducted up the leg via the tibial nerve to the spinal cord (which plays an important role in controlling the bladder) and, if used repeatedly, leads to an improvement in bladder condition in many patients. It is thought that electrical impulses transmitted from the tibial nerve via the spinal cord to the brain have a positive effect on the bladder centres in the central nervous system, although the exact mechanisms of action are not yet fully understood. The repeated application of this electrical stimulation seems to be particularly important for the success of the treatment. Experience shows that noticeable improvement only occurs after several stimulation sessions. This type of therapy has proven to be effective in patients with chronic bladder problems and offers an excellent and almost side-effect free alternative to treatments such as Botox injections into the bladder muscle or operations on the bladder.
The duration of the study is 1 year for each study participant, with baseline and follow-up examinations taking place at 4 fixed time points.
In order to better understand the developments that occur after spinal cord injury and the mechanism of action of TTNS, comprehensive neuro-urological, imaging, and laboratory assessments (including histological analysis of tissue samples) will be carried out during the course of this study. Your bladder dysfunction will be assessed using questionnaires and the data from routine clinical examinations (bladder function examination, neuro-urological examination, neurophysiological examination, and analyses of blood, urine and stool samples) in combination with data from study-specific assessments. In addition, we are performing cellular-level investigations (using blood, urine, stool and bladder tissue samples) to identify important

For peer review only - http://bmjopen.bmj.com/site/about/guidelines.xhtml

mechanisms which for example, may control cell division, cell proliferation and cell death. Furthermore, you will undergo routine bowel function examinations, in particular regarding sensation in the area of the anus and rectum, as well as rectal pressure conditions and muscle strength. It is possible that findings from these examinations can also be used for diagnostic or treatment purposes at a later point in time.

The study will be conducted in accordance with Swiss regulations, and with all internationally recognised guidelines. The responsible cantonal ethics committee has evaluated and approved the study. A description of this study can also be found on the website of the Swiss Federal Office of Public Health: www.kofam.ch.

4. Study procedure

If you are eligible for this study according to our inclusion and exclusion criteria, a member of the study team will a member of the study team will discuss the study with you in detail (informed consent discussion). You will have time to ask questions and you will receive this study information to read and consent forms to sign. Once you are enrolled in this study, in addition to the TTNS treatment, you will undergo baseline and follow-up examinations that occur at fixed time points over a period of 1 year (see chart below). The individual assessments that make up these examinations can be spread over several days. The examinations can vary in terms of time and according to your state of health, but should not take more than half a day in the longest case.

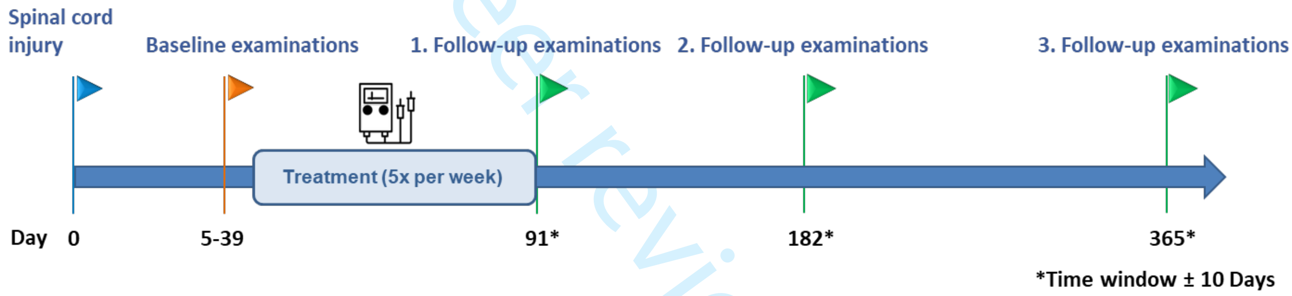

The **baseline examinations** take place up to 40 days after your spinal cord injury.

First, your medical history is taken, a list of your medications is collected, and you undergo a comprehensive medical examination:

- Physical examination
- Neurological examination:
  - Assessment of the severity of your paralysis: this includes an examination of your muscle strength, mobility, sensation (sensitivity to light touch and ability to distinguish between sharp and blunt objects) as well as sensation and conscious control of the anus
  - Reflexes
  - Muscle stiffness (spasticity)
  - Functional status (autonomy, breathing, management of bladder and bowel dysfunction, mobility)
  - Various walking tests for patients capable of walking, for example to record the walking aids used
- Assessment of health state, particularly bladder and bowel function using questionnaires:
  - Bladder emptying and sensation as well as catheter use
  - Your personal experiences, limitations and perceptions associated with your bladder dysfunction.
  - Bladder diary (over a period of 3 days): Documentation of times and amounts of fluid intake and urine excretion as well as incontinence episodes and use of adsorbent pads. We will provide you with a bladder diary and a urine measuring cup.
  - Bladder function (International Prostate Symptom Score, IPSS)
  - Urinary tract symptoms (Urinary Symptom Profile, USP)

For peer review only - http://bmjopen.bmj.com/site/about/guidelines.xhtml

- Quality of life (Qualiveen)
- Bowel function (Neurogenic Bowel Dysfunction Questionnaire, NBD)
- Sexual function (Female Sexual Function Index, FSFI; International Index of Erectile Function, IIEF)
- Spasticity after spinal cord injury (Spinal Cord Injury Spasticity Evaluation Tool, SCI-SET)

- Measurement of muscle function, nerve conduction, including nerve conduction velocity, as well as electrical activity in the brain. During these examinations, your nerves will be stimulated using short, light electrical impulses, among other things. For this purpose, adhesive electrodes (like those used for an electrocardiogram, (ECG)) or needle electrodes are placed at different positions on your body, for example, on your hand, or under the skin of your head, and a short electrical stimulus is triggered. This can be perceived as a tingling sensation. Sometimes this test is perceived as unpleasant. These sensations disappear when the test is finished and leave no side effects. This examination can last up to an hour.
- Bowel examinations
  - Anorectal manometry: in this clinical examination the pressure of the anal sphincter muscle, the sensation in the rectum and the neuronal reflexes are tested. This serves to assess the function of the anal sphincter. If abnormalities are found during the examination (for example, faecal incontinence, constipation or defecation disorders), the examination serves as a basis for further action.
  - Examination of the rectum and anus (proctoscopy): with this examination, diseases in the area of the anus, anal canal and lower rectum can be detected and, in some cases, treated directly.

Magnetic resonance imaging (MRI) is routinely used for capturing images of organs and tissues in a non-invasive and harmless manner. However, this examination cannot be performed if you have certain types of metal implants or a pacemaker, which you will be asked about separately. For the MRI examination, you must leave all metallic objects in a changing room. Before the examination, the scanning procedure will be explained to you one more time. You will then be accompanied to the MRI room where you will lie down on the examination table.
In order to make it as comfortable as possible for you to lie still, and to prevent unwanted movements, we use foam wedges, padding and velcro straps. You will be given headphones that you can use to communicate with the examiners at any time. You are positioned in the MRI machine and while you are lying very still on your back, images are taken of the spine and brain. In addition, brain function is measured at rest (resting activity, rMRI) and during electrical stimulation of the tibial nerve (functional MRI, fMRI). By repeating the assessment at several time points, we can detect changes that occur during the one-year course of the study. You will be able to listen to music during the some of the assessments. The MRI examination is radiation-free and it does not produce side effects. The headphones reduce the unpleasant noise caused by the machine. No contrast medium is required for the above-mentioned examinations, so no injections are administered.

As part of the normal hospital routine, **blood** and **urine samples** are collected from you, and slightly larger quantities will be taken for study purposes. In addition, stool samples will be collected, and, if you give your separate consent, tissue samples will also be collected from the bladder. The tissue samples are taken from the bladder under general or spinal (regional) anaesthesia using an optical instrument (cystoscope) with biopsy forceps / electrical loop inserted into the bladder through the urethra.

After completion of the baseline examinations, participants will be randomly divided into two treatment groups (actual TTNS (verum) or sham TTNS (sham)). You will be assigned to a treatment group using a random procedure, comparable to the tossing a coin (randomisation). The random assignment ensures that the groups are comparable. You cannot influence the treatment group assignment nor can your study doctor or any other person.

For peer review only - http://bmjopen.bmj.com/site/about/guidelines.xhtml

The treatment will be carried out by specially trained study personnel 5 times a week for 6-9 weeks until the first follow-up assessments. For the treatment, 4 adhesive electrodes are placed on the foot and during a preparatory phase, various current intensities and other parameters are tested to optimize the nerve stimulation conditions. Afterwards, the treatment, according to your group assignment, can begin. In order to ensure that the study data is credible and cannot be influenced, neither you nor your study doctor can know whether you are receiving verum or sham TTNS (this procedure is called "double-blind"). Every week you will fill in questionnaires to assess bladder awareness (University of South Australia Urinary Symptom Assessment (USA2)), bladder function (International Prostate Symptom Score (IPSS)) & Urinary Symptoms Profile (USP), spasticity (Spinal Cord Injury Spasticity Evaluation Tool (SCI-SET)), and quality of life (Qualiveen) as well as bowel function (Neurogenic Bowel Dysfunction Questionnaire (NBD)).

During the follow-up examinations, you will undergo repeats of the same clinical and research examinations that were performed at the study start. This means undergoing routine clinical examinations, filling in a bladder and bowel diary as well as all of the questionnaires mentioned above. Urine and blood samples are also collected again. In addition, bladder pressure, bowel function, and neurophysiological measurements are carried out, as well as an MRI examination. These examinations will occur 3, 6 and 12 months after spinal cord injury. During the second follow-up examination (6 months after spinal cord injury) if you consent to the removal of bladder tissue another sample will be taken. Your participation in the study will be complete after the 3rd follow-up examination (12 months after spinal cord injury).

We may have to prematurely end your participation in the study, if, for example, you have health problems that cannot be treated, or cannot be sufficiently treated, with electrotherapy or if the study protocol is not followed. In this case, you will undergo one final examination for your own safety. Your general practitioner will be informed about your participation in this study.

5. Benefits

If you participate in this study, early phase TTNS might prevent serious bladder dysfunction that is likely to occur due to your spinal cord injury. This could reduce the necessity of using long-term and often ineffective treatments, improve quality of life and reduce healthcare costs. This would be a milestone in the treatment of patients with spinal cord injury in the sense of "prevention is better than treatment". If you receive sham treatment, the course of the disease is unlikely to be affected. However, you will benefit from detailed examinations, which enhance monitoring of rehabilitation progress and allow for the immediate identification and treatment of health problems.

6. Rights

You participate in this study on a voluntary basis. If you do not wish to participate or later withdraw your consent, you do not need to justify your decision. Your medical care is guaranteed and independent of your decision to participate in the study. You may ask questions about the study at any time. The contact person, whose details are supplied at the end of this document, will be happy to answer your questions.

7. Responsibilities

As a participant, <u>over the entire study year</u>, it is necessary that you:

▪ adhere to the instructions and requirements outlined in the study plan (study protocol).

▪ inform your study doctor about your disease course and report any new symptoms, problems, or changes in your health condition (also after the end of the treatment phase or at the time of discontinuation of the study, for example, until any side effects disappear).

▪ always contact a member of the study team first to discuss any changes to your treatment plan (for example, changing the doses of your existing medications, adding a new medication or treatment, including complementary medicine and/or other treatments such as electrical muscle stimulation or similar treatments).

▪ bring a fully completed 3-day bladder diary and questionnaires with you to all baseline and follow-up examinations.

For peer review only - http://bmjopen.bmj.com/site/about/guidelines.xhtml

▪ do not receive any other electrical stimulation of nerves or muscles for therapeutic purposes (exception: functional electrical stimulation (FES) of the upper extremities), as it is sometimes used as a therapeutic approach during spinal cord injury rehabilitation. After the end of the study, these treatments can be used without restriction.

8. Risks and burdens for the participants

Overall TTNS has an excellent safety profile, meaning it has almost no side effects. In very rare cases, skin reactions in the area of the adhesive electrodes and/or an unpleasant tingling sensation in the feet with slight numbness may occur for a short time after the TTNS session. To date, severe, life-threatening or permanent damage due to TTNS have not been reported in any study.

For measuring bladder pressure (urodynamic investigation, a routine clinical procedure) and stimulating the lower urinary tract, bladder filling using an inserted catheter is necessary. Bladder filling can cause a feeling of tension and an unpleasant urge to urinate. In rare cases, the catheter insertion can cause a urinary tract infection, which would manifest as pain and a burning sensation when urinating. Very rarely, the urethra could be injured resulting in urethral narrowing (urethral stricture). If such symptoms occur, please inform the contact persons listed below immediately.

The only examination that is a source of radiation exposure is the video-urodynamic investigation, but this is a routine clinical examination which has a direct benefit for the patient. Patients with an acute spinal cord injury have a high risk of developing serious bladder problems, which in the worst case could lead to kidney failure. Therefore, all patients will undergo regular video-urodynamic investigations as part of their routine neuro-urological follow-ups.

Magnetic resonance imaging (MRI) has been an integral part of medical diagnostics for a long time. Images of the inside of the body are generated using radio frequency waves in a strong magnetic field. Magnetic resonance imaging is not known to cause harmful side effects. In particular, there is no radiation exposure. In order to avoid any risk, metallic or magnetic objects must be removed and stored before the examination.

Bowel examinations (anorectal manometry) are clinically established and are regarded as safe examinations. The insertion of the catheter can be perceived as unpleasant. Pain indicates a medical problem, which can then be directly examined and treated if necessary. In rare cases, the movement of the probe in the rectum and the inflation of the balloon can cause superficial injuries to the intestinal mucosa.

During **blood collection**, bruising may occur in the area of the puncture site. There is a very low risk of local or general infection. In extremely rare cases, injury to a nerve in the skin may occur, and possibly even progress to a chronic problem.

In rare cases, **taking tissue samples from the bladder** can cause a bladder infection or bleeding, which can very rarely result in a blood transfusion or a further intervention (cystoscopy) to control the bleeding (hemostasis). In extremely rare cases, a bladder perforation (small hole in the bladder) might occur during the intervention. In this case, a catheter has to remain in the bladder for an extended period of time to allow the bladder to heal. As with any procedure, general complications such as thromboses and embolisms can occur.

All other examinations and test procedures are non-invasive or minimally invasive.

For women who can become pregnant

For women of childbearing age (by definition not menopausal, last menstrual period within the last 12 months, no tubal ligation, ovaries and/or uterus not surgically removed) a free pregnancy test is carried out before the first examination. If this test is positive, you cannot participate in the study for your own safety. If you become pregnant during the study, you must inform your study doctor immediately, and may no longer participate in the study for your safety. In this case this does happen, you will be asked to provide information about the course and outcome of the pregnancy. The study doctor will discuss the procedures with you. If you are breastfeeding, you cannot participate in the study.

For peer review only - http://bmjopen.bmj.com/site/about/guidelines.xhtml

9. Other treatment options

There are currently no clinical treatment options to prevent the development of serious bladder dysfunction. If you do not wish to participate or wish to withdraw from the clinical trial at a later date, this will not affect your medical care.

10. Results of the study

The study doctor will inform you of any new knowledge acquired during the study that could affect your evaluation of potential benefits from the study or your safety and thus your consent to participate in the study. You will receive this information orally as well as in writing.
In principle, you will be informed about incidental findings (for example in MRI or genetic analyses) that may have implications for your present or future state of health, including the prevention, detection and treatment of existing or potential diseases. If you do not wish to be informed (so-called "right not to know"), please discuss this with your study doctor and ensure that you check the relevant box on your declaration of consent form.

11. Confidentiality of data and samples

For this study, your personal and medical data will be collected. Very few professionals will see your unencrypted data, and only to perform tasks within the study. When data is collected for study purposes, the data is encrypted. Encryption means that all reference data that could identify you (name, date of birth) is replaced by a code. The list of codes always remains in the study centres. Therefore, those persons who do not know the code cannot identify you. In scientific studies, the data is summarized and it is not possible to draw conclusions about you as an individual. Your name will never appear on the internet or in a publication. Sometimes scientific journals require that individual data (so-called "raw data") is submitted. If individual data have to be submitted, the data is always encrypted and therefore not traceable to you as a person. All persons who have access to your data within the scope of the study are subject to confidentiality obligations. All data protection regulations are observed, and you as a participating person have the right to view your data at any time.

If data / samples are stored on site, it is in a database / biobank for research purposes. These data and samples can be sent in encrypted form to another database / biobank within the framework of this study. The biological samples that were collected specifically for this study are usually no longer available for diagnostic purposes.

The data and biological samples will be shared in encrypted form for analysis purposes among the four participating study centres, and also sent to Bern (Switzerland), Leipzig (Max Planck Institute, Germany), London (United Kingdom), Porto (Portugal) and Pittsburgh (USA) for special analyses, where they will be examined for this project and then stored for 10 years. The code list will remain in the recruiting study centre and only the local study team will have access to it. The sponsor is responsible for ensuring that the international sites maintain the same standards as those in Switzerland.

It is possible that your data and samples may be used for other investigations at a later date or may be sent to another database / biobank in Switzerland or abroad for use in additional analyses that are not yet specified (further use). These other databases / biobanks must comply with the same standards as the database / biobank currently used in this study. To allow this further use, we ask you to sign the additional declaration of consent at the very end of this document.

It is possible that this study will be reviewed by the competent ethics committee, Swissmedic, or by the sponsor (or their representative responsible for quality assurance). The study team may need to disclose your personal and medical data for such reviews. It is possible that your treating physician may be contacted to provide information about your state of health. In the course of this project, data will be collected which may also be of interest to your attending doctor and thereby relevant for your personal medical care. Therefore, this data (for example: data from neurological examinations, bladder function, function of muscles and nerves, and magnetic resonance images)

For peer review only - http://bmjopen.bmj.com/site/about/guidelines.xhtml

may also be stored in your electronic medical file. This primarily serves to reduce duplicate examinations and thus to reduce your examination burden.

12. Withdrawal
You can withdraw from the study at any time if you no longer wish to participate. The data and samples collected up until that point will still be analysed (in encrypted form), so as not to lower the value of the project. It is not possible to anonymize your data and samples when you withdraw, meaning the data and samples remain encrypted. Please make sure that you agree to this before you participate in this study.

13. Compensation
You will not receive any compensation for participating in this study.

14. Liability
Balgrist University Hospital, which initiated the study and is responsible for conducting it, is liable for any damage you may suffer in relation to the medical product tested or to research activities (for example, the examinations). The requirements and procedure for this are regulated by law. The University of Zürich has an insurance policy with Zürich Versicherungs-Gesellschaft AG (police: 14.970.888) for the department of research at the Spinal Cord Injury Centre at Balgrist University Hospital (Forchstrasse 340, 8008 Zürich) to cover liability in the event of a claim.
In the event of damage that is attributable to an approved therapy administered in accordance with existing medical standards (certified medical device used according to the instructions) or that also would have occurred if a standard treatment had been used, the same liability rules apply as for treatments occurring outside of a research framework. If you have suffered damage, please contact your study doctor or the insurance company mentioned above. The necessary steps will then be taken for you.

15. Funding of the study
The Swiss National Science Foundation (SNSF) is the primary source of funding for this study.

16. Contact information
If any questions, uncertainties or emergencies arise during or after the study, you can always contact the following persons:

Study Doctor (Principal Investigator):	Deputy:
Prof. Thomas M. Kessler, MD<br>Neuro-Urology / Spinal Cord Injury Centre<br>Balgrist University Hospital<br>Forchstrasse 340<br>8008 Zürich<br>phone +41 (0)44 386 39 07 (weekdays during working hours)	Dr. Ulrich Mehnert, MD, PhD<br>Neuro-Urology / Spinal Cord Injury Centre<br>Balgrist University Hospital<br>Forchstrasse 340<br>8008 Zürich<br>phone +41 (0)44 386 39 07 (weekdays during working hours)
Phone: +41 (0)44 386 11 11 (in case of emergency, outside of regular working hours)	

For peer review only - http://bmjopen.bmj.com/site/about/guidelines.xhtml

Declaration of consent to participate in the TASCI study

Please read this form carefully. Please ask if you do not understand something or would like to know something. Your written consent is required for participation in this study.

**Title of the study**: Transcutaneous tibial nerve stimulation in patients with acute spinal cord injury to prevent neurogenic bladder dysfunction<br><br>(Full title: Transcutaneous tibial nerve stimulation in patients with acute spinal cord injury to prevent neurogenic detrusor overactivity: A nationwide randomised, sham-controlled, double-blind clinical trial)	
**Acronym/Short title:** TASCI	**BASEC-number: 2019-00074**
**Responsible institute (Sponsor with address**)	University of Zürich<br>c/o Balgrist University Hospital<br>Forchstrasse 340<br>8008 Zürich
**Study site**	Neuro-Urology / Spinal Cord Injury Centre<br>Balgrist University Hospital
**Responsible clinical investigator at the study site**<br>First name and last name (in block letters):	
**Participant**<br>First name and last name (in block letters):	
Gender: ☐ female ☐ male	Date of birth: __ __. __ __. __ __ __ __
Participant ID: ______________________	Screening-ID (SWISCI-ID): ________________

- I have been informed verbally and in writing by the undersigned clinical investigator about the purpose and procedure of this study using the stimulation device (ELPHA II 3000) and about possible advantages and disadvantages as well as possible risks.
- I am participating in this study on a voluntary basis and accept the contents of the written study information that has been provided to me. I have had sufficient time to make my decision.
- My questions regarding participation in this study have been answered. I will keep the written information and receive a copy of my consent form.
- I agree that my general practitioner will be informed about my participation in the study.
- I agree that the responsible expert representatives of the sponsor and the competent ethics committee may inspect my unencrypted data for examination and quality control purposes, but in strict compliance with confidentiality requirements.
- In the event that study results or incidental findings have direct implications for my present or future health, I will be informed unless I explicitly state otherwise. If I do not wish to be informed, I hereby notify my study doctor (clinical investigator) and confirm that I do not want to receive this type of information by checking the box below:

  ☐ I do NOT wish to be informed about results or incidental findings

For peer review only - http://bmjopen.bmj.com/site/about/guidelines.xhtml

- I am aware that my health-related and personal data (e.g. MRI and samples) can only be transferred in encrypted form (pseudonymised) for research purposes for this study (also abroad).
- In the event of further treatment outside the trial site, I authorize my general practitioner and attending doctors to transmit my post-treatment data relevant to the trial to the clinical investigator.
- I can withdraw from the study at any time and without justification. My continuing medical treatment is always guaranteed regardless of my participation in the study. Any data and samples collected up to the time of my withdrawal will be evaluated in the study.
- The liability insurance of the hospital/institution is responsible for covering any damages.
- I am aware that the requirements mentioned in the participant information must be fulfilled. In the interest of my health, the investigator may exclude me from the study at any time.
- I have also been informed about the accompanying research on molecular biological changes occurring in persons with bladder dysfunction.

☐ I agree,
☐ I do NOT agree,
that tissue samples can be collected from my bladder during the baseline examination and 6-month follow-up examination.

Place:<br><br>Date [dd.mm.yyyy]:<br><br>__ __. __ __. __ __ __ __	Signature of the participant

Confirmation by the investigator: I hereby confirm that I have explained the nature, significance and scope of the study to this participant. I confirm that I will fulfil all obligations in relation to this study in accordance with the applicable laws. If at any time during the course of the study I learn of anything that could influence the participant's willingness to participate in the study, I will inform him/her immediately.

Place:<br><br>Date [dd.mm.yyyy]:<br><br>__ __. __ __. __ __ __ __	Last name and first name of the investigator (in block letters):<br><br>Signature of the investigator

For peer review only - http://bmjopen.bmj.com/site/about/guidelines.xhtml

Consent to further use of data and biological material from this study for biomedical research

**Participant**<br>First name and last name (in block letters):	
Gender: ☐ female ☐ male	Date of birth: __ __. __ __. __ __ __ __
Participant ID:	Screening-ID. (SWISCI-ID):

I allow my data and samples from this study to be used for medical research purposes. This means that the health-related data (such as MRI images) and biological materials may be stored in a database or biobank and used for future, as of yet undefined, research projects for an indefinite period of time. This consent is valid for an unlimited period of time.

I am making this decision on a voluntary basis and can revoke this decision at any time. If I withdraw my consent, my data will be made anonymous and my samples will be destroyed. I only need to inform my study doctor (clinical investigator), and do not have to justify this decision.

I understand that the data and samples are encrypted and that the code list is stored securely. The data and samples can be sent to other databases and biobanks in Switzerland as well as abroad for analysis, provided that they maintain the same standards as in Switzerland. All legal requirements regarding data protection are fulfilled.

Usually, all data and samples are evaluated on a group level and the results are published in summary form. If a result that is important for my health should arise, it is possible that I will be contacted via my study doctor. If I do not wish to be contacted, I will inform my study doctor.

If results from the data and samples are commercialized, I have no claim to a share of the commercial use.

Place:<br><br>Date [dd.mm.yyyy]:<br><br>__ __. __ __. __ __ __ __	Signature of the participant:

Confirmation by the investigator: I hereby confirm that I have informed the participant of the nature, significance and scope of the further use of biological samples and/or genetic data.

Place:<br><br>Date [dd.mm.yyyy]:<br><br>__ __. __ __. __ __ __ __	Last name and first name of the investigator (in block letters):<br><br>Signature of the investigator:

For peer review only - http://bmjopen.bmj.com/site/about/guidelines.xhtml

Supplement 3. Secondary outcome parameters. Assessment periods are referenced to the date of spinal cord injury (SCI) diagnosis and include: screening and baseline (S&B) (days 5-39), 3-month follow-up (day 91 +/- 10), 6-month follow-up (day 182 +/-10), 12-month follow-up (day 365 +/-10). Additionally, some surveys are administered weekly during the TTNS treatment period (TTNS: weekly), and the bowel and bladder diaries are administered once every two weeks during the treatment period (TTNS: bi-weekly).

Outcome and Measurement Parameter (analysis metric)	Assessment Periods
**Occurrence of neurogenic detrusor overactivity (DO) jeopardizing the upper urinary tract over a 1-year time course assessed by: urodynamic investigation and clinical assessment**	
DO amplitude ≥40 cmH_2O, or initiation of DO treatment with antimuscarinics and/or intradetrusor onabotulinumtoxinA injections (time-to-event analysis)	S&B, 3, 6, 12 months
**Differential changes in lower urinary tract (LUT) function, assessed by urodynamic investigation**	
Maximum cystometric capacity (mL)	S&B, 3, 6, 12 months
Compliance (mL/cmH_2O)	S&B, 3, 6, 12 months
DO (present or absent), if present:	S&B, 3, 6, 12 months
Bladder volume at first DO (mL)	S&B, 3, 6, 12 months
Maximum DO amplitude (cmH_2O)	S&B, 3, 6, 12 months
Detrusor leak-point pressure (cmH_2O)	S&B, 3, 6, 12 months
Maximum detrusor pressure during storage phase (cmH_2O)	S&B, 3, 6, 12 months
Maximum detrusor pressure during voiding phase (cmH_2O)	S&B, 3, 6, 12 months
Detrusor pressure at maximum flow rate (cmH_2O)	S&B, 3, 6, 12 months
Maximum flow rate (mL/s)	S&B, 3, 6, 12 months
Voided volume (mL)	S&B, 3, 6, 12 months
Post void residual (mL)	S&B, 3, 6, 12 months
Pelvic floor electromyography (EMG) (normal/pathological)	S&B, 3, 6, 12 months
Videography (vesico-uretero-renal reflux, present/absent)	S&B, 3, 6, 12 months
Free flow curve pattern	S&B, 3, 6, 12 months
**Differential changes in lower spinal cord function, assessed by neuro-urological physical examination**	
Voluntary sphincter contraction (present/absent; if present, weak/strong)	S&B, 3, 6, 12 months
Resting anal tonus (weak/strong/spastic)	S&B, 3, 6, 12 months
Bulbocavernosus reflex(present/absent)	S&B, 3, 6, 12 months
Anal reflex (present/absent)	S&B, 3, 6, 12 months
Sensory testing S2-S5 (algesia, aesthesia: present/absent, even between sides, type of sensation)	S&B, 3, 6, 12 months
**Differential changes in LUT symptoms, assessed by bladder diary, International Prostate Symptom Score (IPSS), Urinary Symptom Profile (USP)**	
Number of voids per 24 hours	S&B, 3, 6, 12 months, TTNS: bi-weekly
Number of day-time voids	S&B, 3, 6, 12 months, TTNS: bi-weekly
Number of night-time voids	S&B, 3, 6, 12 months, TTNS: bi-weekly
Number of intermittent catheterizations	S&B, 3, 6, 12 months, TTNS: bi-weekly
Number of leakages per 24 hours	S&B, 3, 6, 12 months, TTNS: bi-weekly
Number of used pads per 24 hours	S&B, 3, 6, 12 months, TTNS: bi-weekly
Mean post void residual (mL)	S&B, 3, 6, 12 months, TTNS: bi-weekly
Mean voided volume (mL)	S&B, 3, 6, 12 months, TTNS: bi-weekly
Pain level (scale, 0-10)	S&B, 3, 6, 12 months, TTNS: bi-weekly
Level of urgency (scale, 0-4)	S&B, 3, 6, 12 months, TTNS: bi-weekly
IPSS Symptom Score (sum score, 0-35)	S&B, 3, 6, 12 months, TTNS: weekly
USP Stress urinary incontinence score (sum score, 0-9)	S&B, 3, 6, 12 months, TTNS: weekly
USP Overactive bladder score (sum score, 0-21)	S&B, 3, 6, 12 months, TTNS: weekly
USP Low stream score (sum score, 0-9)	S&B, 3, 6, 12 months, TTNS: weekly

For peer review only

**Differential changes in quality of life, assessed by IPSS, Qualiveen**	
IPSS Quality of life score (scale, 0-6)	S&B, 3, 6, 12 months, TTNS: weekly
Qualiveen total score (mean score, 0-4)	S&B, 3, 6, 12 months, TTNS: weekly
Qualiveen Limitations / Inconvenience subscale (mean score, 0-4)	S&B, 3, 6, 12 months, TTNS: weekly
Qualiveen Constraints / Restrictions subscale (mean score, 0-4)	S&B, 3, 6, 12 months, TTNS: weekly
Qualiveen Fears subscale (mean score, 0-4)	S&B, 3, 6, 12 months, TTNS: weekly
Qualiveen Feelings / Impact on Daily Life subscale (mean score, 0-4)	S&B, 3, 6, 12 months, TTNS: weekly
**Differential changes in sexual function, assessed by clinical assessment, FSFI (females), IIEF (males)**	
Psychogenic erection, M (present / reduced / absent / unknown / not applicable)	S&B, 3, 6, 12 months
Reflex erection, M (present / reduced / absent / unknown / not applicable)	S&B, 3, 6, 12 months
Ejaculation, M (present / reduced / absent / unknown / not applicable)	S&B, 3, 6, 12 months
Orgasmic function, M, F (present / reduced / absent / unknown / not applicable)	S&B, 3, 6, 12 months
Psychogenic genital arousal, F (present / reduced / absent / unknown / not applicable)	S&B, 3, 6, 12 months
Reflex genital arousal, F (present / reduced / absent / unknown / not applicable)	S&B, 3, 6, 12 months
Menstruation, F (present / reduced / absent / unknown / not applicable)	S&B, 3, 6, 12 months
FSFI, F total score (sum score, 0-36)	S&B, 3, 6, 12 months
FSFI, F Desire domain (sum score, 0-6)	S&B, 3, 6, 12 months
FSFI, F Arousal domain (sum score, 0-6)	S&B, 3, 6, 12 months
FSFI, F Lubrication domain (sum score, 0-6)	S&B, 3, 6, 12 months
FSFI, F Orgasm domain (sum score, 0-6)	S&B, 3, 6, 12 months
FSFI, F Satisfaction domain (sum score, 0-6)	S&B, 3, 6, 12 months
FSFI, F Pain domain (sum score, 0-6)	S&B, 3, 6, 12 months
IIEF, M total score (sum score, 5-75)	S&B, 3, 6, 12 months
IIEF, M Erectile Function domain (sum score, 1-30)	S&B, 3, 6, 12 months
IIEF, M Orgasmic Function domain (sum score, 0-10)	S&B, 3, 6, 12 months
IIEF, M Sexual Desire domain (sum score, 2-10)	S&B, 3, 6, 12 months
IIEF, M Intercourse Satisfaction domain (sum score, 0-15)	S&B, 3, 6, 12 months
IIEF, M Overall Satisfaction domain (sum score, 2-10)	S&B, 3, 6, 12 months
**Differential changes in bowel symptoms, assessed by NBD, bowel diary**	
NBD Score (version from ISCoS Bowel Function Dataset) (sum score, 0-47)	S&B, 3, 6, 12 months, TTNS: weekly
Number of defecations per 24h	S&B, 3, 6, 12 months, TTNS: bi-weekly
Number of urgency episodes per 24h	S&B, 3, 6, 12 months, TTNS: bi-weekly
Quantity of stool per defecation (Small/Medium/Large)	S&B, 3, 6, 12 months, TTNS: bi-weekly
Feeling of incomplete evacuation per 24h	S&B, 3, 6, 12 months, TTNS: bi-weekly
Number of accidental bowel leakages per 24h	S&B, 3, 6, 12 months, TTNS: bi-weekly
Quantity per bowel leakage (Small/Medium/Large)	S&B, 3, 6, 12 months, TTNS: bi-weekly
Stool consistency score (Bristol Stool Scale)	S&B, 3, 6, 12 months, TTNS: bi-weekly
Pain related to defecation (score)	S&B, 3, 6, 12 months, TTNS: bi-weekly
Medications for bowel	S&B, 3, 6, 12 months, TTNS: bi-weekly
**Differential changes in bowel function and pelvic floor function, assessed by rectal sensitivity examination, anorectal manometry, and barostat assessment**	
Initial sensation (mL)	S&B, 3, 6, 12 months
Urgency to defecate (mL)	S&B, 3, 6, 12 months
Maximum tolerated volume (mL)	S&B, 3, 6, 12 months
Basal internal anal sphincter pressure (mmHg)	S&B, 3, 6, 12 months
Squeeze external anal sphincter pressure (mmHg)	S&B, 3, 6, 12 months
Relaxation internal anal sphincter pressure during defecation (%, mmHg)	S&B, 3, 6, 12 months
Intraabdominal pressure during defecation (mmHg)	S&B, 3, 6, 12 months

For peer review only

Defecatory disorder (Rao Classification I-IV)	S&B, 3, 6, 12 months
Rectal capacity (mL)	S&B, 3, 6, 12 months
Rectal compliance (mL/mmHg)	S&B, 3, 6, 12 months
**Differential changes in motor and sensory function, assessed by the International Standards for Neurological Classification of Spinal Cord Injury (ISNCSCI) protocol**	
American Spinal Injury Association (ASIA) Impairment scale (AIS) Grade (A,B,C,D,E)	S&B, 3, 6, 12 months
ISNCSCI upper extremity motor score (UEMS) (score, 0-50)	S&B, 3, 6, 12 months
ISNCSCI lower extremity motor score (LEMS) (score, 0-50)	S&B, 3, 6, 12 months
ISNCSCI light touch (LT) score (score, 0-112)	S&B, 3, 6, 12 months
ISNCSCI pin prick (PP) score (score, 0-112)	S&B, 3, 6, 12 months
**Differential changes in functional independence assessed by the Spinal Independence Measure (SCIM) III**	
SCIM III Score (sum score, 0-100)	S&B, 3, 6, 12 months
**Differential changes in spasticity, assessed by Spinal Cord Injury Spasticity Evaluation Tool (SCI-SET), Modified Ashworth Scale (MAS)**	
SCI-SET Score (sum score, -105 to +105; mean score: -3 to +3, number of positive responses, number of negative responses)	S&B, 3, 6, 12 months, TTNS: weekly
Muscle tone of knee flexors and extensors (scale, 0-4)	S&B, 3, 6, 12 months
Muscle tone of elbow flexors and extensors (scale, 0-4)	S&B, 3, 6, 12 months
**Differential changes in brain and spinal cord function as well as cortico-spinal integrity, assessed using neurophysiological and neuroimaging methods: nerve conduction studies (NCS), EMG, electroencephalography (EEG), transcranial magnetic stimulation (TMS)**	
Latencies and amplitudes of F-waves to electrical stimulation	S&B, 3, 6, 12 months
Compound muscle action potentials (cMAP)	S&B, 3, 6, 12 months
Current perception thresholds (CPTs)	S&B, 3, 6, 12 months
Sensory evoked potentials (SEPs): latencies and amplitudes in response to electrical nerve stimulation	S&B, 3, 6, 12 months
Long latency sensory evoked potentials (LL-SEPs): latencies and amplitudes, in response to electrical nerve stimulation	S&B, 3, 6, 12 months
Sympathetic skin response (SSR): latencies and amplitudes	S&B, 3, 6, 12 months
Frequency and coherence measures of surface EMG and EEG	S&B, 3, 6, 12 months
Lower urinary tract sensory evoked potentials (LUTSEPs): latencies and amplitudes in response to electrical LUT stimulation	S&B, 3, 6, 12 months
Changes in white and grey matter area in the lumbosacral enlargement (LSE) and their relation to clinical outcomes	S&B, 3, 6, 12 months
Changes in white and grey matter area in upper cervical spinal cord (at C2/C3) and their relation to clinical outcomes	S&B, 3, 6, 12 months
Changes in white and grey matter volume of the conus medullaris (CM) and their relation to clinical outcomes	S&B, 3, 6, 12 months
Changes in grey and white matter volume in the brain and their relation to clinical outcomes	S&B, 3, 6, 12 months
Changes in fractional anisotropy (FA) in the brain and spinal cord and their relation to clinical outcomes	S&B, 3, 6, 12 months
Changes in diffusivity in the brain and spinal cord and their relation to clinical outcomes	S&B, 3, 6, 12 months
Changes in brain and spinal cord tissue microstructure and their relation to clinical outcomes	S&B, 3, 6, 12 months
Changes in brain function and connectivity during rest and electrical stimulation of the tibial nerve and their relation to clinical outcomes	S&B, 3, 6, 12 months
**Differential changes in biochemical and molecular biomarkers in urine, blood, stool, and bladder tissue, assessed by histomorphology, enzyme-linked immunosorbent assay (ELISA), western blot, reverse transcription polymerase chain reaction (RT-PCR), microbiome analysis**	
Biomarkers reflecting oxidative stress (presence/absence and quantities of peptides, proteins, antibodies, and small molecules)	S&B, 3, 6, 12 months
Expression profile of messenger RNA (mRNA) and microRNA (miRNA) in urine and blood as well as their relation to clinical outcomes	S&B, 3, 6, 12 months
Changes in inflammatory markers in bladder tissue, blood, and urine, as well as their relation to clinical outcomes	S&B, 3, 6, 12 months
Changes in levels of neurotransmitters (neurotrophins) in blood, and urine, as well as their relation to clinical outcomes	S&B, 3, 6, 12 months
Changes in the composition of urinary and stool microbiomes (analysed by sequencing and microbial culturing) and their relation to clinical outcomes	S&B, 3, 6, 12 months
Changes in the density / quality of afferent / efferent innervation of the bladder tissue and their relation to clinical outcomes	S&B, 6 months

For peer review only

**Safety of TTNS, assessed by clinical record check, patient interview, health diary (confirmed by clinician)**	
Urinary tract infection (UTI) (number of occurrences and intensity/severity)	Any time
Infection other than UTI (number of occurrences and intensity/severity)	Any time
Skin irritation at the electrode site (number of occurrences and intensity/severity)	Any time
Irritation-related symptoms (e.g. dysuria, hematuria) with a temporal link to a LUT intervention (i.e. immediately following the intervention) (number of occurrences and intensity/severity)	Any time
Neuro-urological deterioration (number of occurrences and intensity/severity)	Any time
Pressure ulcer (location, number of occurrences and intensity/severity)	Any time
Neurological deterioration (number of occurrences and intensity/severity)*	Any time
Increase in pain (number of occurrences and intensity/severity)*	Any time
Increase in spasticity (number of occurrences and intensity/severity)*	Any time
Deep vein thrombosis / pulmonary embolism (number of occurrences and intensity/severity)	Any time
Autonomic dysreflexia (number of occurrences (suspected and verified) and intensity/severity)	Any time
Transfer/admittance to an acute care facility (number of occurrences and intensity/severity)*	Any time

*only events that qualify as adverse events and serious adverse events are recorded
cMAP compound muscle action potentials; CPTs, current perception thresholds; DO, detrusor overactivity; ELISA, enzyme-linked immunosorbent assay; EEG, electroencephalography; EMG, electromyography; F, female; FSFI, Female Sexual Function Index; IIEF, International Index of Erectile Dysfunction; IPSS, International Prostate Symptom Score; ISNCSCI, the International Standards for Neurological Classification of Spinal Cord Injury; LL-SEP, long latency sensory evoked potential; LUT, lower urinary tract; LUTSEP lower urinary tract sensory evoked potential; M, male; MAS, Modified Ashworth Scale; miRNA, microRNA; mRNA, messenger RNA; NBD, neurogenic bowel dysfunction; NCS, nerve conduction studies; RT-PCR, Reverse transcription polymerase chain reaction; S&B, screening and baseline; SCI, spinal cord injury; SCI-SET, Spinal Cord Injury Spasticity Evaluation Tool; SCIM, Spinal Cord Independence Measure; SEP, sensory evoked potentials; SSR, Sympathetic skin response; TMS, transcranial magnetic stimulation; TTNS, transcutaneous tibial nerve stimulation; USP Urinary Symptom Profile; UTI, urinary tract infection

For peer review only
