# Supplementary material for: TASCI—transcutaneous tibial nerve stimulation in patients with acute spinal cord injury to prevent neurogenic detrusor overactivity: protocol for a nationwide, randomised, sham-controlled, double-blind clinical trial
Source: BMJ Open. 2020 Aug 13;10(8):e039164. doi: 10.1136/bmjopen-2020-039164 (PMC7430472; doi:10.1136/bmjopen-2020-039164)
Supplement: Reviewer comments [file bmjopen-2020-039164.reviewer_comments.pdf]

PEER REVIEW HISTORY

BMJ Open publishes all reviews undertaken for accepted manuscripts. Reviewers are asked to complete a checklist review form (**http://bmjopen.bmj.com/site/about/resources/checklist.pdf**) and are provided with free text boxes to elaborate on their assessment. These free text comments are reproduced below.

ARTICLE DETAILS

**TITLE (PROVISIONAL)**	TASCI: Transcutaneous tibial nerve stimulation in patients with Acute Spinal Cord Injury to prevent neurogenic detrusor overactivity: Protocol for a nationwide, randomised, sham-controlled, double-blind clinical trial
**AUTHORS**	Birkhäuser, Veronika; Liechti, Martina; Anderson, Collene; Bachmann, Lucas; Baumann, Sarah; Baumberger, Michael; Birder, Lori; Botter, Sander; Büeler, Silvan; Cruz, Célia; David, Gergely; Freund, Patrick; Friedl, Susanne; Gross, Oliver; Hund-Georgiadis, Margret; Husmann, Knut; Jordan, Xavier; Koschorke, Miriam; Leitner, Lorenz; Luca, Eugenia; Mehnert, Ulrich; Möhr, Sandra; Mohammadzada, Freschta; Monastyrskaya, Katia; Pfender, Nikolai; Pohl, Daniel; Sadri, Helen; Sartori, Andrea; Schubert, Martin; Sprengel, Kai; Stalder, Stephanie; Stoyanov, Jivko; Stress, Cornelia; Tatu, Aurora; Tawadros, Cécile; van der Lely, Stéphanie; Wöllner, Jens; Zubler, Veronika; Curt, Armin; Pannek, Jürgen; Brinkhof, Martin; Kessler, Thomas

This article was not externally reviewed at BMJ Open. Protocols that have been independently assessed prior to submission to BMJ Open are usually fast-tracked to publication on the grounds that further substantial changes will not be possible. This independent assessment will usually be external, independent review for both a substantial grant award from a non-commercial or government funder and ethics approval.
